# Date palm transcriptome analysis provides new insights on changes in response to high salt stress of colonized roots with the endophytic fungus *Piriformospora indica*

**DOI:** 10.3389/fpls.2024.1400215

**Published:** 2024-07-31

**Authors:** Manzoor Ahmad, Mughair Abdul Aziz, Miloofer Sabeem, M. Sangeeta Kutty, Sathesh K. Sivasankaran, Faical Brini, Ting Ting Xiao, Ikram Blilou, Khaled Masmoudi

**Affiliations:** ^1^ Department of Integrative Agriculture, College of Agriculture and Veterinary Medicine, United Arab Emirates University, Al Ain, United Arab Emirates; ^2^ Department of Vegetable Science, College of Agriculture, Kerala Agricultural University, Vellanikkara, Thrissur, India; ^3^ Division of Research, Innovation, and Impact, 106B Bond Life Sciences Center, University of Missouri, Columbia, MO, United States; ^4^ Biotechnology and Plant Improvement Laboratory, Centre of Biotechnology of Sfax (CBS)/University of Sfax, Sfax, Tunisia; ^5^ College of Plant Science and Technology, Beijing Key Laboratory for Agricultural Application and New Technique, Beijing University of Agriculture, Beijing, China; ^6^ King Abdullah University of Science and Technology (KAUST), Biological and Environmental Sciences and Engineering (BESE), Thuwal, Saudi Arabia

**Keywords:** transcriptome, *P. dactylifera*, *P. indica*, salinity, DEGs

## Abstract

Salinity is a significant threat that causes considerable yield losses in date palm. The root endophytic fungus *Piriformospora indica* has proven effective in providing salt stress tolerance to host plants. However, the underlying molecular mechanism facilitating the date palm’s response to *P. indica* inoculation, and its involvement in the salt stress tolerance, remains unknown. In this study, the colonization of *P. indica* on date palm seedlings exposed to saline conditions was observed through confocal microscopy, and its impact on gene expressions was evaluated using the transcriptomic analysis. Our findings show that *P. indica* colonization reinforced the cortical cells, prevented them from plasmolysis and cell death under salinity. The RNAseq analysis produced clean reads ranging from 62,040,451 to 3,652,095 across the treatment groups, successfully assembling into 30,600 annotated genes. Out of them, the number of differentially expressed genes (DEGs) varied across the treatments: i.e., 2523, 2031, and 1936 DEGs were upregulated, while 2323, 959, and 3546 were downregulated in Salt, Fungi, and Fungi+Salt groups, respectively. Furthermore, principal component analysis based on transcriptome profiles revealed discrete clustering of samples from different treatment groups. KEGG and GO pathways enrichment analysis highlighted variation in the number and types of enriched pathways among the treatments. Our study indicated variations in gene expression related to plant hormone biosynthesis and signal transduction (auxin, abscisic acid, gibberellin, and ethylene), ABC transporters, sodium/hydrogen exchanger, cation HKT transporter, transcription factors such as WRKY and MYBs, and the plant immune system (lipoxygenase and jasmonate) of the date palm seedlings. By characterizing the transcriptome of date palm roots under salt stress and with colonization of *P. indica*, the present findings provide valuable perspectives on the molecular mechanisms responsible for inducing salinity stress tolerance in plants.

## 1 Introduction

Salinity is a major challenge to agricultural crop growth, impacting approximately 20% of cultivated land and 33% of irrigated land globally (Shrivasata and Kumar, 2015). It disrupts crucial physiological and metabolic processes in plants, including photosynthesis, respiration, and protein synthesis (Athar et al., 2022). This disruption arises from ion toxicity, nutrient imbalances, decreased osmotic potential, and impaired water uptake, leading to damage to plant cell organelles (Abdul Aziz and Masmoudi, 2023a). In arid regions, increased groundwater salt levels have severely affected date palm production. Date palm, *Phoenix dactylifera*, employs intricate mechanisms involving sensing, signaling, and response to mitigate the adverse effects of salt stress (Ma et al., 2022). Therefore, enhancing date palm’s defense mechanisms of salinity tolerance is crucial for ensuring efficient date palm productivity in salt-affected regions.

Certain soil fungal species contribute to the enhancement of plant salt stress defense mechanisms, due to their adaptability to saline environments (Siddiqui et al., 2022). Endophytic mycorrhizal fungi establish mutually beneficial relationships with various terrestrial plant species, offering a potential solution for overcoming salinity stress. *Piriformospora indica*, belonging to the Sebacinales order, is an endosymbiont characterized as a harmless, axenically cultivable root endophyte. This fungus has the capability to colonize the roots of numerous higher plants, improving their growth by enhancing stress tolerance and disease resistance (Jisha et al., 2019). This has led to the speculation of their potential role as a symbiotic fungus for enhancing date palm’s tolerance to salinity stress.

A systematic investigation into the mechanisms underlying the date palm and *P. indica* colonization can be an essential way for improving the salt tolerance of date palm. The various pathways through which *P. indica* modulates plant physiological processes have been examined in different model interaction systems, such as *P. indica* colonization with *Arabidopsis thaliana* and *Hordeum vulgare* (Achatz et al., 2010; Qiang et al., 2012). Transcriptomic analyses in various studies have found differentially expressed genes (DEGs) in the roots of plants inoculated with *P. indica* under diverse stress conditions, including salinity stress (Ghaffari et al., 2016; Abdelaziz et al., 2019), drought stress (Zhang et al., 2018), early blight (Panda et al., 2019), and root-knot nematode infections (Atia et al., 2020). *P. indica* inoculation triggers the expression of host plant defense-related genes (*PR*, *LOX2*, and *ERF1*), abiotic stress-responsive genes (*DREB2A*, *CBL1*, and *RD29A*), and osmoprotectants like proline and glycine betaine (Waller et al., 2005; Zarea et al., 2012; Trivedi et al., 2013).

Thus, there is clear evidence that *P. indica* plays a crucial role in balancing the trade-off between the growth of host plants and their tolerance to salt stress by influencing various physiological aspects. The interaction between plants and this endophytic fungus is intricate and mutual, making it stimulating to reveal the transcriptomic alterations in date palms with the inoculation of *P. indica* in response to salt stress. Hence, it becomes imperative to develop novel molecular strategies aimed at enhancing salt stress tolerance in date palms through using endophytic fungi.

Limited transcriptome profiling studies have been conducted in non-model plants like date palm (Abdul Aziz and Masmoudi, 2024). Al-Dous et al. (2011) utilized Illumina GAII sequencing to assemble 58% of the date palm genome, predicting 25,059 genes. Bourgis et al. (2011) performed a comparative transcriptome study on oil and date palm mesocarps using pyrosequencing data from the Roche GS FLX Titanium platform. Additionally, pyrosequencing data contributed to transcriptomic profiles for date palm fruit development (Yin et al., 2012). Full-genome assemblies of date palm’s plastid (158,462 bp) and mitochondrion (715,001 bp) have been achieved (Yang et al., 2010; Fang et al., 2012). Al-Mssallem et al. (2013) conducted *de novo* transcriptome assembly using second and third generation sequencing technologies, revealing stress-related genes in date palm. Despite these efforts, the transcriptomic profiling of genes in response to salt stress for *P. indica* colonized date palm remains unexplored.

In our previous study, we have demonstrated that inoculation of date palm seedlings with the beneficial endophyte *P. indica* reduced the adverse impacts of salt stress (Sabeem et al., 2022). It improved the growth of date palm seedlings through the maintenance of ion balance, enhanced uptake of nutrients, and increased antioxidant activity. Hence, this study aims to enhance our understanding of *P. indica*-mediated salt tolerance in date palms through root transcriptome analysis. The main objectives include gaining a deeper understanding of *P. indica’s* potential to alleviate salt stress by profiling differentially expressed transcripts/genes in date palm during salt stress. Through RNA sequencing, the current investigation compares the transcriptomes of *P. indica*-colonized and non-colonized date palm roots under salt stress condition. Numerous salt-responsive date palm differentially expressed genes (DEGs) were identified, and their functional annotation was conducted. This transcriptome profiling contributes to our knowledge on how *P. indica* colonization in date palm roots aids plants to resist the increasing problem of salinity stress in arid regions.

## 2 Materials and methods

### 2.1 Plant material and growth conditions

Tissue culture seedlings of the Khalas variety of date palm were obtained from the tissue culture laboratory at UAE University and grown in ½ MS liquid medium (R core team, 2022). Before exposure to salinity stress treatments, seedlings were cultivated in a growth chamber for four weeks under controlled conditions, at a temperature of 25°C ± 2°C, a photoperiod consisting of 16 hours of light and 8 hours of darkness, and a relative humidity of 60%.

### 2.2 Fungal growth conditions, root colonization, and stress treatments

*P. indica* was cultivated on potato dextrose agar (PDA) medium (Sigma, St. Louis, USA) and left to incubate for 10 days at 28°C in darkness. Chlamydospores from the agar plate were introduced to 100 ml of Kaefer medium for liquid culture in 500 ml Erlenmeyer flasks. Furthermore, the flasks were placed on a rotary shaker, operating at 100 rpm, for 8 to 10 days at 28°C. Following centrifugation of the *P. indica* liquid culture, the harvested solution had its mycelium removed and was washed three times with sterile distilled water.

For the co-cultivation process, *P. indica* was combined with the roots of *in vitro*-grown Khalas date palm seedlings. This involved injecting 1% of a 500 µl diluted mycelial solution into the media surrounding the roots, with a subsequent gentle shaking of the mixture. The inoculated seedlings were placed in a growth chamber at 25°C with a photoperiod of 16 hours of light and 8 hours of darkness. To shield the root zones from light exposure, aluminum foil covered the bottom of the tubes containing the seedlings. After allowing *P. indica* to propagate within the roots of Khalas date palm seedlings for three weeks, four experimental groups were generated. Plant seedlings grown in normal water were classified as control. Seedlings grown in ½ MS medium with 250 mM NaCl are termed as the Salt group. Those cultivated in normal water and inoculated with *P. indica* are determined as the Fungi group. Seedlings grown in a ½ MS medium with 250 mM NaCl and inoculated with *P. indica* are categorized as the Fungi+Salt group in this study. Each experimental group was prepared in triplicates and samples from each replicate was used for subsequent transcriptomic and root structure analysis.

### 2.3 Root structure after colonization with *P. indica* and application of salt stress

Roots were collected after three weeks from both inoculated and non-inoculated seedlings cultivated in ½ MS liquid medium with the addition of 250 mM NaCl and under control conditions. These roots were embedded in 8% low melting agarose, and cross-sections were developed with a vibratome (Xiao et al., 2019), resulting in sections with a thickness of 150 µm. Consequently, the sections were stained using the cell wall dye SCRI Renaissance (Musielak et al., 2015).

Imaging of the sections was conducted using an LSM 880 Airyscan Confocal Microscope (Leica Microsystems, Wetzlar, Germany). The 405-nm laser was employed to visualize the cell wall stain, while the 488-nm laser was used for autofluorescence. Images were captured with a 2x0.8M17 Plan-Apochromat objective, and the tile scan function was employed to obtain the full field of view.

### 2.4 Total RNA extraction and cDNA library preparation for RNA-seq sequencing

Total RNA extraction from root samples of different treatments was carried out using the RNeasy total RNA isolation kit from Qiagen, following the manufacturer’s instructions. The concentration of RNA was determined using a NanoDrop1000 spectrophotometer from Thermo Scientific, USA. Approximately 3 µg of RNA per sample was used for cDNA synthesis. The TruSeq RNA Sample Prep Kit from Illumina, USA, was utilized to construct the cDNA libraries, following the manufacturer’s protocol, and indexed codes were introduced to associate sequences with each sample. Macrogen in Seoul, South Korea, performed paired-end sequencing for all libraries using an Illumina HiSeq X-ten platform.

### 2.5 Quality control and data analysis

The raw data in fastq file format were examined with the quality filtering procedures. Using the fastp tool, adaptor sequences and low-quality reads (with a quality score below Q20) were eliminated to obtain clean reads. These clean reads were used in subsequent analyses for downstream steps. For each sample, high-quality clean reads were aligned to the reference *P. dactylifera* genome (https://www.ncbi.nlm.nih.gov/bioproject/322046) using the HISAT2 tool. The feature counts tool was used to quantify transcript abundance and read counts (Liao et al., 2014). Moreover, gene expression levels were assessed through calculations using FPKM (fragments per kilobase of transcript sequence per million base pairs), a metric that normalizes expression by considering the transcript length and total mapped reads.

### 2.6 Differential expression analysis and functional annotation of transcripts

Differential expression analysis was performed using the DESeq2 R package (version 1.43.1) (Love et al., 2014). DEGs were identified by comparing the expression levels of all transcripts between the salt stressed and control groups. Genes exhibiting a fold change greater than 1 and a false discovery rate (FDR) value below 0.05 were considered differentially expressed.

The functions of the identified DEGs were annotated using the Gene Ontology (GO), Kyoto Encyclopedia of Genes and Genomes (KEGG), and Uniprot databases. Functional gene set enrichment analysis was conducted using the iDEP.96 software available at sdstate.edu. This analysis provided perceptions into the enriched biological processes and pathways associated with the identified DEGs, aiding in the interpretation of their functional significance in the context of the experimental conditions.

### 2.7 Data processing and visualization

The data analysis and visualization were carried out using R (version 4.3.1). Various packages were used for different aspects of analysis and visualization (R core team, 2022). Specifically, the Pheatmap package was utilized for generating heatmaps, the ggplot2 package for creating bar plots and volcano plots, and the ggVennDiagram package for generating Venn diagrams. The use of diverse R packages allowed for a thorough exploration and presentation of the results obtained from the differential expression analysis and functional annotation of genes.

## 3 Results

### 3.1 Root structural characterization of the date palm seedlings

**Figure 1:**
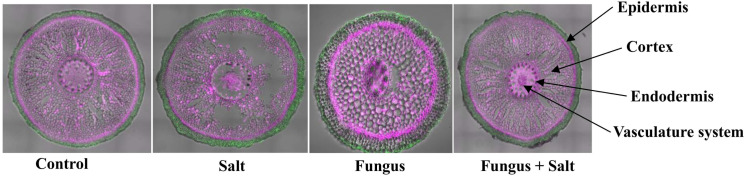
Anatomical structure of date seedlings roots. Confocal microscopy was used to examine the tissue anatomy of date palm roots colonized and non colonized with *P. indica* under control and salt stress conditions. The cell wall was stained with a pink color, while autofluorescence was represented by a green color.

To understand the cellular and subcellular responses of *P. indica* colonization in living date palm roots challenged with 250 mM NaCl, confocal microscopy was used to visualize tissue anatomy and reveal the consequences of salt treatment and the colonization of roots with *P. indica*. The *P. indica* colonized cortical cells reinforced the root architecture and prevented the plasmolysis and cell death of salt treated roots (**Figure 1**). The non-colonized and salt treated roots showed huge plasmolysis of the cortex cell layers, whereas the colonized roots with *P. indica* and salt-treated preserved the cortex cell layers from plasmolysis (**Figure 1**).

### 3.2 RNA sequencing analysis of the date palm seedlings root

**Figure 2:**
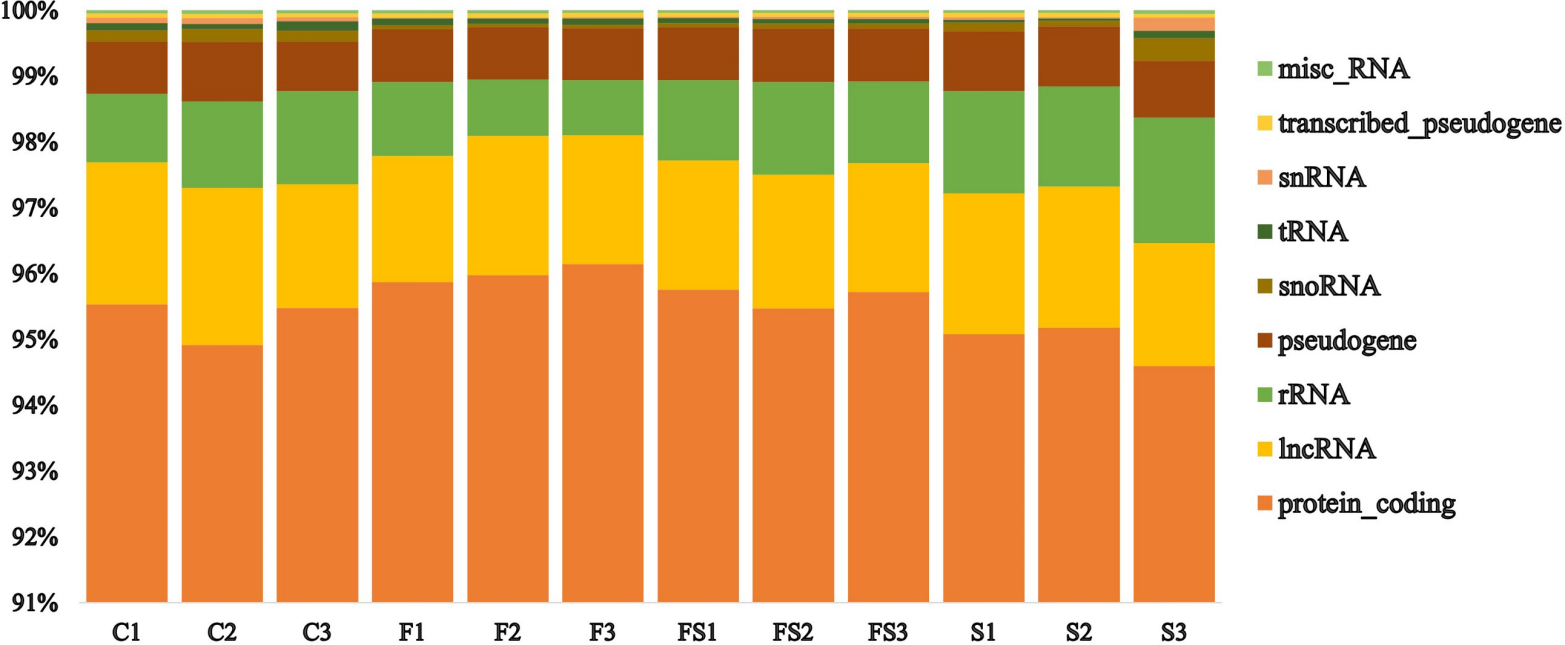
Distribution of the functionally assigned clean reads of the date palm seedlings root RNA Sequencing. The functionally assigned clean reads were distributed across various categories, including protein coding, long non-coding RNA (lncRNA), ribosomal RNA (rRNA), pseudogene, small nucleolar RNA (snoRNA), transfer RNA (tRNA), small nuclear RNA (snRNA), transcribed pseudogene, and miscellaneous RNA (misc_RNA), in different treatments. The treatments were categorized as follows: C: control), F: *P. indica* colonized date palm seedlings under control condition, FS: *P. indica* colonized date palms under salt stress condition, and S: non-colonized date palm seedlings under salt stress conditions.

A transcriptomic analysis was conducted to investigate the molecular mechanisms underlying *P. indica*-mediated salt tolerance or adaptation in date palm seedlings under salinity stress. The sequencing results yielded raw reads ranging from 67,295,188 to 39,977,852 (**Supplementary Table S1**). After the elimination of low-quality reads and contaminants, the clean reads from all samples ranged from 62,040,451 to 3,652,095 (**Supplementary Table S1**). The control group had the highest number of both raw and clean sequencing reads, while the Fungi+Salt treatment group had the lowest (**Supplementary Table S1**). Similarly, the control group exhibited the highest number of mapped reads at 5,254,737, whereas the Fungi+Salt group had the lowest reads (3,275,435). Approximately, 30,600 functional genes were annotated from the clean reads, with over 94% successfully assigned to protein-coding genes across all samples (**Figure 2**). Particularly, the Fungi+Salt group showed a relatively higher number of protein-coding genes (94% to 96%) compared to other treatments.

### 3.3 Identification and evaluation of DEGs

**Figure 3:**
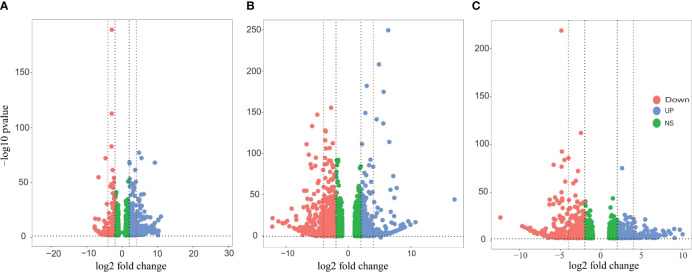
Volcano plots of the DEGs in the different treatment groups of the date palm seedlings. DEGs were identified using DESeq2 R package (version 1.43.1). The vertical axis represents the statistical significance of the difference in gene expression. Blue dots indicate the upregulated DEGs, the red dot signifies downregulated DEGs, and the green dot shows the non-significant DEGs. **(A)** Visualization of the DEGS in the date palm seedlings inoculated with *P. indica* under control condition. **(B)** Visualization of the DEGS in the non-inoculated date palm seedlings inoculated under salt stress condition. **(C)** Visualization of the DEGS in the date palm seedlings inoculated with *P. indica* under salt stress condition. The analysis revealed significant variation in the expression pattern of DEGs across the treatments.

In order to understand how *P. indica* colonization enhances salt tolerance in date palms, we analyzed the DEGs in the established experimental groups. The transcriptomic profile of the control group (without salt and uncolonized) was used as a reference for both salt and fungi treatments. However, for the Fungi+Salt group, the salt treatment was used as a reference transcriptome. DEGs were examined based on a fold change of ≥ 1 and an FDR of ≤ 0.05 in response to salt stress and *P. indica* colonization in date palm roots. In the Salt, Fungi, and Fungi+Salt treatments, a total of 25,902, 23,351, and 25,679 DEGs were identified, respectively (**Figures 3A–C**; **Supplementary Table S2**).

**Figure 4:**
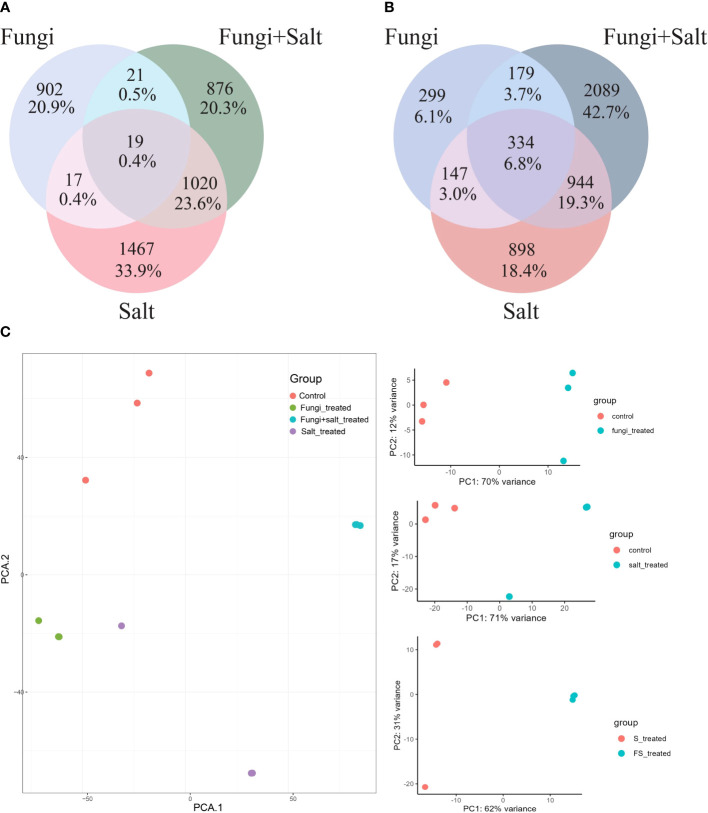
Variances and similarities in the transcriptomic profile of DEGs across the treatment groups. DEGs were identified using DESeq2 R package (version 1.43.1). In this analysis only DEGs with fold change ≥ 1 and ≤ -1 are included. **(A)** Venn diagram displaying the shared and treatment specific upregulated DEGs among the treatments. **(B)** Venn diagram showing the shared and treatment-specific downregulated DEGs across the treatments. **(C)** PCA based on the Fragments Per Kilobase Million (FPKM) values demonstrating the clustering of samples from the different experimental groups.

Among the treatments, a high number of upregulated DEGs were observed in the Salt group (2523) followed by fungi group (2301), whereas the lowest number (1936) was observed in Fungi+Salt group (**Supplementary Table S2**). Conversely, Fungi+Salt treatment displayed the highest count of downregulated DEGs (3546), trailed by the Salt group (2323), whereas only 959 downregulated DEGs were identified in the fungi treated group. The variation in DEGs between the treatments showed how the colonization of *P. indica* influenced the date palm’s functional responses under normal condition and salinity stress. Moreover, it is worth noting that, among the upregulated DEGs, only 19 genes (0.4%) were observed to be present in all treatment groups (**Figure 4A**). In contrast, most upregulated DEGs exhibited treatment-specific expression patterns, as depicted in the Venn diagram (**Figure 4A**). Correspondingly, when considering the downregulated DEGs, it is notable that just 334 genes (6.8%) were detected consistently across all treatments. Moreover, the salt group and Fungi+salt group exhibit the largest proportion of upregulated and downregulated DEGs, with 1020 (23.0%) and 944 (19.3%) DEGs respectively (**Figures 4A, B**). The PCA analysis based on the transcriptome profile showed the distinct clustering of the samples of different treatments, which further explained the differences in functional responses of date palm under different stress conditions (**Figure 4C**).

### 3.4 Pathway enrichment analysis of the transcriptome of the colonized date palm with *P. indica*

**Figure 5:**
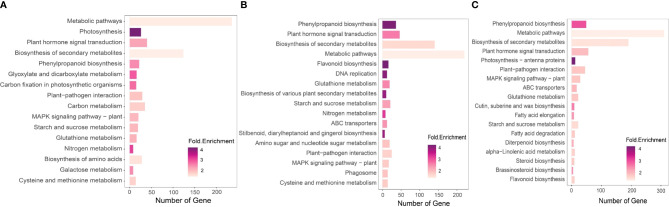
KEGG pathway enrichment analysis of the DEGs of the experimental date palm groups. We performed KEGG pathway enrichment analysis using iDEP.96 available at iDEP.96 (sdstate.edu). The analysis revealed variation in the number and types of enriched pathways in the experimental groups. **(A)** Non-inoculated date palm seedlings under salt stress (Salt group) **(B)** *P. indica* inoculated date palm seedlings under salt stress (Fungi) **(C)** *P. inidca* inoculated date palm seedling under the control condition (Fungi+Salt).

Various source databases, such as NCBI, GO, KEGG, and UniProt, were used for pathway enrichment analysis and functional gene annotation. The analysis results indicate variations in gene counts and fold changes associated with different pathways in the established treatments (**Figures 5A–C**).

**Figure 6:**
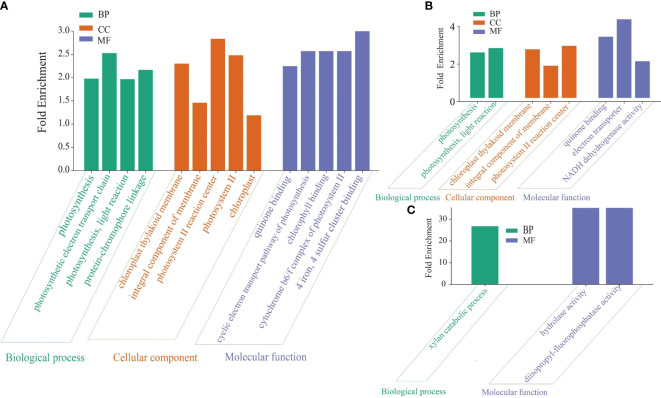
GO pathway of the DEGs in the date palm seedlings treatment conditions. The GO pathway enrichment analysis was performed using iDEP.96 tool available at iDEP.96 (sdstate.edu). The analysis showed substantial variations in enriched pathways across the experimental groups. **(A)** *P. indica* inoculated date palm seedlings under salt stress condition (Fungi+Salt group). **(B)** Non-inoculated date palm seedlings under salt stress condition (Salt group). **(C)** *P. indica* inoculated seedlings under the control condition (Fungi group).

For the pathway enrichment analysis and functional gene annotation, different source databases were used, including the NCBI, GO, KEGG and UniProt. Overall, the analysis findings show variations in gene counts and fold changes of genes associated with different pathways in the established treatments. For example, we observed a higher number of enriched KEGG pathways in the Salt+Fungi group (18), followed by the Fungi group (17) (**Figure 5A**), whereas the lowest number (16) enriched KEGG pathways were observed in the Salt group. Additionally, the different groups exhibited variations in the enriched pathways. For instance, the Fungi group showed significant enrichment in pathways, including DNA replication (3.86 folds), phagosome (1.75 folds), cysteine, and methionine metabolism (1.65 folds), stilbenoid, diarylheptanoid and gingerol biosynthesis (3.70 folds), starch and sucrose metabolism (2.14 folds), as well as biosynthesis of various plant secondary metabolites (3.76 folds) (**Figure 5B**). Some pathways, including fatty acid elongation (2.91 folds), fatty acid degradation (2.00 folds), diterpenoid biosynthesis (2.46 folds), steroid biosynthesis (1.99 folds), alpha-Linolenic acid metabolism (1.85 folds), and brassinosteroid biosynthesis (2.97 folds), were enriched in the Fungi+Salt treated group (**Figure 5C**). Additionally, we found that the pathways associated with ABC transporters were present in both the Fungi+Salt and Fungi groups but were not detected in the Salt group. The GO pathway enrichment analysis revealed a higher number of enriched pathways in the Fungi+Salt group (**Figure 6A**), followed by the Salt group (**Figure 6B**). The Fungi treated group exhibited a lower number of enriched pathways (3) compared to Fungi+Salt (14) and Salt (8) groups (**Figure 6C**). In a similar way, the Uniprot pathway enrichment analysis revealed a clear and discernible pattern of enriched pathways across the various experimental treatments. The discrepancy in enriched pathways demonstrates the impact of *P. indica* colonization on the functional responses of date palms under salinity stress (**Supplementary Figure S1**).

### 3.5 Impact of *P. indica* inoculation on the date palm hormone related genes

**Figure 7:**
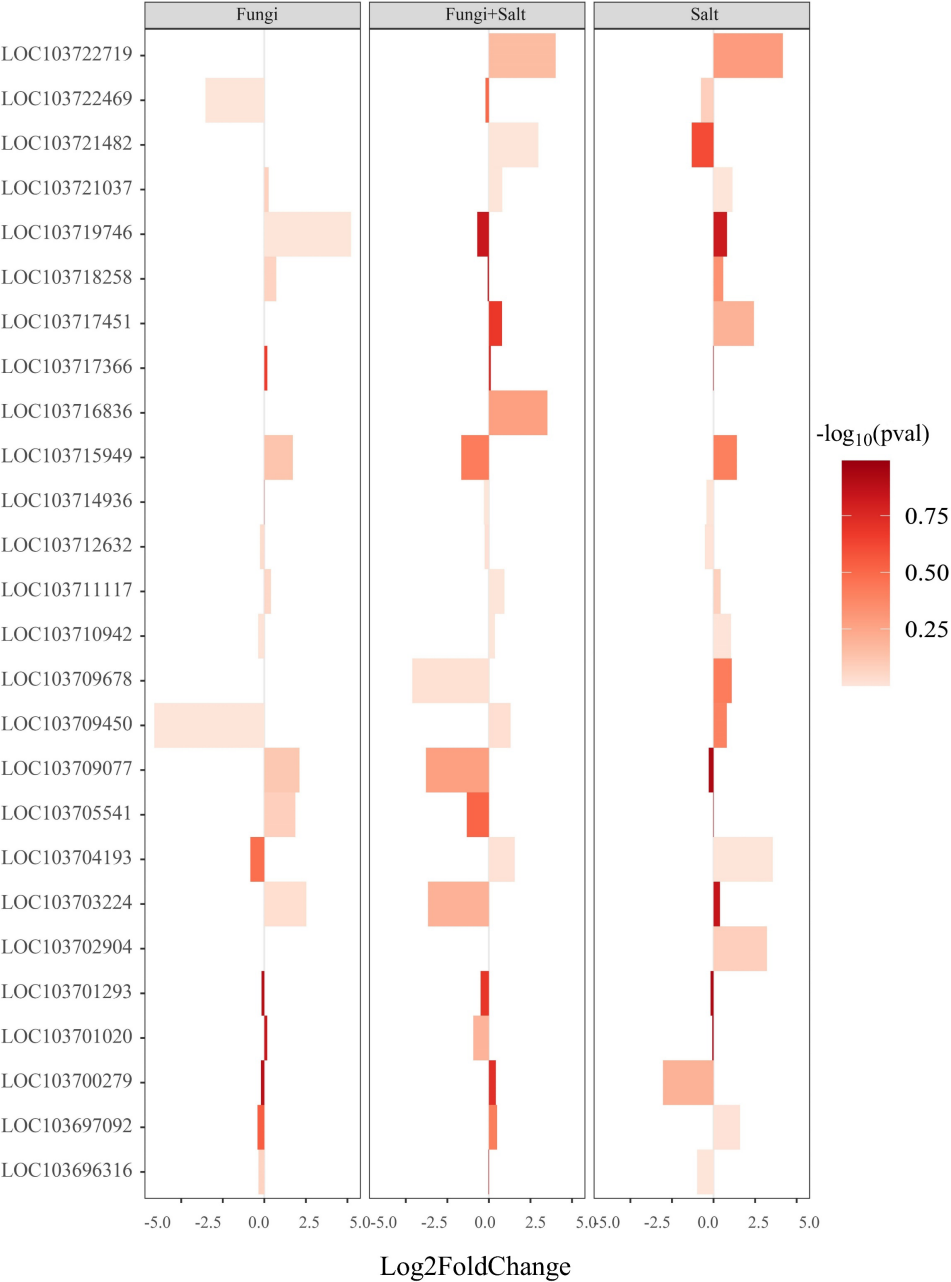
Bar plot showing the expression of DEGs associated with gibberellin biosynthesis and catabolism. DEGs were identified using DESeq2 R package (version 1.43.1). DEGs involved in gibberellin biosynthesis and catabolism were retrieved from the DEGs profile of each treatment and a heated bar plot were generated using ggplot2 package in R. The coordinates of the figure show the statistics of genes expression. The bar color intensity shows the increase and decrease of *P* value in the form of -log_10_. Fungi: *P. indica* inoculated under control condition, Fungi+salt: *P. indica* inoculated under salt stress condition, and Salt: Non- inoculated salt stress condition.

Plant hormones regulation is important for plant cell cycle, plant growth and stress responses. The analysis of DEGs related to plant hormones, including auxin, gibberellin, and abscisic acid, was conducted in the transcriptome of each experimental group. The results revealed specific variations in the expression profiles of genes associated with hormone regulation across the treatments. Particularly, a larger number of DEGs related to gibberellin biosynthesis were identified in the *P. indica* inoculated date palm seedlings under the salt stress condition (28), followed by the non-inoculated seedlings under the salt stress condition (25), while the *P. indica* inoculation under the control condition exhibited the lowest number (18) (**Figure 7**; **Supplementary Table S3**). Particularly, genes associated with gibberellin 2-beta-dioxygenase 8 (1.10 folds), gibberellin 2-beta-dioxygenase 2 (3.64 folds), and gibberellin 3-beta-dioxygenase 1-like (2.47 folds) were significantly upregulated in the *P. indica* inoculated date palms under salt stress treatment. Moreover, genes including gibberellin 2-beta-dioxygenase 1 (1.74 folds), gibberellin 20-oxidase-like protein (1.86 folds), gibberellin-regulated protein 5 (2.11 folds), and gibberellin-regulated protein 6 (5.46 folds) were significantly upregulated in the *P. indica* inoculated date palm seedling under control condition but downregulated in the *P. indica* inoculated date palm seedling under salt stress condition (**Figure 7**).

Similarly, significant differences were observed in the DEGs associated with auxin and abscisic acid-responsive proteins across the treatments (**Supplementary Figures S2**, **S3**). The *P. inidca* inoculated date palm seedlings under the salt stress condition showed a higher abundance of DEGs related to auxin (75) and abscisic acid (78), followed by the non-inoculated salt stress condition (79 and 77), and the *P. indica* inoculated under the control condition (57 and 44) (**Supplementary Figures S2**, **S3**; **Supplementary Tables S4**, **S5**). These findings highlight the intricate and treatment-specific regulation of plant hormone-related genes, emphasizing their importance in plant cell cycle, growth, and stress responses.

### 3.6 *P. indica* inoculation alters the expression pattern of transcription factors

**Figure 8:**
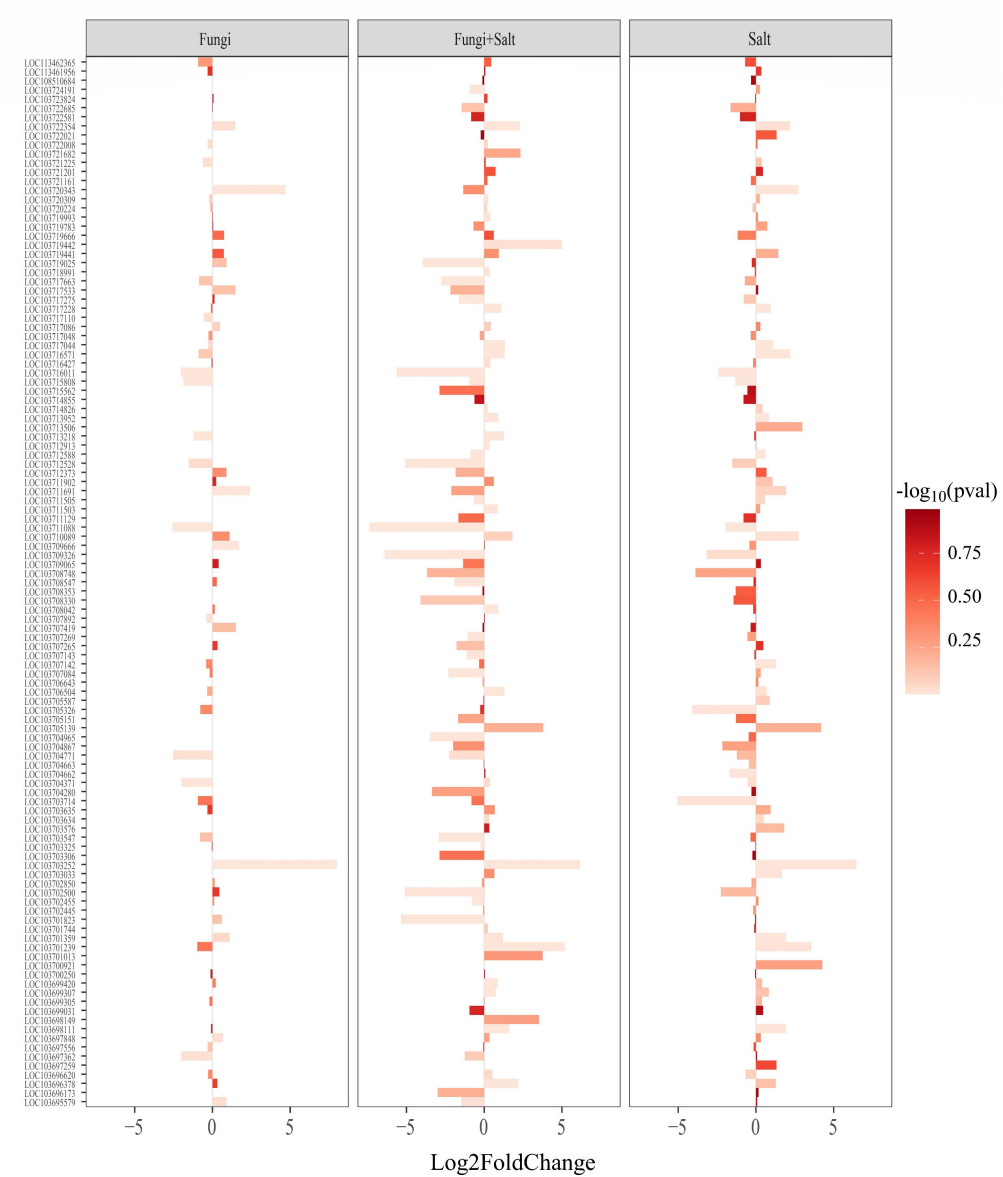
Bar plot showing the expression pattern of ethylene-responsive transcription factors (ERFs) across the treatments. DEGs were identified using DESeq2 R package (version 1.43.1). DEGs related to ERFs were retrieved from the DEGs profile of each treatment and a heated bar plot were generated using ggplot2 package in R. The bar coordinates show the statistics of ERFs expression. The bar color intensity shows the increase and decrease of *P* value in the form of -log_10_. Fungi: *P. indica* inoculated under control condition, Fungi+salt: *P. indica* inoculated under salt stress condition, and Salt: Non- inoculated salt stress condition.

In this study, stress tolerance was explored with a focus on transcription factors, crucial regulatory components in stress-responsive pathways. Specifically, three families of stress-responsive transcription factors—ethylene-responsive transcription factors (ERFs), WRKYs, and MYBs—were examined in the transcriptome of the treatment groups. The results revealed significant variations in the number and expression patterns of DEGs associated with these transcription factor families across the treatments (**Figure 8**).

Among the three families, the *P. indica* inoculated date palm seedlings under the salt stress condition showed the highest number of ERFs (111), followed closely by the non-inoculated seedlings under the salt stress condition with 110 ERFs, while the *P. indica* inoculated date palm seedling under the control condition showed 68 ERFs (**Figure 8**; **Supplementary Table S6**). Furthermore, the expression of ERFs within each treatment varied, for instance, in the *P. indica* inoculated date palm seedlings under the salt stress, 17 out of 111 ERFs (15.3%) were upregulated, and 34 (30.6%) were downregulated. Similarly, in the non-inoculated salt stress condition and inoculated control condition, 21 (19.1%) and 9 (13.2%) ERFs were upregulated, while 18 (16.3%) and 8 (11.7%) were downregulated, respectively (**Supplementary Table S6**).

In the other two transcription factor families (WRKYs and MYBs), significant variations were observed in both the number and expression patterns across the experimental treatments. Particularly, the *P. indica* inoculated under the salt stress group had the highest number of WRKYs (88) and MYBs (34), followed by the non-inoculated salt stress group with 85 WRKYs and 33 MYBs, and the *P. indica* inoculated under the control condition with 71 WRKYs and 30 MYBs (**Supplementary Figures S4**, **S5**; **Supplementary Tables S7**, **S8**). These findings indicate the intricate regulatory role of transcription factors in stress responses and highlight treatment-specific differences in their expression profiles.

### 3.7 *P. indica* inoculation modulates the expression of immune and transporter genes

The inherent immune system of plants relies on various components, including salicylic acid, jasmonate, and ethylene, which play pivotal roles in influencing both harmful pathogens and symbiotic organisms. Additionally, lipoxygenases contribute by converting linoleic or alpha-linolenic acid into (9S)- and (13S)-hydroperoxides, serving as precursors for various oxylipins involved in plant defense. Transcriptomic analysis indicated that the colonization of *P. indica* led to alterations in the abundance and expression of genes related to plant defense.

The expression of genes involved in jasmonate synthesis exhibited differences among the experimental groups (**Supplementary Figure S6**; **Supplementary Table S9**). The non-inoculated salt stress group showed the highest number of DEGs related to jasmonate (4), followed by *P. indica* inoculated under the salt stress condition (3), and *P. indica* inoculated under the control condition (2). Similarly, a total of 8 DEGs related to lipoxygenases were identified in the transcriptome of the experimental groups. Specifically, a significant variation was observed in the expression patterns of these DEGs across the treatments (**Supplementary Figure S6**; **Supplementary Table S10**). In the *P. indica* inoculated seedlings under the salt stress condition, 6 out of the jasmonate-related DEGs (75%) were downregulated, while in the non-inoculated salt stress condition, only 3 (37.5%) were downregulated.

Transporter genes, including ABC transporters, HKT, and sodium/hydrogen exchangers, play a crucial role as transporter proteins in plant stress responses and various physiological processes (Sabeem et al., 2022). In this study, three differentially expressed genes (DEGs) related to the HKT gene, specifically HKT1, HKT6, and HKT8, along with their isoforms, were identified in the transcriptome of treatment condition (**Supplementary Figure S7**; **Supplementary Table S** **11**). These genes exhibited a differential expression pattern across the treatments. Moreover, all HKT-related genes were upregulated in the *P. indica* inoculation under control condition, while they were downregulated in the *P. indica* inoculated under the salt stress condition.

Similarly, four DEGs related to sodium/hydrogen exchangers and their isoforms were identified with varying expression levels in different treatments (**Supplementary Figure S8**; **Supplementary Table S12**). In the *P. indica* inoculation under the control condition, most of the sodium/hydrogen exchangers showed downregulation. However, a different expression pattern was observed in the *P. indica* inoculation under the salt stress and non-inoculated salt stress condition, except for one isoform (LOC103713094: sodium/hydrogen exchanger 2-like), which exhibited upregulation in both groups, (1.28 folds) and (1.11 folds), respectively.

ABC transporters play an essential role in plant physiology by facilitating the movement of various substances across cell membranes. This involvement is critical for essential processes such as nutrient absorption, hormone signaling, detoxification, and plant-microbe interactions, among others. Ultimately, these transporters significantly impact plant growth, development, and stress responses. In the present study, 109, 107, and 97 DEGs related to ABC transporters genes and their isoforms were identified in the Salt group, Fungi+Salt group and Fungi group, respectively (**Supplementary Figure S8**; **Supplementary Table S13**). The Salt group shared the highest proportion of upregulation (23.8%) followed by Salt+Fungi (21.4%) and Fungi (17.5%). On the other side, Fungi+Salt group exhibited the largest proportion of downregulated DEGs (14.5%), followed by Salt group (11%) and Fungi group (5.1%). In addition, we observed differences in the ABC transporters linked to DEGs among the treatments (**Supplementary Table S1** **3**).

## 4 Discussion

The endophytic fungus, *P. indica*, plays a crucial role in effectively modulating plant responses to salt stress and reducing its adverse effects on plant functioning. Transcriptomic analysis serves as a gold standard proxy for exploring stress-responsive genes and understanding the molecular mechanisms of stress tolerance in plants (Abdul Aziz et al., 2022). In this study, the roots of date palm seedlings were inoculated with *P. indica*, and RNAseq was conducted after successful colonization, with the goal of revealing the molecular mechanisms underlying *P. indica*-mediated salt tolerance in date palms. The study showed the formation of a symbiotic interaction between the *P. indica* and date palm roots due to its ability to colonize a broad range of host, previously found with barley (Baltruschat et al., 2008), and rice (Jogawat et al., 2013).

An initial assessment of root sections using confocal microscopy displayed significant plasmolysis of the cortex cell layers in non-colonized salt-treated roots. In contrast, the roots colonized by *P. indica* exposed to salt treatment exhibited reinforced and preserved cortex cell layers, preventing plasmolysis. This observation displays the colonization and interaction of *P. indica* with the date palm roots, suggesting a potential protective role of *P. indica* in maintaining the structural integrity of root cells under salt stress conditions (Gill et al., 2016). Likewise, it was reported by Ali et al. (2022) that the endophytic fungus *Stemphylium lycopresici* inoculated to maize roots under saline condition (Gill et al., 2016), enhanced the root hair development, lowered the formation of lysogenic aerenchyma and decreased the Na^+^/K^+^ ratio in maize plants.

The results of RNAseq showed alterations in gene expression within the roots of date palm CV Khalas colonized by *P. indica*, enhancing its tolerance to salt stress. The observed changes in gene expression patterns between *P. indica*-colonized and non-colonized date palm seedling roots under salt stress highlight the significant influence of *P. indica* colonization on plant gene expression. These findings associated with existing studies on the impact of endophytic microorganisms on host plant gene expression under abiotic stresses (Masmoudi et al., 2021). For instance, Cheng et al. (2021) reported significant gene alterations in the leaves of *Achnatherum inebrians* under salt stress induced by the fungus *Epichloe gansuensis*. In contrast, the introduction of *Epichloe coenophiala* into tall fescue resulted in minimal genetic alterations, with the DEGs primarily involved in the plant’s response to abiotic stress (Dinkins et al., 2017). Similarly, the analysis of the whole transcriptome of rice plants revealed that approximately 0.5% of all genes experienced expression level changes following inoculation with *Glomus intraradices* (Güimil et al., 2005). Gazara et al. (2020) reported *P. indica*-induced gene modifications in rice plants exposed to salinity. Furthermore, Rho et al. (2018) conducted a meta-analysis, indicating that incorporating endophytes, particularly *P. indica*, positively impacted plant growth and the ability to withstand abiotic stresses such as salinity, drought, and nitrogen deficiency. These collective findings indicated the potential of *P. indica* in influencing the molecular responses of plants to environmental stresses, including salt stress, thereby enhancing their tolerance. Similarly, our results showed the salt stress tolerance was conferred in date palm by *P. indica* inoculation via the expression of defense-related genes.

The pathway enrichment analysis conducted in this study, with a specific focus on KEGG pathways, revealed significant differences in enriched pathways when comparing the transcriptomes of *P. indica*-colonized roots to non-colonized roots (salt stress) of date palm seedlings. These differences highlight the selective involvement of the *P. indica* influenced expressed in different KEGG pathways, contributing to increased resilience to salinity stress. For example, there was a significant upregulation of genes involved in phenylpropanoid biosynthesis in the *P. indica* inoculated date palm seedlings under the salt stress group (fold change = 3.95) and inoculated control condition (fold change = 3.97), compared to the non-inoculated salt stress treatment (fold change = 2.48). This indicates the beneficial role of *P. indica* colonization in elevating salt stress. Phenylpropanoids, synthesized from phenylalanine in plants, are secondary metabolites crucial for enhancing plant’s ability to withstand various biotic and abiotic stresses (Korkina, 2007). Additionally, the upregulation of genes associated with flavonoid biosynthesis in *P. indica*-colonized roots (control and salt stress conditions) suggests a potential enhancement of the antioxidant capacity of date palms against reactive oxygen species (ROS) produced during salt stress. Flavonoids, known as low molecular weight phenolic compounds, possess the ability to scavenge ROS generated in plants facing biotic or abiotic stress conditions (Shomali et al., 2022).

The transcriptome analysis of date palm roots during the interaction with *P. indica* revealed a stimulation of phytohormones such as gibberellin, auxin, and abscisic acid biosynthesis and signal transduction. The expression of genes involved in gibberellin biosynthesis displayed significant differences between the roots of *P. indica*-colonized and non-colonized plants. The higher expression of gibberellin 2-beta-dioxygenase 8, gibberellin 3-beta-dioxygenase 1, and gibberellin 2-beta dioxygenase 2 in the roots of *P. indica*-colonized date palm seedlings suggests the beneficial effect of *P. indica* on plant growth under salt stress. Particularly, the presence of gibberellin 2-beta-dioxygenase 8, an enzyme involved in the hydroxylation of gibberellin precursors, exclusively in the *P. indica* inoculation under the salt stress group is essential. The role of gibberellin in interactions of endophytes with roots is well known. The earlier studies on *P. indica* mainly focused on the activation of innate immune responses through gibberellin synthesis in plants. It has been displayed that *P. indica* supported the rice plants to survive root herbivory, and gibberellin functioned as a signal and interaction component for the inducible plant tolerance against the stress conditions (Cosme et al., 2016). In barley mutants, the impaired synthesis of gibberellins affected the perception and reduced *P. indica* colonization which indicated that gibberellin acts as a modulator of the basal defense of the roots (Schäfer et al., 2009). Previous research has further showed that plants with elevated levels of gibberellin catabolism genes can better withstand challenging environments such as drought and high salinity (Zhou and Underhill, 2016).

Similarly, auxin and abscisic acid are vital plant hormones that play fundamental roles in plant metabolism, growth, and development (Bouzroud et al., 2019). The results indicate that *P. indica* colonization alters the gene expression of auxin and abscisic acid-related genes. Auxins such as IAA are not only involved in key developmental processes but also plays a role in stress defense responses (Naser and Shani, 2016). Therefore, it was not surprising in our study that root-interaction of *P. indica* with date palm seedlings interfered with its auxin metabolism or signaling, which stimulated their growth and developmental processes. Abscisic acid-intensive 5 protein, which participates in drought stress response, showed a significant upregulation (fold change = 6.43) in the *P. indica*-colonized salt-treated date palm roots. In general, ABA stimulates Arbuscular Mycorrhiza (AM) symbiosis but the impact of this hormone is largely dependent on the developmental stage of the interaction and the conditions of stress (López-Ráez, 2016). Mainly, ABA plays its role when plants and their interacting symbionts are under the stress conditions, specifically osmotic stress. Therefore, the variations in the gene expression profile related to auxins and ABA signify the beneficial effect of *P. indica* colonization on date palm growth under salt stress. The modulation of hormone-related genes by *P. indica* suggests a potential mechanism through which the fungus enhances the salt tolerance of date palm seedlings.

Transcription factors, including ERFs, WRKYs, and MYBs, exert a significant impact on regulating the biosynthesis and signaling of stress-related hormones such as ethylene, abscisic acid, jasmonate, and salicylic acid (Abdul Aziz and Masmoudi, 2023b; Zhou et al., 2022). It has been reported that inoculation of *P. indica* with barley seedlings upregulated specific genes such as WRKY transcription factors (Molitor et al., 2011). This suggests WRKY a significant interaction factor in the *P. indica’s* inoculation with date palm seedlings. Our data analysis revealed a significant difference in the expression pattern of transcription factors between *P. indica*-colonized and non-colonized roots of date palm seedlings. These variations in transcription factor expression could potentially influence the expression of key genes involved in the biosynthesis and signaling of stress-related hormones that may play a role in the salinity tolerance of date palm seedlings (Abdul Aziz et al., 2021).

For example, ethylene, a crucial phytohormone with vital roles in plant physiological processes, including growth, development, and stress responses, exhibited higher expression of 1-aminocyclopropane-1-carboxylate oxidase 1 in the *P. indica*-colonized groups (control and salt stress) compared to non-colonized roots (salt stress) (**Supplementary Figure S9**). 1-aminocyclopropane-1-carboxylate oxidase 1 is an essential enzyme in ethylene biosynthesis and serves as a rate-limiting factor. Therefore, our data here showed that *P. indica* induces ethylene synthesis in date roots after inoculation, which suggests that ethylene signaling is required for symbiotic interaction between them. It has been found that impaired ethylene signaling resulted in reduced root colonization in Arabidopsis (Khatabi et al., 2012). Furthermore, previous research indicated that the colonization of barley roots by *P. indica* led to the upregulation of 1-aminocyclopropane-1-carboxylate oxidase (Schäfer et al., 2009). Similarly, genes involved in jasmonate biosynthesis, a major component of the plant innate immune system, exhibited altered expressions in *P. indica*-colonized roots compared to non-colonized date palm roots. These findings are related to previous studies, suggesting that *P. indica* colonization leads to the downregulation of genes involved in jasmonate biosynthesis, resulting in a lower immune response (Schäfer et al., 2009). This downregulation facilitates successful colonization and the establishment of a symbiotic association.

## 5 Conclusion

This study provides evidence that the colonization of *P. indica*, a fungal endophyte, in date palm roots contributes to the reinforcement of root architecture, prevention of plasmolysis, and reduction in cell death under high salinity conditions. Transcriptome analysis of the roots revealed that *P. indica* colonization led to the upregulation of numerous genes associated with salt stress and signaling pathways. Results from the GO and KEGG analyses displayed DEGs associated with various biological processes crucial for responding to salinity tolerance. Furthermore, the modulation of gene expression related to gibberellin, auxin, ethylene biosynthesis, and signaling, and with varied expression patterns of downstream transcription factors, is anticipated to exhibit a strong correlation with salt stress tolerance. The findings of this study provide essential insights into the molecular mechanisms underlying *P. indica*-mediated salt tolerance in date palm. It lays the groundwork for future research on the interaction between date palm and *P. indica*, as well as other endophytic microorganisms, an area of study that has received limited attention.

## Data availability statement

The original contributions presented in the study are included in the article/**Supplementary Material**, further inquiries can be directed to the corresponding author.

## Author contributions

MA: Formal analysis, Software, Visualization, Writing – original draft, Writing – review & editing. MAA: Investigation, Methodology, Writing – review & editing. MS: Methodology, Writing – review & editing. MSK: Investigation, Methodology, Writing – review & editing. SKS: Formal analysis, Writing – review & editing. FB: Investigation, Writing – review & editing. TX: Methodology, Writing – review & editing. IB: Investigation, Methodology, Writing – review & editing. KM: Conceptualization, Funding acquisition, Resources, Supervision, Validation, Writing – original draft, Writing – review & editing.
