# Supplementary material for: Date palm transcriptome analysis provides new insights on changes in response to high salt stress of colonized roots with the endophytic fungus Piriformospora indica
Source: Front Plant Sci. 2024 Jul 31;15:1400215. doi: 10.3389/fpls.2024.1400215 (PMC11322345; doi:10.3389/fpls.2024.1400215)
Supplement: Supplementary file 1 [file DataSheet_1.docx]

Supplementary Material

# Supplementary Figures and Tables

##
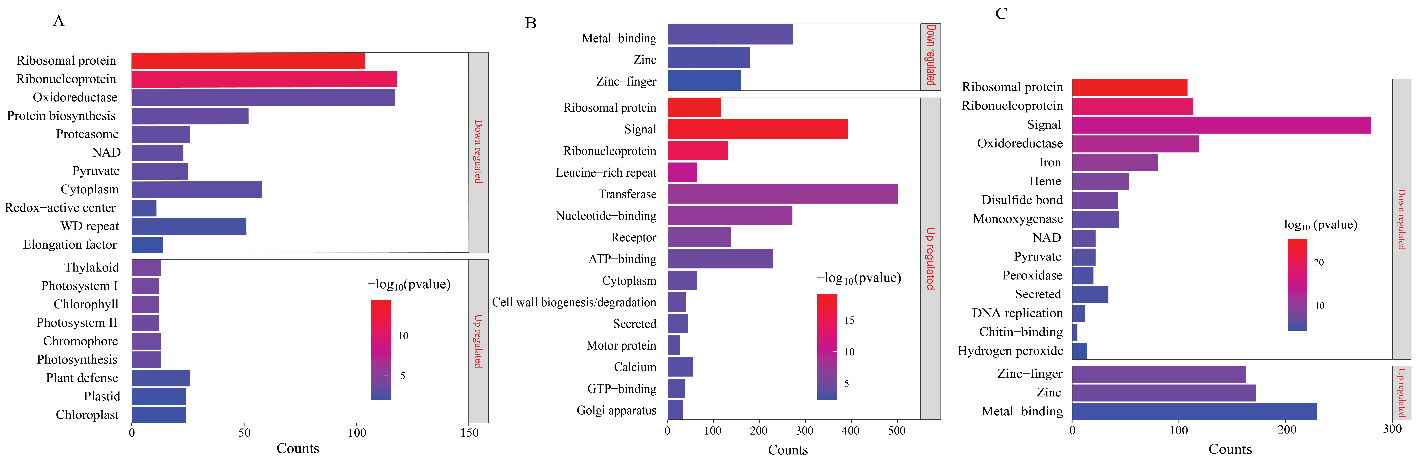
Supplementary Figures

**Supplementary Figure 1:** UniProt pathway enrichment analysis of the DEGs from the different treatment groups. (A) Non-inoculated date palm seedlings under salinity stress. (B) *P. indica* inoculated date palm seedlings under the salinity stress. (C) *P. indica* inoculated date palm seedlings under the control condition.


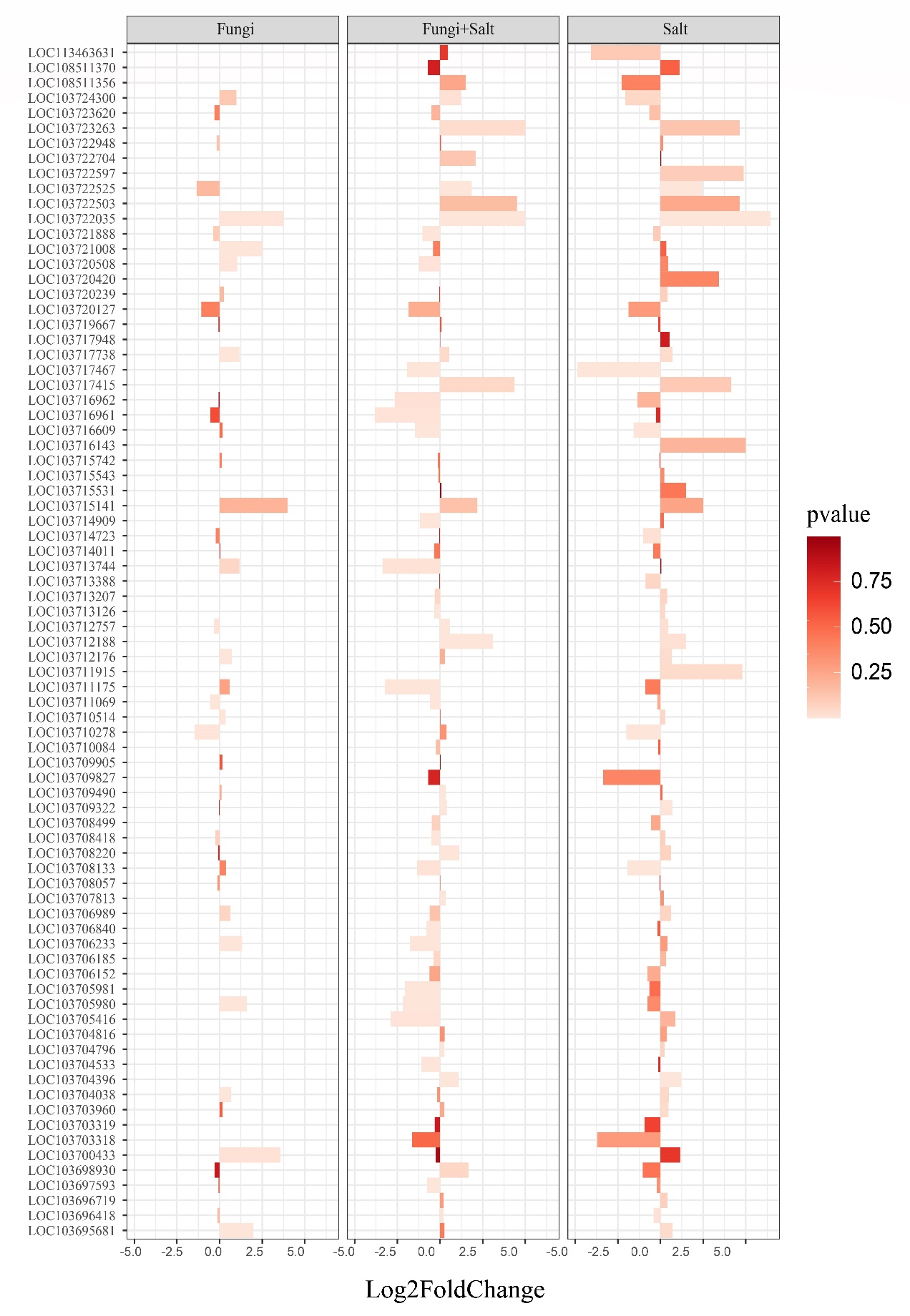


**Supplementary Figure 2:** Expression of DEGs associated with auxin. The bar plot displays the DEGs involved in auxin biosynthesis for different treatment conditions. The coordinates of the figure represent the statistics of genes expression.


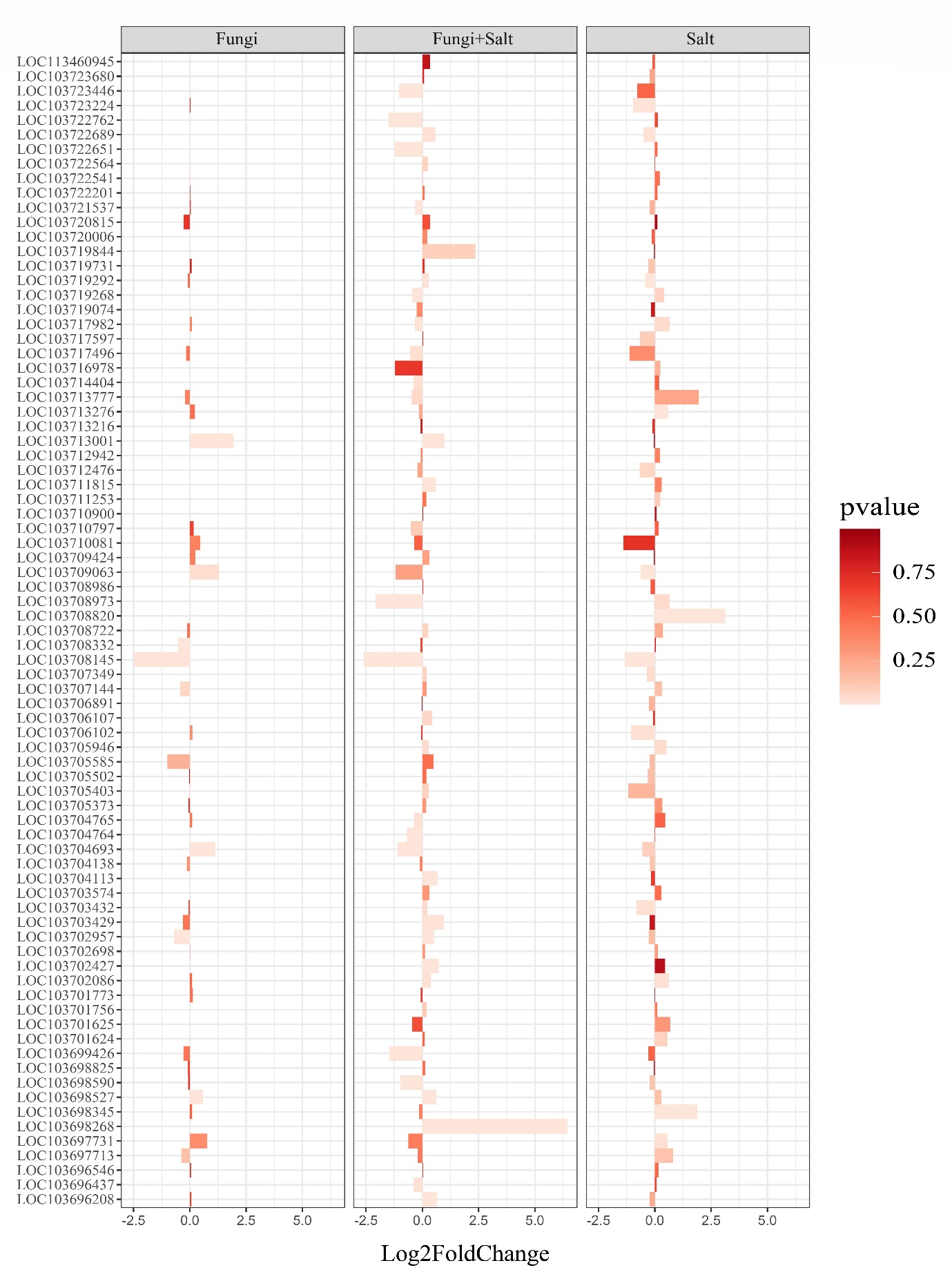


**Supplementary Figure 3:** Expression of DEGs associated with abscisic acid. The bar plot displays the DEGs involved in abscisic acid biosynthesis for different treatment conditions. The coordinates of the figure represent the statistics of genes expression.


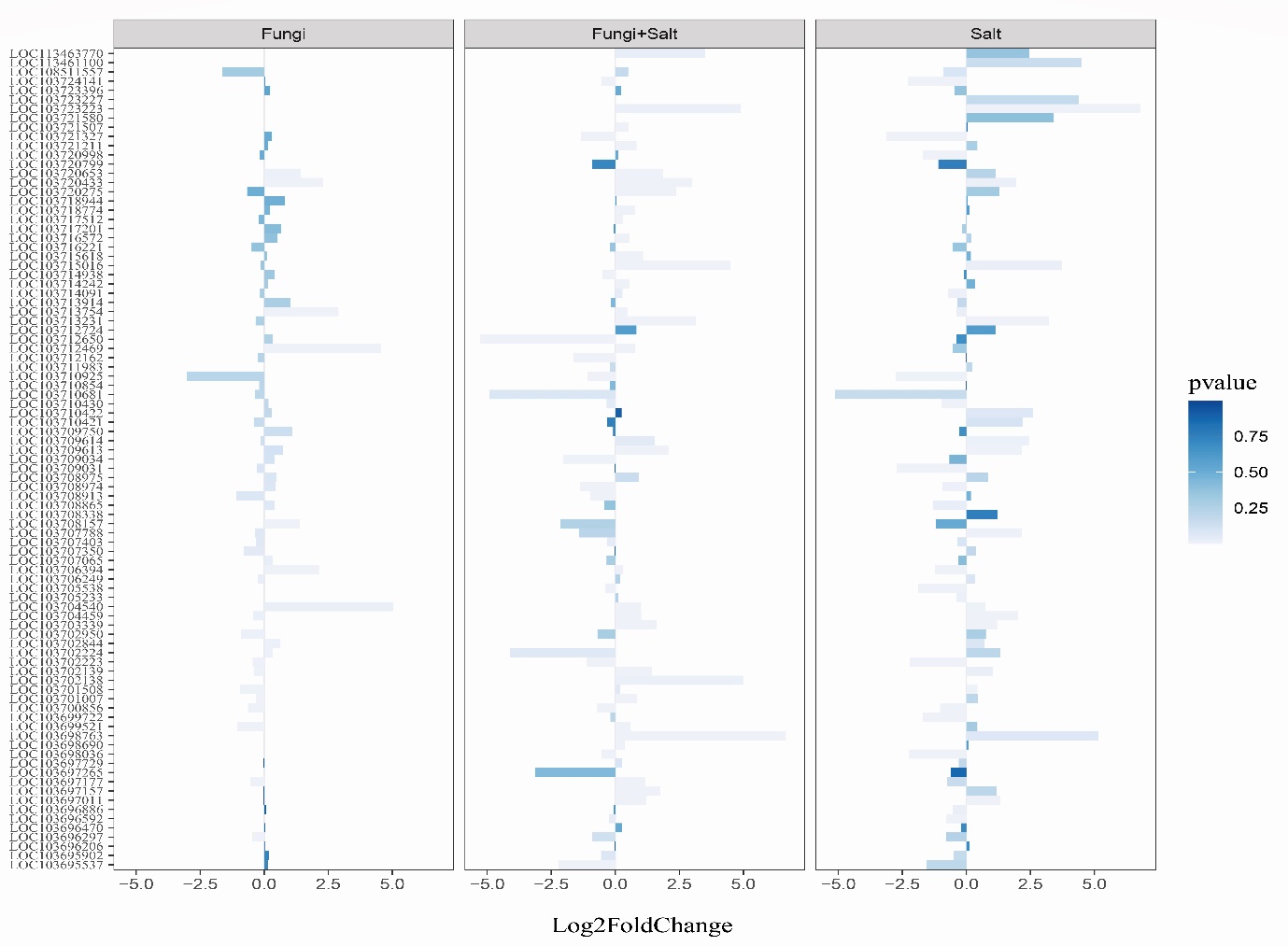


**Supplementary Figure 4:** Expression of DEGs associated with WRKYs transcription factors. The bar plot displays the DEGs involved in WRKYs transcription factors related processes for different treatment conditions. The coordinates of the figure represent the statistics of genes expression.


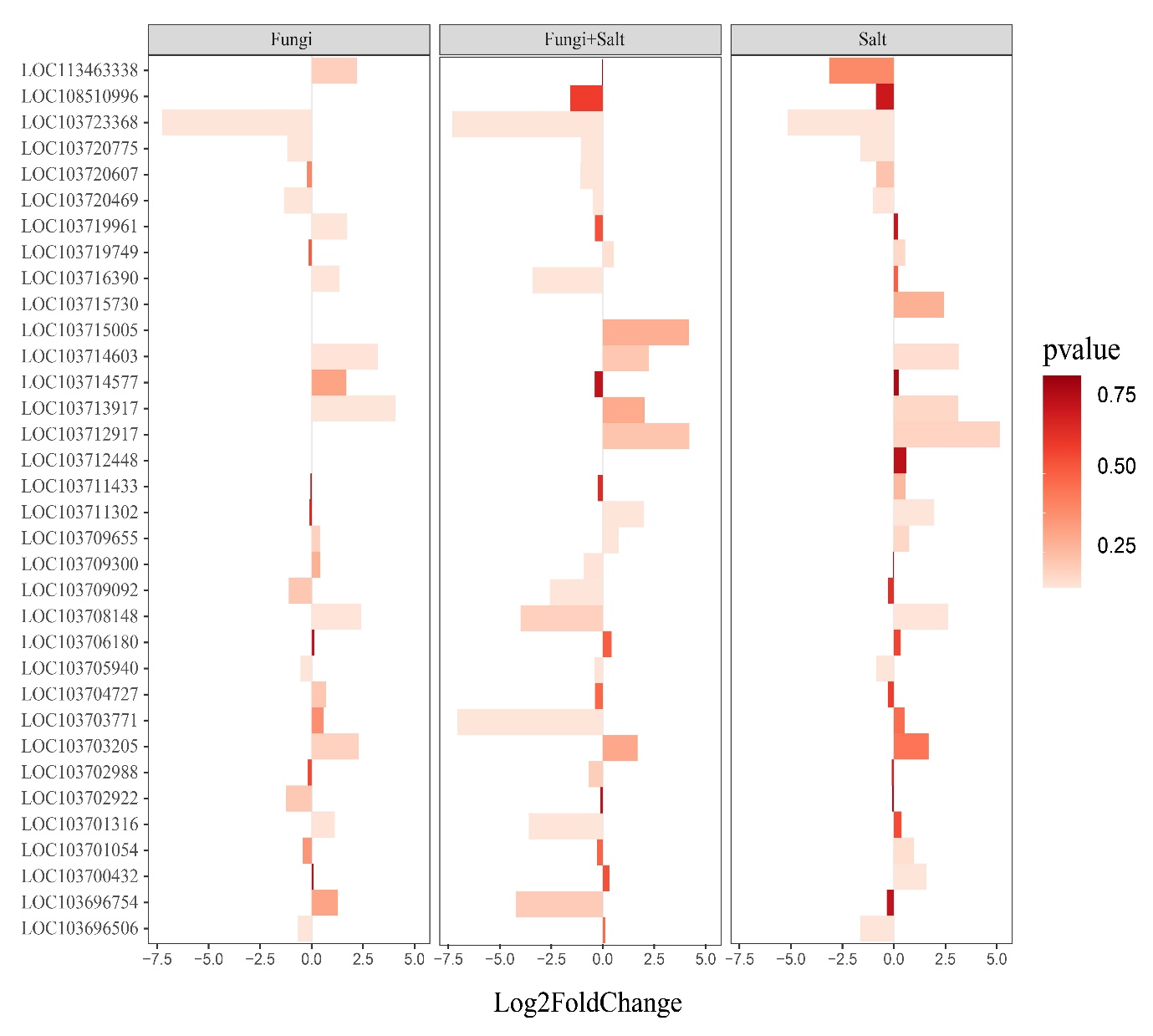


**Supplementary Figure 5:** Expression of DEGs associated with MYBs transcription factor. The bar plot displays the DEGs involved in MYBs transcription factor related processes for different treatment conditions. The coordinates of the figure represent the statistics of genes expression.


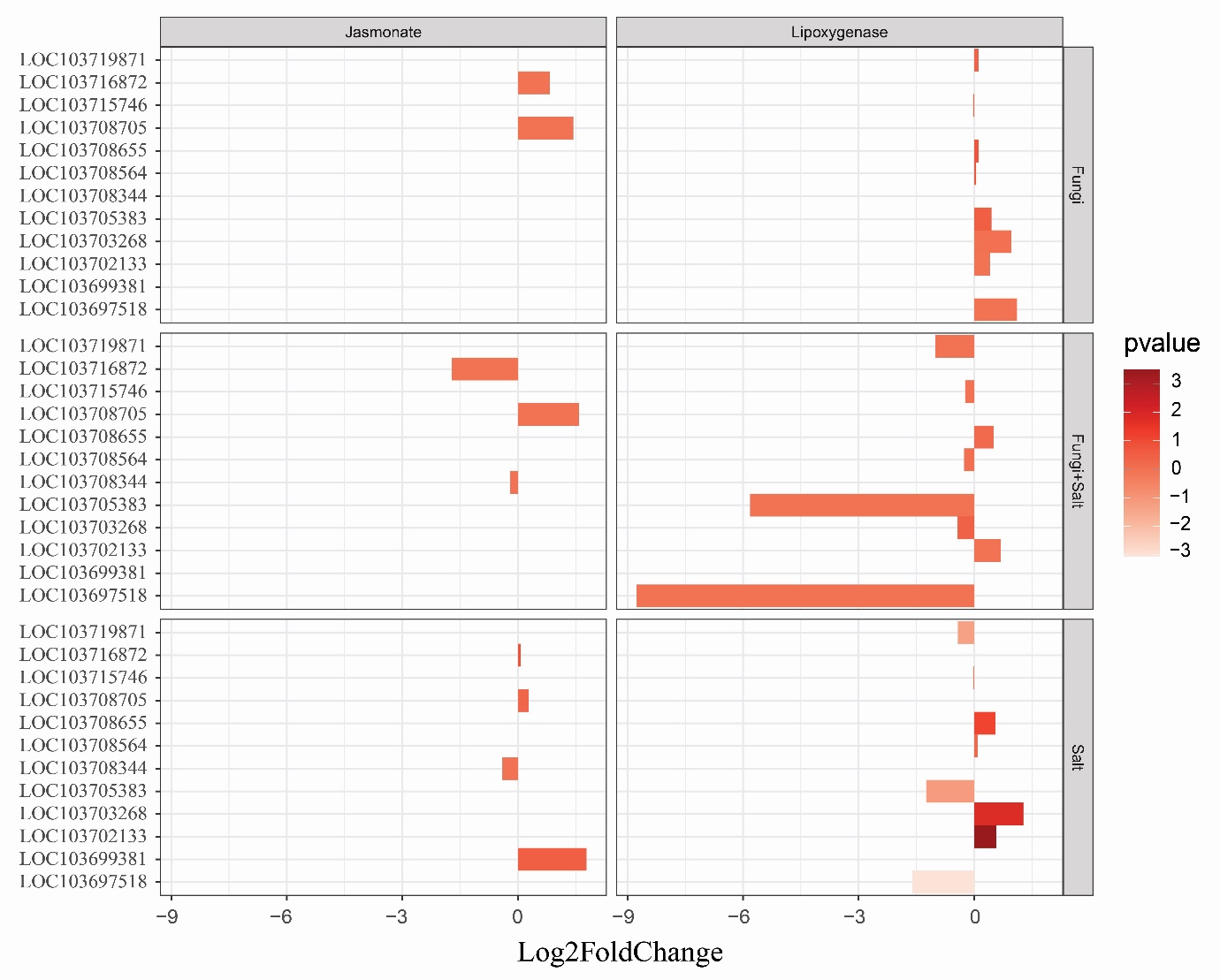


**Supplementary Figure 6:** Expression of DEGs associated with Jasmonate and Lipoxygenases. The bar plot displays the DEGs involved in Jasmonate and Lipoxygenases biosynthesis for different treatment conditions. The coordinates of the figure represent the statistics of genes expression.


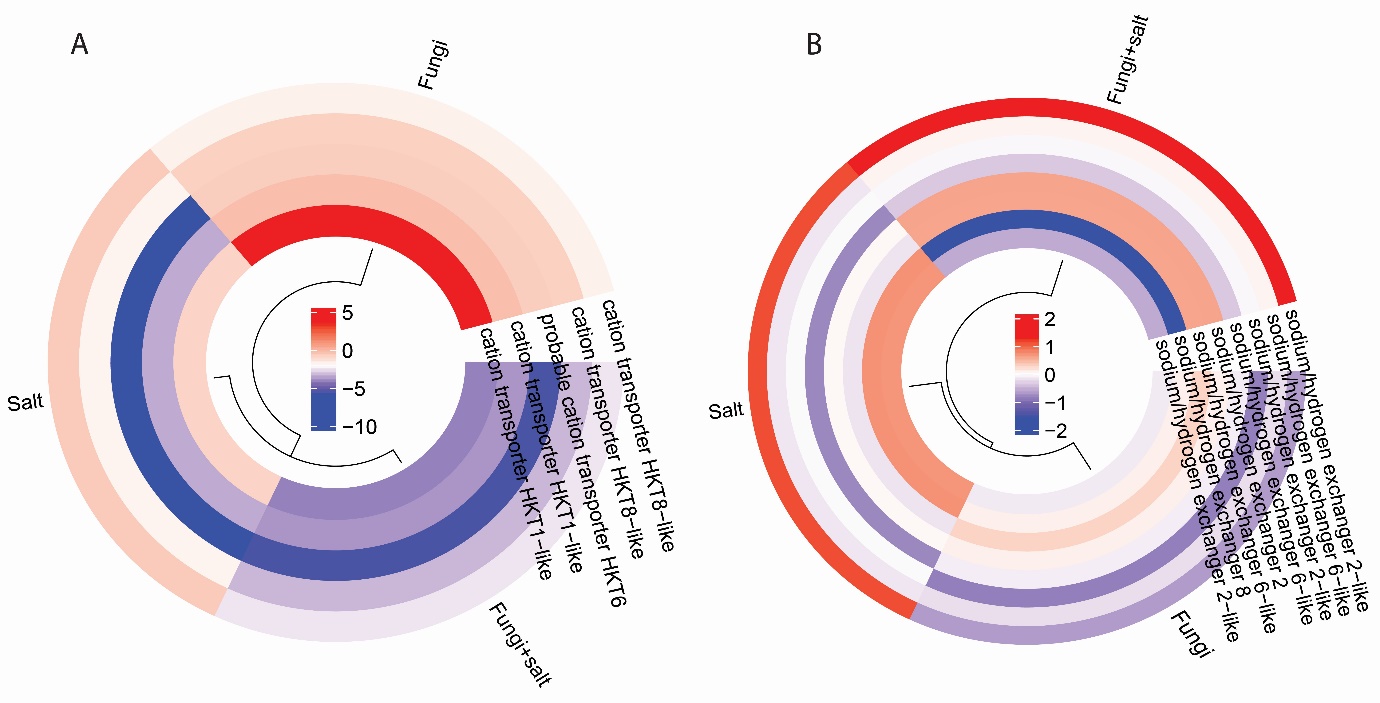


**Supplementary Figure 7:** Circular heatmap showing the expression of DEGs associated with transporter genes. (A) HKT and (B) Sodium/Hydrogen exchanger genes in different treatment conditions.


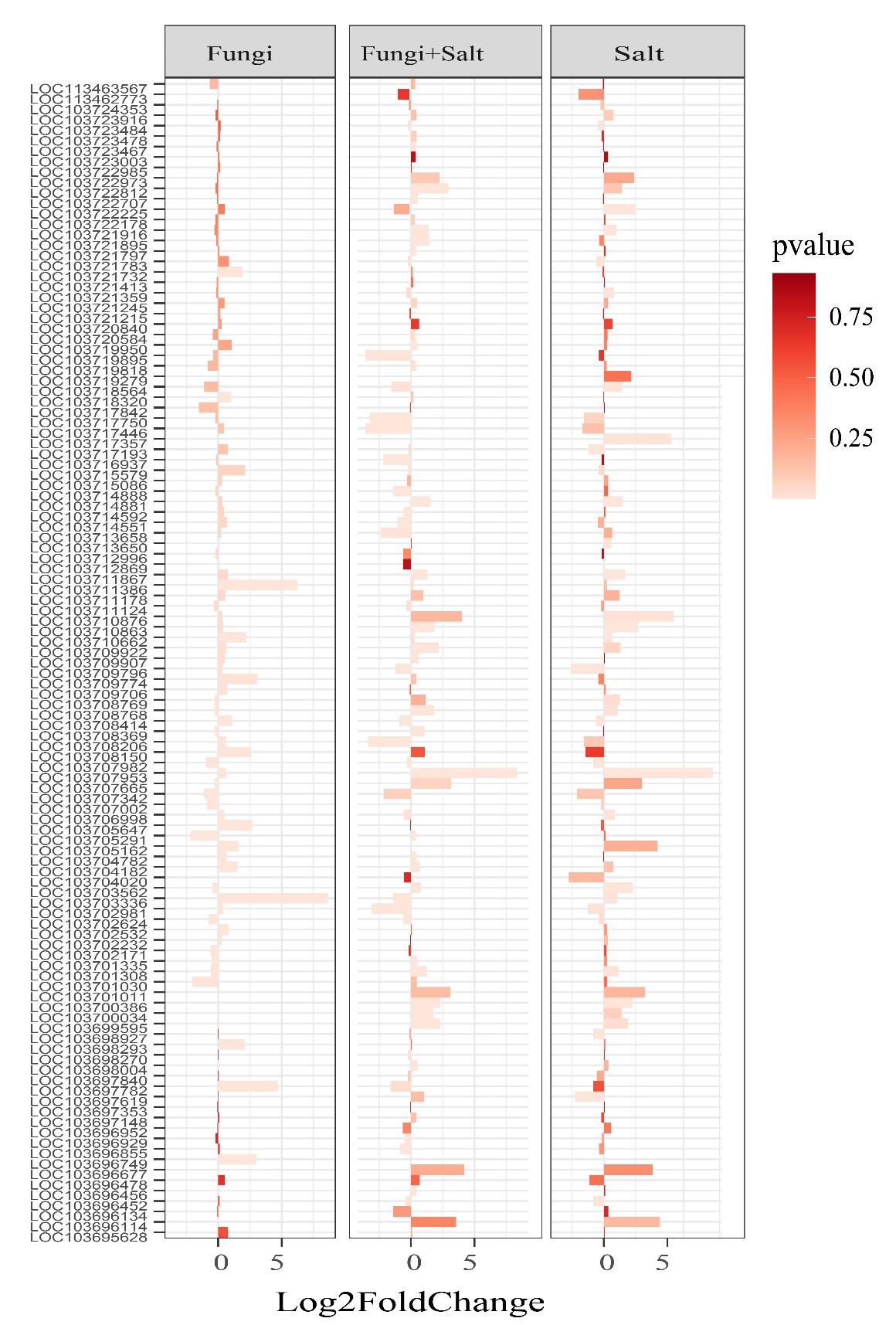


**Supplementary Figure 8:** Expression of DEGs associated with ABC transporters. The bar plot displays the DEGs involved in ABC transporters related processes for different treatment conditions. The coordinates of the figure represent the statistics of genes expression.


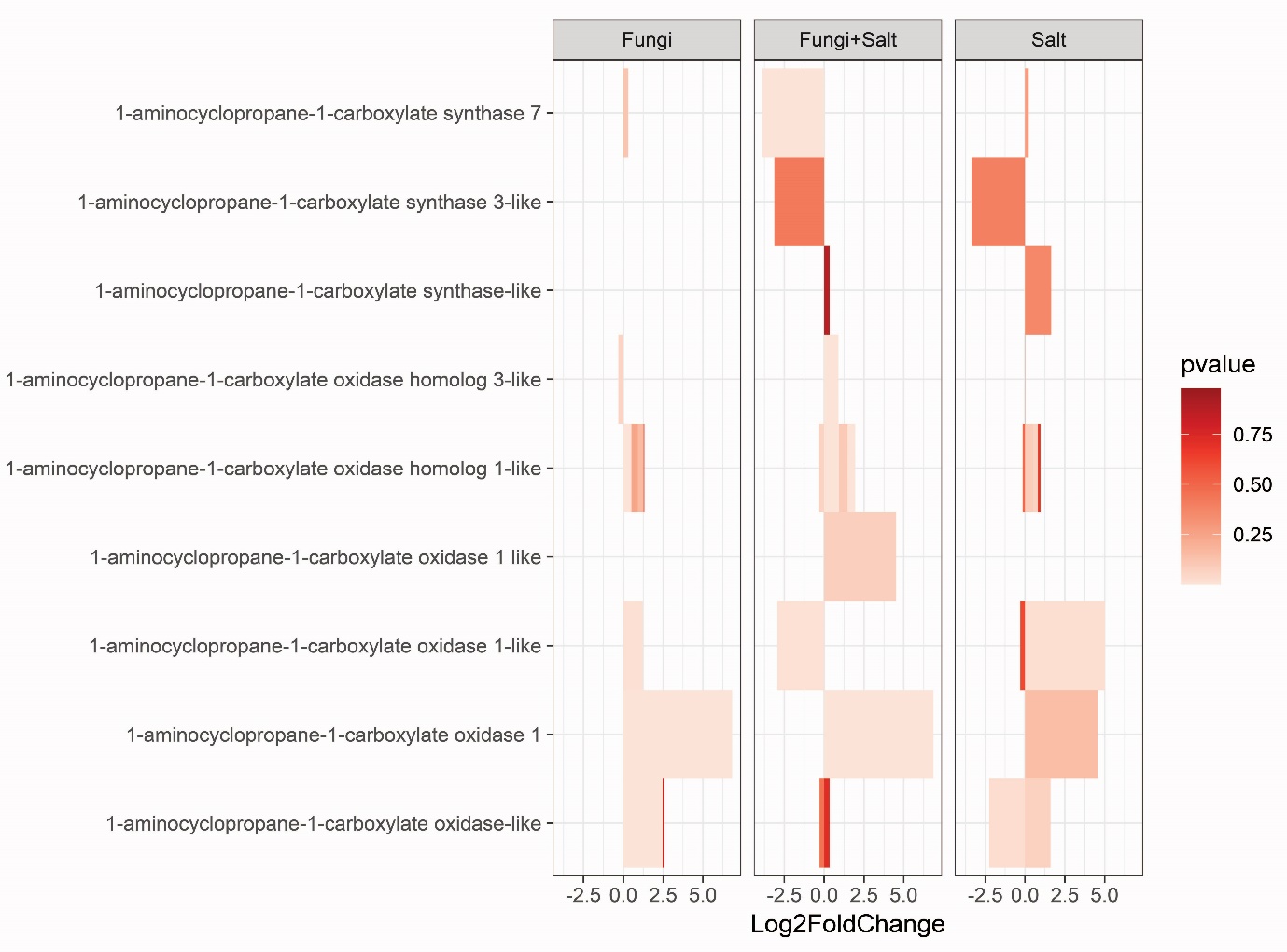


**Supplementary Figure 9:** Expression of DEGs associated with ethylene. The bar plot displays the DEGs involved in ethylene biosynthesis for different treatment conditions. The coordinates of the figure represent the statistics of genes expression.

## Supplementary tables

Table S1: Sequencing reads across the different data palm seedling treatment samples

| Sample | Raw reads | Clean reads | Mapped reads |
| --- | --- | --- | --- |
| C1 | 67295188 | 62040451 (92.19%) | 5254737 |
| C2 | 54919324 | 50967303 (92.8%) | 3952021 |
| C3 | 68355270 | 63315844 (92.63%) | 5039426 |
| F1 | 41237610 | 36898213 (89.48%) | 4339397 |
| F2 | 39977852 | 36520957 (91.35%) | 3456895 |
| F3 | 40558832 | 37164832 (91.63%) | 3394000 |
| FS1 | 46855944 | 43490872 (92.82%) | 3365072 |
| FS2 | 46414820 | 43139385 (92.94%) | 3275435 |
| FS3 | 46194932 | 42813268 (92.68%) | 3381664 |
| S1 | 45819204 | 40568400 (88.54%) | 5250804 |
| S2 | 46528840 | 41693599 (89.61%) | 4835241 |
| S3 | 58877964 | 54004200 (91.72%) | 4873764 |

Table S2: Distribution of DEGs across the different data palm seedling treatment samples

| Treatment | Total DEGs | Upregulated DEGs | Downregulated DEGs |
| --- | --- | --- | --- |
| Salt | 25902 | 2523 | 2323 |
| Fungi | 23351 | 2031 | 959 |
| Fungi+Salt | 25679 | 1936 | 3546 |

Table 3: Gibberellin associated DEGs in different date palm seedling treatment groups

| **Differentially expressed genes related to gibberellin in the Fungus treated group** | | | | | | | | |
| --- | --- | --- | --- | --- | --- | --- | --- | --- |
| **Gene_Symbol** | **log2FoldChange** | **lfcSE** | **stat** | **pvalue** | **Type** | **Protein_ID** | **Product** | |
| LOC103703224 | 2.522348 | 1.128315 | 2.2355 | 0.025385 | protein_coding | XP_008784232.1 | gibberellin-regulated protein 6 | |
| LOC103704193 | -0.83898 | 1.18053 | -0.71068 | 0.477285 | protein_coding | XP_008785607.2; XP_026659459.1 | gibberellin 3-beta-dioxygenase 1-like | |
| LOC103711117 | 0.397555 | 0.203622 | 1.952416 | 0.050889 | protein_coding | XP_008795357.3 | gibberellin 2-beta-dioxygenase 6 | |
| LOC103717366 | 0.180172 | 0.405966 | 0.44381 | 0.65718 | protein_coding | XP_017700854.2 | gibberellin 2-beta-dioxygenase 6-like | |
| LOC103718258 | 0.718655 | 0.390954 | 1.83821 | 0.066031 | protein_coding | XP_008805212.1 | gibberellin 2-beta-dioxygenase 1 | |
| LOC103701020 | 0.174111 | 0.817555 | 0.212965 | 0.831354 | protein_coding | XP_008781172.1 | gibberellin 2-beta-dioxygenase 2-like | |
| LOC103701293 | -0.165 | 1.215366 | -0.13576 | 0.892009 | protein_coding | XP_008781527.1 | gibberellin-regulated protein 9 | |
| LOC103705541 | 1.861488 | 1.07868 | 1.725709 | 0.0844 | protein_coding | XP_026659861.1; XP_008787509.1; XP_008787515.1 | gibberellin 20-oxidase-like protein | |
| LOC103709077 | 2.111865 | 1.334663 | 1.582321 | 0.113576 | protein_coding | XP_008792485.1 | gibberellin-regulated protein 5-like | |
| LOC103709450 | -6.61982 | 1.505298 | -4.39768 | 1.09E-05 | protein_coding | XP_008792996.1 | gibberellin 2-beta-dioxygenase 3-like | |
| LOC103710942 | -0.36709 | 0.141353 | -2.59701 | 0.009404 | protein_coding | XP_008795099.1 | gibberellin receptor GID1C-like | |
| LOC103712632 | -0.25967 | 0.123154 | -2.10852 | 0.034986 | protein_coding | XP_008797426.1; XP_008797425.1 | chitin-inducible gibberellin-responsive protein 1-like | |
| LOC103714936 | -0.00868 | 0.091762 | -0.09454 | 0.924681 | protein_coding | XP_008800637.2 | chitin-inducible gibberellin-responsive protein 2-like | |
| LOC103715949 | 1.712048 | 1.120703 | 1.527656 | 0.126598 | protein_coding | XP_008801975.1 | gibberellin 2-beta-dioxygenase 1-like | |
| LOC103719746 | 5.212673 | 0.946147 | 5.509367 | 3.6E-08 | protein_coding | XP_008807353.1 | gibberellin-regulated protein 6-like | |
| LOC103721037 | 0.260174 | 0.145719 | 1.785457 | 0.074187 | protein_coding | XP_017701655.1 | gibberellin receptor GID1C-like | |
| LOC103722469 | -3.53806 | 0.603756 | -5.86007 | 4.63E-09 | protein_coding | XP_008811259.2 | gibberellin 2-beta-dioxygenase 3-like | |
| LOC103696316 | -0.34789 | 0.188767 | -1.84298 | 0.065331 | protein_coding | XP_008776123.1 | chitin-inducible gibberellin-responsive protein 1-like | |
| LOC103697092 | -0.40969 | 0.673995 | -0.60786 | 0.543283 | protein_coding | XP_008777097.1 | gibberellin 20 oxidase 1-D-like | |
| LOC103700279 | -0.20381 | 1.313322 | -0.15519 | 0.876674 | protein_coding | XP_008780452.1 | gibberellin 20 oxidase 2-like | |
| **Differentially expressed genes related to gibberellin in the Salt treated group** | | | | | | | | |
| **Gene_Symbol** | **log2FoldChange** | **lfcSE** | **Stat** | **pvalue** | **Type** | **Protein_ID** | | **Product** |
| LOC103703224 | 0.386567 | 1.996543 | 0.193618 | 0.846475 | protein_coding | XP_008784232.1 | | gibberellin-regulated protein 6 |
| LOC103704193 | 3.562211 | 0.678483 | 5.25026 | 1.52E-07 | protein_coding | XP_008785607.2; XP_026659459.1 | | gibberellin 3-beta-dioxygenase 1-like |
| LOC103711117 | 0.423011 | 0.241494 | 1.751646 | 0.079835 | protein_coding | XP_008795357.3 | | gibberellin 2-beta-dioxygenase 6 |
| LOC103717366 | -0.02499 | 0.358593 | -0.06968 | 0.944446 | protein_coding | XP_017700854.2 | | gibberellin 2-beta-dioxygenase 6-like |
| LOC103718258 | 0.580838 | 0.597508 | 0.9721 | 0.331001 | protein_coding | XP_008805212.1 | | gibberellin 2-beta-dioxygenase 1 |
| LOC103701020 | -0.07555 | 1.214203 | -0.06222 | 0.950388 | protein_coding | XP_008781172.1 | | gibberellin 2-beta-dioxygenase 2-like |
| LOC103701293 | -0.17748 | 1.753521 | -0.10121 | 0.919382 | protein_coding | XP_008781527.1 | | gibberellin-regulated protein 9 |
| LOC103702904 | 3.209418 | 1.87315 | 1.71338 | 0.086643 | protein_coding | XP_017697226.1 | | gibberellin 20 oxidase 1-D |
| LOC103705541 | -0.02076 | 1.954887 | -0.01062 | 0.991526 | protein_coding | XP_026659861.1; XP_008787509.1; XP_008787515.1 | | gibberellin 20-oxidase-like protein |
| LOC103709077 | -0.29064 | 3.253929 | -0.08932 | 0.928828 | protein_coding | XP_008792485.1 | | gibberellin-regulated protein 5-like |
| LOC103709450 | 0.796828 | 0.941801 | 0.846068 | 0.397515 | protein_coding | XP_008792996.1 | | gibberellin 2-beta-dioxygenase 3-like |
| LOC103709678 | 1.092593 | 1.363636 | 0.801235 | 0.422996 | protein_coding | XP_008793360.1 | | gibberellin 2-beta-dioxygenase 2-like |
| LOC103710942 | 1.047128 | 0.36382 | 2.878152 | 0.004 | protein_coding | XP_008795099.1 | | gibberellin receptor GID1C-like |
| LOC103712632 | -0.51695 | 0.198155 | -2.60881 | 0.009086 | protein_coding | XP_008797426.1; XP_008797425.1 | | chitin-inducible gibberellin-responsive protein 1-like |
| LOC103714936 | -0.42136 | 0.1359 | -3.10054 | 0.001932 | protein_coding | XP_008800637.2 | | chitin-inducible gibberellin-responsive protein 2-like |
| LOC103715949 | 1.404394 | 1.695533 | 0.828291 | 0.407506 | protein_coding | XP_008801975.1 | | gibberellin 2-beta-dioxygenase 1-like |
| LOC103717451 | 2.427512 | 1.909935 | 1.270992 | 0.203732 | protein_coding | XP_008804067.1 | | gibberellin 3-beta-dioxygenase 1-like |
| LOC103719746 | 0.809041 | 3.543549 | 0.228314 | 0.819402 | protein_coding | XP_008807353.1 | | gibberellin-regulated protein 6-like |
| LOC103721037 | 1.135917 | 0.275184 | 4.12784 | 3.66E-05 | protein_coding | XP_017701655.1 | | gibberellin receptor GID1C-like |
| LOC103721482 | -1.30815 | 2.56589 | -0.50982 | 0.610174 | protein_coding | XP_008809946.1 | | gibberellin 2-beta-dioxygenase 2 |
| LOC103722469 | -0.75674 | 0.443966 | -1.7045 | 0.088288 | protein_coding | XP_008811259.2 | | gibberellin 2-beta-dioxygenase 3-like |
| LOC103722719 | 4.169253 | 3.935831 | 1.059307 | 0.28946 | protein_coding | XP_008811603.2 | | gibberellin 3-beta-dioxygenase 1 |
| LOC103696316 | -0.98442 | 0.241265 | -4.08026 | 4.50E-05 | protein_coding | XP_008776123.1 | | chitin-inducible gibberellin-responsive protein 1-like |
| LOC103697092 | 1.591479 | 0.616592 | 2.581091 | 0.009849 | protein_coding | XP_008777097.1 | | gibberellin 20 oxidase 1-D-like |
| LOC103700279 | -3.04079 | 2.392146 | -1.27116 | 0.203673 | protein_coding | XP_008780452.1 | | gibberellin 20 oxidase 2-like |
| **Differentially expressed genes related to gibberellin in the Fungi+Salt treated group** | | | | | | | | |
| **Gene_Symbol** | **log2FoldChange** | **lfcSE** | **stat** | **pvalue** | **Type** | **Protein_ID** | **Product** | |
| LOC103722719 | 4.014358 | 2.858824 | 1.404199 | 0.16026 | protein_coding | XP_008811603.2 | gibberellin 3-beta-dioxygenase 1 | |
| LOC103722469 | -0.2023 | 0.302588 | -0.66858 | 0.503766 | protein_coding | XP_008811259.2 | gibberellin 2-beta-dioxygenase 3-like | |
| LOC103721482 | 2.971807 | 0.815891 | 3.642406 | 0.00027 | protein_coding | XP_008809946.1 | gibberellin 2-beta-dioxygenase 2 | |
| LOC103721037 | 0.804284 | 0.142148 | 5.658072 | 1.53E-08 | protein_coding | XP_017701655.1 | gibberellin receptor GID1C-like | |
| LOC103719746 | -0.70119 | 3.331984 | -0.21044 | 0.833324 | protein_coding | XP_008807353.1 | gibberellin-regulated protein 6-like | |
| LOC103718258 | -0.07441 | 0.438552 | -0.16968 | 0.865265 | protein_coding | XP_008805212.1 | gibberellin 2-beta-dioxygenase 1 | |
| LOC103717451 | 0.789315 | 1.917371 | 0.411665 | 0.680585 | protein_coding | XP_008804067.1 | gibberellin 3-beta-dioxygenase 1-like | |
| LOC103717366 | 0.116675 | 0.385344 | 0.30278 | 0.762057 | protein_coding | XP_017700854.2 | gibberellin 2-beta-dioxygenase 6-like | |
| LOC103716836 | 3.517469 | 3.211148 | 1.095393 | 0.273345 | protein_coding | XP_008803234.2 | gibberellin 2-beta-dioxygenase 8-like | |
| LOC103715949 | -1.66152 | 2.086977 | -0.79614 | 0.425953 | protein_coding | XP_008801975.1 | gibberellin 2-beta-dioxygenase 1-like | |
| LOC103714936 | -0.29827 | 0.079158 | -3.7681 | 0.000164 | protein_coding | XP_008800637.2 | chitin-inducible gibberellin-responsive protein 2-like | |
| LOC103712632 | -0.25256 | 0.104411 | -2.41892 | 0.015567 | protein_coding | XP_008797426.1; XP_008797425.1 | chitin-inducible gibberellin-responsive protein 1-like | |
| LOC103711117 | 0.929275 | 0.175986 | 5.280384 | 1.29E-07 | protein_coding | XP_008795357.3 | gibberellin 2-beta-dioxygenase 6 | |
| LOC103710942 | 0.362689 | 0.095959 | 3.779622 | 0.000157 | protein_coding | XP_008795099.1 | gibberellin receptor GID1C-like | |
| LOC103709678 | -4.60563 | 1.925588 | -2.39181 | 0.016766 | protein_coding | XP_008793360.1 | gibberellin 2-beta-dioxygenase 2-like | |
| LOC103709450 | 1.300655 | 0.576416 | 2.256452 | 0.024042 | protein_coding | XP_008792996.1 | gibberellin 2-beta-dioxygenase 3-like | |
| LOC103709077 | -3.78494 | 3.464518 | -1.09249 | 0.274619 | protein_coding | XP_008792485.1 | gibberellin-regulated protein 5-like | |
| LOC103705541 | -1.32698 | 2.04507 | -0.64887 | 0.516425 | protein_coding | XP_026659861.1; XP_008787509.1; XP_008787515.1 | gibberellin 20-oxidase-like protein | |
| LOC103704193 | 1.552544 | 0.626698 | 2.477339 | 0.013237 | protein_coding | XP_008785607.2; XP_026659459.1 | gibberellin 3-beta-dioxygenase 1-like | |
| LOC103703224 | -3.66137 | 2.886113 | -1.26862 | 0.204578 | protein_coding | XP_008784232.1 | gibberellin-regulated protein 6 | |
| LOC103701293 | -0.49708 | 1.240232 | -0.4008 | 0.688568 | protein_coding | XP_008781527.1 | gibberellin-regulated protein 9 | |
| LOC103701020 | -0.93907 | 0.721984 | -1.30067 | 0.19337 | protein_coding | XP_008781172.1 | gibberellin 2-beta-dioxygenase 2-like | |
| LOC103700279 | 0.417564 | 1.177865 | 0.354509 | 0.722958 | protein_coding | XP_008780452.1 | gibberellin 20 oxidase 2-like | |
| LOC103697092 | 0.491314 | 0.598098 | 0.821461 | 0.411384 | protein_coding | XP_008777097.1 | gibberellin 20 oxidase 1-D-like | |
| LOC103696316 | -0.02422 | 0.177831 | -0.13617 | 0.891684 | protein_coding | XP_008776123.1 | chitin-inducible gibberellin-responsive protein 1-like | |

Table S4: Auxin associated DEGs in the different date palm seedling treatment groups Abscisic acid associated differentially expressed genes in the Fungi treated group

| **Differentially expressed genes related to auxin in the Fungus control treated group** | | | | | | | | | | | | | | | | | | |
| --- | --- | --- | --- | --- | --- | --- | --- | --- | --- | --- | --- | --- | --- | --- | --- | --- | --- | --- |
| **Gene_Symbol** | | **log2FoldChange** | | | **lfcSE** | **Stat** | | | **Pvalue** | | | **Type** | | | **Protein_ID** | | | **Product** |
| LOC103703318 | | -3.70976 | | | 3.592314 | -1.03269 | | | 0.301747 | | | protein_coding | | | XP_008784357.2 | | | protein SMALL AUXIN UP-REGULATED RNA 10-like |
| LOC103703319 | | -0.92384 | | | 1.997992 | -0.46238 | | | 0.643807 | | | protein_coding | | | XP_026658850.1 | | | auxin-responsive protein SAUR76-like |
| LOC103703960 | | 0.470668 | | | 0.230486 | 2.042064 | | | 0.041145 | | | protein_coding | | | XP_008785276.1; XP_008785277.1; XP_026659200.1; XP_026659199.1; XP_008785274.1 | | | auxin response factor 18-like |
| LOC103704038 | | 0.485983 | | | 0.250183 | 1.942514 | | | 0.052075 | | | protein_coding | | | XP_026659225.1; XP_017697527.1; XP_008785388.1; XP_008785386.1; XP_008785387.1; XP_017697531.1; XP_008785385.1; XP_008785392.1 | | | auxin transporter-like protein 4 |
| LOC103704396 | | 1.210559 | | | 0.317958 | 3.807285 | | | 0.000141 | | | protein_coding | | | XP_008785886.1 | | | auxin response factor 17-like |
| LOC103704533 | | -0.1226 | | | 0.38068 | -0.32205 | | | 0.747414 | | | protein_coding | | | XP_008786091.1 | | | auxin-induced protein 22D-like |
| LOC103704796 | | 0.240018 | | | 0.132526 | 1.811093 | | | 0.070126 | | | protein_coding | | | XP_008786460.1; XP_026659536.1 | | | auxin response factor 7-like |
| LOC103704816 | | 0.378932 | | | 0.351275 | 1.078732 | | | 0.280707 | | | protein_coding | | | XP_008786490.1 | | | auxin-responsive protein IAA25-like |
| LOC103705416 | | 0.884534 | | | 0.682785 | 1.29548 | | | 0.195155 | | | protein_coding | | | XP_008787344.1 | | | auxin-responsive protein SAUR71-like |
| LOC103705980 | | -0.75293 | | | 0.826019 | -0.91152 | | | 0.362022 | | | protein_coding | | | XP_008788131.1; XP_017697988.1 | | | auxin-responsive protein IAA30-like |
| LOC103705981 | | -0.65023 | | | 0.925485 | -0.70258 | | | 0.482318 | | | protein_coding | | | XP_008788132.1 | | | auxin-induced protein 22D-like |
| LOC103706152 | | -0.75783 | | | 0.632452 | -1.19823 | | | 0.230826 | | | protein_coding | | | XP_008788387.1 | | | auxin transporter-like protein 2 |
| LOC103706185 | | 0.335311 | | | 0.250134 | 1.340524 | | | 0.180075 | | | protein_coding | | | XP_008788445.1; XP_017698037.1 | | | probable auxin efflux carrier component 1c |
| LOC103706233 | | 0.408122 | | | 0.390347 | 1.045537 | | | 0.295775 | | | protein_coding | | | XP_026660149.1 | | | probable auxin efflux carrier component 1b |
| LOC103706840 | | -0.16872 | | | 0.289105 | -0.58359 | | | 0.559498 | | | protein_coding | | | XP_008789309.1 | | | auxin-responsive protein IAA27-like |
| LOC103706989 | | 0.631004 | | | 0.337272 | 1.870906 | | | 0.061358 | | | protein_coding | | | XP_008789525.1 | | | auxin-responsive protein IAA10 |
| LOC103707813 | | 0.185024 | | | 0.192051 | 0.963414 | | | 0.33534 | | | protein_coding | | | XP_008790698.1; XP_008790697.1; XP_008790699.1; XP_008790695.1 | | | auxin response factor 2A-like |
| LOC103708057 | | -0.05663 | | | 0.262749 | -0.21553 | | | 0.829355 | | | protein_coding | | | XP_008791040.1; XP_008791039.1 | | | auxin response factor 23-like |
| LOC103708133 | | -1.92102 | | | 0.595201 | -3.22751 | | | 0.001249 | | | protein_coding | | | XP_008791133.1 | | | auxin-induced protein X15-like |
| LOC103708220 | | 0.605547 | | | 0.33664 | 1.798801 | | | 0.07205 | | | protein_coding | | | XP_008791264.1 | | | auxin-responsive protein SAUR50 |
| LOC103708418 | | 0.297558 | | | 0.174245 | 1.707703 | | | 0.087692 | | | protein_coding | | | XP_008791554.1 | | | auxin response factor 18 |
| LOC108511356 | | -2.2741 | | | 2.72382 | -0.8349 | | | 0.403777 | | | protein_coding | | | XP_026660945.1 | | | auxin-responsive protein SAUR50 |
| LOC103708499 | | -0.54772 | | | 0.465801 | -1.17588 | | | 0.239645 | | | protein_coding | | | XP_026660994.1 | | | auxin-responsive protein SAUR32-like |
| LOC108511370 | | 1.108308 | | | 1.790384 | 0.619034 | | | 0.535894 | | | protein_coding | | | XP_017698715.1 | | | auxin-responsive protein SAUR36-like |
| LOC103709322 | | 0.690908 | | | 0.230407 | 2.998643 | | | 0.002712 | | | protein_coding | | | XP_008792838.2 | | | auxin response factor 16-like |
| LOC103709490 | | 0.127664 | | | 0.190291 | 0.670887 | | | 0.502292 | | | protein_coding | | | XP_008793078.1; XP_008793077.1 | | | auxin-responsive protein IAA27-like |
| LOC103709827 | | -3.34868 | | | 3.86946 | -0.86541 | | | 0.386812 | | | protein_coding | | | XP_008793565.1 | | | auxin-responsive protein SAUR78 |
| LOC103709905 | | -0.01558 | | | 0.302724 | -0.05147 | | | 0.958949 | | | protein_coding | | | XP_008793654.1; XP_008793651.1 | | | auxin-binding protein 4 |
| LOC103710084 | | -0.14013 | | | 0.227238 | -0.61665 | | | 0.537468 | | | protein_coding | | | XP_008793917.1 | | | auxin response factor 17-like |
| LOC103710278 | | -1.99746 | | | 0.340426 | -5.86752 | | | 4.42E-09 | | | protein_coding | | | XP_026661678.1; XP_026661677.1 | | | auxin-responsive protein IAA7-like |
| LOC103710514 | | 0.276759 | | | 0.144191 | 1.91939 | | | 0.054935 | | | protein_coding | | | XP_008794474.1; XP_026661767.1 | | | auxin response factor 17 |
| LOC103711069 | | -0.18283 | | | 0.155286 | -1.17739 | | | 0.239041 | | | protein_coding | | | XP_008795277.1; XP_008795276.1 | | | auxin response factor 7-like |
| LOC103711175 | | -0.89511 | | | 1.132789 | -0.79018 | | | 0.42942 | | | protein_coding | | | XP_017699290.1; XP_008795449.1; XP_008795448.1 | | | auxin transporter-like protein 2 |
| LOC103711915 | | 4.797405 | | | 2.350303 | 2.041186 | | | 0.041232 | | | protein_coding | | | XP_008796457.1 | | | auxin-induced protein X10A |
| LOC103712176 | | 0.667755 | | | 0.323576 | 2.06367 | | | 0.039049 | | | protein_coding | | | XP_008796846.1 | | | auxin-responsive protein SAUR36-like |
| LOC103712188 | | 1.486942 | | | 0.641703 | 2.31718 | | | 0.020494 | | | protein_coding | | | XP_008796867.1 | | | auxin-responsive protein SAUR32-like |
| LOC103712757 | | 0.468707 | | | 0.205405 | 2.281868 | | | 0.022497 | | | protein_coding | | | XP_008797599.1 | | | auxin transport protein BIG |
| LOC103713126 | | 0.271336 | | | 0.140362 | 1.93312 | | | 0.053221 | | | protein_coding | | | XP_008798164.1; XP_008798163.1 | | | auxin response factor 19-like |
| LOC103713207 | | 0.390798 | | | 0.213915 | 1.826882 | | | 0.067718 | | | protein_coding | | | XP_008798267.1; XP_008798269.1; XP_008798266.1; XP_008798270.1 | | | auxin response factor 15-like |
| LOC103713388 | | -0.87097 | | | 0.459249 | -1.8965 | | | 0.057894 | | | protein_coding | | | XP_008798522.1 | | | auxin-responsive protein SAUR32-like |
| LOC103713744 | | 0.051624 | | | 1.248766 | 0.04134 | | | 0.967025 | | | protein_coding | | | XP_017699908.1 | | | auxin efflux carrier component 6 |
| LOC103714011 | | -0.42557 | | | 0.546979 | -0.77803 | | | 0.436551 | | | protein_coding | | | XP_008799332.1 | | | auxin response factor 12-like |
| LOC103714723 | | -1.02183 | | | 0.427545 | -2.38999 | | | 0.016849 | | | protein_coding | | | XP_026663329.1; XP_008800316.1; XP_008800317.1; XP_026663330.1 | | | auxin-responsive protein IAA4 |
| LOC103714909 | | 0.197205 | | | 0.290946 | 0.677808 | | | 0.497894 | | | protein_coding | | | XP_026663424.1 | | | auxin-responsive protein IAA10-like |
| LOC103715141 | | 2.495122 | | | 2.216893 | 1.125504 | | | 0.260375 | | | protein_coding | | | XP_008800898.1 | | | auxin efflux carrier component 4 |
| LOC103715531 | | 1.49563 | | | 1.95897 | 0.763478 | | | 0.445178 | | | protein_coding | | | XP_008801409.1 | | | auxin-responsive protein SAUR50 |
| LOC103715543 | | 0.222479 | | | 0.245363 | 0.906735 | | | 0.364547 | | | protein_coding | | | XP_008801434.1 | | | auxin response factor 19-like |
| LOC103715742 | | -0.05768 | | | 0.177528 | -0.32491 | | | 0.74525 | | | protein_coding | | | XP_008801701.1 | | | protein AUXIN RESPONSE 4 |
| LOC103716143 | | 4.999592 | | | 3.827682 | 1.306167 | | | 0.191496 | | | protein_coding | | | XP_008802247.1 | | | auxin efflux carrier component 5-like |
| LOC103716609 | | -1.57327 | | | 0.513319 | -3.0649 | | | 0.002177 | | | protein_coding | | | XP_008802896.1 | | | auxin-responsive protein IAA25-like |
| LOC103716961 | | -0.26672 | | | 0.850323 | -0.31367 | | | 0.753769 | | | protein_coding | | | XP_008803402.1 | | | auxin-responsive protein SAUR76-like |
| LOC103716962 | | -1.34303 | | | 1.03838 | -1.29339 | | | 0.195878 | | | protein_coding | | | XP_008803404.1 | | | protein SMALL AUXIN UP-REGULATED RNA 10-like |
| LOC103717415 | | 4.155001 | | | 2.561969 | 1.6218 | | | 0.104846 | | | protein_coding | | | XP_008804012.1 | | | auxin-responsive protein SAUR50-like |
| LOC103717467 | | -4.87742 | | | 0.154504 | -31.5681 | | | 1E-218 | | | protein_coding | | | XP_008804094.1 | | | auxin-responsive protein SAUR71-like |
| LOC103717738 | | 0.704836 | | | 0.337271 | 2.089821 | | | 0.036634 | | | protein_coding | | | XP_026664500.1; XP_008804453.1; XP_026664501.1 | | | auxin-responsive protein IAA10-like |
| LOC103717948 | | 0.527283 | | | 2.216682 | 0.237871 | | | 0.811981 | | | protein_coding | | | XP_008804756.1 | | | auxin-responsive protein IAA10-like |
| LOC103719667 | | -0.13401 | | | 0.291153 | -0.46026 | | | 0.645326 | | | protein_coding | | | XP_008807236.1; XP_008807235.1 | | | auxin response factor 11-like |
| LOC103720127 | | -1.87217 | | | 1.786414 | -1.048 | | | 0.294637 | | | protein_coding | | | XP_008807919.1; XP_008807918.1 | | | auxin-responsive protein IAA31 |
| LOC103720239 | | 0.412572 | | | 0.240744 | 1.713735 | | | 0.086577 | | | protein_coding | | | XP_008808072.1 | | | auxin response factor 15-like |
| LOC103720420 | | 3.415531 | | | 3.955649 | 0.863456 | | | 0.387887 | | | protein_coding | | | XP_008808332.1 | | | auxin-responsive protein IAA33-like |
| LOC103720508 | | 0.456805 | | | 0.518708 | 0.880659 | | | 0.378502 | | | protein_coding | | | XP_008808453.1 | | | auxin-induced protein 22D-like |
| LOC113463631 | | -4.07086 | | | 2.516507 | -1.61766 | | | 0.105735 | | | protein_coding | | | XP_026665714.1 | | | auxin-responsive protein SAUR36-like |
| LOC103721008 | | 0.344278 | | | 0.551144 | 0.624661 | | | 0.532194 | | | protein_coding | | | XP_008809247.1; XP_008809245.1; XP_008809246.1; XP_008809244.1; XP_008809249.1; XP_008809248.1 | | | auxin-responsive protein IAA9-like |
| LOC103721888 | | -0.42104 | | | 0.256999 | -1.6383 | | | 0.101358 | | | protein_coding | | | XP_026666141.1; XP_008810485.1; XP_017701849.1 | | | auxin-responsive protein IAA6-like |
| LOC103722035 | | 6.438252 | | | 1.099007 | 5.858247 | | | 4.68E-09 | | | protein_coding | | | XP_008810675.1 | | | auxin-responsive protein SAUR40 |
| LOC103722503 | | 4.6346 | | | 3.927839 | 1.179937 | | | 0.238025 | | | protein_coding | | | XP_008811310.1 | | | auxin-responsive protein IAA33 |
| LOC103722525 | | 2.446258 | | | 0.576046 | 4.246636 | | | 2.17E-05 | | | protein_coding | | | XP_008811333.1 | | | auxin-responsive protein SAUR71-like |
| LOC103722597 | | 4.875965 | | | 2.931697 | 1.663189 | | | 0.096275 | | | protein_coding | | | XP_008811425.1 | | | auxin-responsive protein SAUR50 |
| LOC103722704 | | 0.048825 | | | 2.504016 | 0.019499 | | | 0.984443 | | | protein_coding | | | XP_008811576.1 | | | auxin-responsive protein SAUR77 |
| LOC103722948 | | 0.15188 | | | 0.159344 | 0.953155 | | | 0.340512 | | | protein_coding | | | XP_008811924.1; XP_026655713.1 | | | auxin response factor 2A-like |
| LOC103723263 | | 4.632831 | | | 3.02744 | 1.53028 | | | 0.125947 | | | protein_coding | | | XP_017702173.1 | | | auxin-responsive protein SAUR36-like |
| LOC103723620 | | -0.65275 | | | 0.44509 | -1.46656 | | | 0.142496 | | | protein_coding | | | XP_008812806.1 | | | probable auxin efflux carrier component 1c |
| LOC103724300 | | -2.06042 | | | 1.058092 | -1.9473 | | | 0.051499 | | | protein_coding | | | XP_008813740.1; XP_008813739.1 | | | auxin response factor 24-like |
| LOC103695681 | | 0.714991 | | | 0.347742 | 2.056095 | | | 0.039773 | | | protein_coding | | | XP_008775284.1 | | | auxin efflux carrier component 3a-like |
| LOC103696418 | | -0.40018 | | | 0.15757 | -2.53969 | | | 0.011095 | | | protein_coding | | | XP_008776254.1 | | | auxin-repressed 12.5 kDa protein |
| LOC103696719 | | 0.39578 | | | 0.23044 | 1.7175 | | | 0.085888 | | | protein_coding | | | XP_017695991.1 | | | auxin response factor 17-like |
| LOC103697593 | | -0.20576 | | | 0.215401 | -0.95524 | | | 0.339456 | | | protein_coding | | | XP_008777704.1; XP_026657097.1 | | | auxin response factor 17-like |
| LOC103698930 | | -1.03582 | | | 1.401149 | -0.73927 | | | 0.459745 | | | protein_coding | | | XP_008779201.1 | | | auxin-responsive protein SAUR32-like |
| LOC103700433 | | 1.149744 | | | 2.945544 | 0.390333 | | | 0.69629 | | | protein_coding | | | XP_008780599.1 | | | auxin-responsive protein SAUR78-like |
| **Auxin associated differentially expressed genes in the Salt treated group** | | | | | | | | | | | | | | | | | | |
| **Gene_Symbol** | | **log2FoldChange** | | **lfcSE** | | | | **stat** | **pvalue** | | **Type** | | | **Protein_ID** | | | **Product** | |
| LOC103703960 | | 0.178698 | | 0.300246 | | | | 0.595171 | 0.551729 | | protein_coding | | | XP_008785276.1; XP_008785277.1; XP_026659200.1; XP_026659199.1; XP_008785274.1 | | | auxin response factor 18-like | |
| LOC103704038 | | 0.680558 | | 0.203977 | | | | 3.336445 | 0.000849 | | protein_coding | | | XP_026659225.1; XP_017697527.1; XP_008785388.1; XP_008785386.1; XP_008785387.1; XP_017697531.1; XP_008785385.1; XP_008785392.1 | | | auxin transporter-like protein 4 | |
| LOC103705980 | | 1.598766 | | 0.433041 | | | | 3.691951 | 0.000223 | | protein_coding | | | XP_008788131.1; XP_017697988.1 | | | auxin-responsive protein IAA30-like | |
| LOC103706233 | | 1.31645 | | 0.306089 | | | | 4.300876 | 1.7E-05 | | protein_coding | | | XP_026660149.1 | | | probable auxin efflux carrier component 1b | |
| LOC103706989 | | 0.634182 | | 0.342189 | | | | 1.85331 | 0.063838 | | protein_coding | | | XP_008789525.1 | | | auxin-responsive protein IAA10 | |
| LOC103708057 | | -0.12454 | | 0.128138 | | | | -0.97193 | 0.331084 | | protein_coding | | | XP_008791040.1; XP_008791039.1 | | | auxin response factor 23-like | |
| LOC103708133 | | 0.363929 | | 0.42522 | | | | 0.855862 | 0.392074 | | protein_coding | | | XP_008791133.1 | | | auxin-induced protein X15-like | |
| LOC103708220 | | -0.09466 | | 0.339222 | | | | -0.27905 | 0.780206 | | protein_coding | | | XP_008791264.1 | | | auxin-responsive protein SAUR50 | |
| LOC103708418 | | -0.24031 | | 0.138614 | | | | -1.7337 | 0.082972 | | protein_coding | | | XP_008791554.1 | | | auxin response factor 18 | |
| LOC103709322 | | -0.0305 | | 0.146282 | | | | -0.20852 | 0.834821 | | protein_coding | | | XP_008792838.2 | | | auxin response factor 16-like | |
| LOC103709490 | | 0.110666 | | 0.089348 | | | | 1.238588 | 0.215498 | | protein_coding | | | XP_008793078.1; XP_008793077.1 | | | auxin-responsive protein IAA27-like | |
| LOC103709905 | | 0.157954 | | 0.291357 | | | | 0.542133 | 0.587727 | | protein_coding | | | XP_008793654.1; XP_008793651.1 | | | auxin-binding protein 4 | |
| LOC103710278 | | -1.4648 | | 0.513234 | | | | -2.85406 | 0.004316 | | protein_coding | | | XP_026661678.1; XP_026661677.1 | | | auxin-responsive protein IAA7-like | |
| LOC103710514 | | 0.354655 | | 0.124967 | | | | 2.837999 | 0.00454 | | protein_coding | | | XP_008794474.1; XP_026661767.1 | | | auxin response factor 17 | |
| LOC103711069 | | -0.54299 | | 0.148669 | | | | -3.65234 | 0.00026 | | protein_coding | | | XP_008795277.1; XP_008795276.1 | | | auxin response factor 7-like | |
| LOC103711175 | | 0.589254 | | 0.530846 | | | | 1.110027 | 0.266987 | | protein_coding | | | XP_017699290.1; XP_008795449.1; XP_008795448.1 | | | auxin transporter-like protein 2 | |
| LOC103712176 | | 0.720362 | | 0.23162 | | | | 3.110104 | 0.00187 | | protein_coding | | | XP_008796846.1 | | | auxin-responsive protein SAUR36-like | |
| LOC103712757 | | -0.31904 | | 0.116771 | | | | -2.73219 | 0.006291 | | protein_coding | | | XP_008797599.1 | | | auxin transport protein BIG | |
| LOC103713744 | | 1.158428 | | 0.615599 | | | | 1.881791 | 0.059864 | | protein_coding | | | XP_017699908.1 | | | auxin efflux carrier component 6 | |
| LOC103714011 | | 0.047035 | | 0.453501 | | | | 0.103715 | 0.917395 | | protein_coding | | | XP_008799332.1 | | | auxin response factor 12-like | |
| LOC103714723 | | -0.22964 | | 0.273233 | | | | -0.84044 | 0.40066 | | protein_coding | | | XP_026663329.1; XP_008800316.1; XP_008800317.1; XP_026663330.1 | | | auxin-responsive protein IAA4 | |
| LOC103715141 | | 3.990367 | | 3.090971 | | | | 1.290975 | 0.196712 | | protein_coding | | | XP_008800898.1 | | | auxin efflux carrier component 4 | |
| LOC103715742 | | 0.135666 | | 0.164894 | | | | 0.822746 | 0.410652 | | protein_coding | | | XP_008801701.1 | | | protein AUXIN RESPONSE 4 | |
| LOC103716609 | | 0.183653 | | 0.265659 | | | | 0.691312 | 0.48937 | | protein_coding | | | XP_008802896.1 | | | auxin-responsive protein IAA25-like | |
| LOC103716961 | | -0.53693 | | 1.05632 | | | | -0.5083 | 0.611243 | | protein_coding | | | XP_008803402.1 | | | auxin-responsive protein SAUR76-like | |
| LOC103716962 | | -0.06101 | | 0.739407 | | | | -0.08251 | 0.93424 | | protein_coding | | | XP_008803404.1 | | | protein SMALL AUXIN UP-REGULATED RNA 10-like | |
| LOC103717738 | | 1.166875 | | 0.265379 | | | | 4.397015 | 1.1E-05 | | protein_coding | | | XP_026664500.1; XP_008804453.1; XP_026664501.1 | | | auxin-responsive protein IAA10-like | |
| LOC103719667 | | -0.06396 | | 0.209648 | | | | -0.3051 | 0.760292 | | protein_coding | | | XP_008807236.1; XP_008807235.1 | | | auxin response factor 11-like | |
| LOC103720127 | | -1.06807 | | 1.296263 | | | | -0.82396 | 0.40996 | | protein_coding | | | XP_008807919.1; XP_008807918.1 | | | auxin-responsive protein IAA31 | |
| LOC103720239 | | 0.24929 | | 0.182375 | | | | 1.366909 | 0.171654 | | protein_coding | | | XP_008808072.1 | | | auxin response factor 15-like | |
| LOC103720508 | | 1.029745 | | 0.36897 | | | | 2.790863 | 0.005257 | | protein_coding | | | XP_008808453.1 | | | auxin-induced protein 22D-like | |
| LOC103721008 | | 2.513049 | | 0.654065 | | | | 3.842202 | 0.000122 | | protein_coding | | | XP_008809247.1; XP_008809245.1; XP_008809246.1; XP_008809244.1; XP_008809249.1; XP_008809248.1 | | | auxin-responsive protein IAA9-like | |
| LOC103721888 | | -0.38442 | | 0.23386 | | | | -1.6438 | 0.100218 | | protein_coding | | | XP_026666141.1; XP_008810485.1; XP_017701849.1 | | | auxin-responsive protein IAA6-like | |
| LOC103722035 | | 3.744774 | | 1.330436 | | | | 2.814697 | 0.004882 | | protein_coding | | | XP_008810675.1 | | | auxin-responsive protein SAUR40 | |
| LOC103722525 | | -1.34576 | | 0.990341 | | | | -1.35888 | 0.174184 | | protein_coding | | | XP_008811333.1 | | | auxin-responsive protein SAUR71-like | |
| LOC103722948 | | -0.17216 | | 0.116565 | | | | -1.47698 | 0.139681 | | protein_coding | | | XP_008811924.1; XP_026655713.1 | | | auxin response factor 2A-like | |
| LOC103723620 | | -0.30809 | | 0.375147 | | | | -0.82126 | 0.411499 | | protein_coding | | | XP_008812806.1 | | | probable auxin efflux carrier component 1c | |
| LOC103724300 | | 0.982662 | | 0.62533 | | | | 1.571431 | 0.116083 | | protein_coding | | | XP_008813740.1; XP_008813739.1 | | | auxin response factor 24-like | |
| LOC103695681 | | 1.972275 | | 0.290823 | | | | 6.781704 | 1.19E-11 | | protein_coding | | | XP_008775284.1 | | | auxin efflux carrier component 3a-like | |
| LOC103696418 | | -0.12727 | | 0.092557 | | | | -1.375 | 0.169132 | | protein_coding | | | XP_008776254.1 | | | auxin-repressed 12.5 kDa protein | |
| LOC103697593 | | -0.06397 | | 0.101188 | | | | -0.63222 | 0.527244 | | protein_coding | | | XP_008777704.1; XP_026657097.1 | | | auxin response factor 17-like | |
| LOC103698930 | | -0.2925 | | 1.419659 | | | | -0.20604 | 0.836761 | | protein_coding | | | XP_008779201.1 | | | auxin-responsive protein SAUR32-like | |
| LOC103700433 | | 3.561417 | | 1.393168 | | | | 2.556345 | 0.010578 | | protein_coding | | | XP_008780599.1 | | | auxin-responsive protein SAUR78-like | |
| **Auxin associated differentially expressed genes in the Fungi+Salt treated group** | | | | | | | | | | | | | | | | | | |
| Gene_Symbol | log2FoldChange | | lfcSE | | | | stat | | | pvalue | | | Type | | | Protein_ID | | Product |
| LOC103703319 | -0.31294 | | 1.437265 | | | | -0.21774 | | | 0.827634 | | | protein_coding | | | XP_026658850.1 | | auxin-responsive protein SAUR76-like |
| LOC103703960 | 0.239465 | | 0.20429 | | | | 1.172182 | | | 0.241124 | | | protein_coding | | | XP_008785276.1; XP_008785277.1; XP_026659200.1; XP_026659199.1; XP_008785274.1 | | auxin response factor 18-like |
| LOC103704038 | -0.18112 | | 0.185984 | | | | -0.97386 | | | 0.330125 | | | protein_coding | | | XP_026659225.1; XP_017697527.1; XP_008785388.1; XP_008785386.1; XP_008785387.1; XP_017697531.1; XP_008785385.1; XP_008785392.1 | | auxin transporter-like protein 4 |
| LOC103704396 | 1.101354 | | 0.125656 | | | | 8.76485 | | | 1.87E-18 | | | protein_coding | | | XP_008785886.1 | | auxin response factor 17-like |
| LOC103704796 | 0.259378 | | 0.091785 | | | | 2.825937 | | | 0.004714 | | | protein_coding | | | XP_008786460.1; XP_026659536.1 | | auxin response factor 7-like |
| LOC103704816 | 0.269992 | | 0.281893 | | | | 0.95778 | | | 0.338174 | | | protein_coding | | | XP_008786490.1 | | auxin-responsive protein IAA25-like |
| LOC103705416 | -2.88477 | | 1.064318 | | | | -2.71044 | | | 0.006719 | | | protein_coding | | | XP_008787344.1 | | auxin-responsive protein SAUR71-like |
| LOC103705980 | -2.17176 | | 0.673484 | | | | -3.22466 | | | 0.001261 | | | protein_coding | | | XP_008788131.1; XP_017697988.1 | | auxin-responsive protein IAA30-like |
| LOC103705981 | -2.06474 | | 0.375556 | | | | -5.49781 | | | 3.85E-08 | | | protein_coding | | | XP_008788132.1 | | auxin-induced protein 22D-like |
| LOC103706152 | -0.61497 | | 0.542418 | | | | -1.13376 | | | 0.256897 | | | protein_coding | | | XP_008788387.1 | | auxin transporter-like protein 2 |
| LOC103706185 | -0.38937 | | 0.207431 | | | | -1.8771 | | | 0.060504 | | | protein_coding | | | XP_008788445.1; XP_017698037.1 | | probable auxin efflux carrier component 1c |
| LOC103706233 | -1.74565 | | 0.447104 | | | | -3.90435 | | | 9.45E-05 | | | protein_coding | | | XP_026660149.1 | | probable auxin efflux carrier component 1b |
| LOC103706840 | -0.78174 | | 0.170464 | | | | -4.58593 | | | 4.52E-06 | | | protein_coding | | | XP_008789309.1 | | auxin-responsive protein IAA27-like |
| LOC103706989 | -0.60606 | | 0.405751 | | | | -1.49368 | | | 0.13526 | | | protein_coding | | | XP_008789525.1 | | auxin-responsive protein IAA10 |
| LOC103708057 | 0.012989 | | 0.147893 | | | | 0.087826 | | | 0.930015 | | | protein_coding | | | XP_008791040.1; XP_008791039.1 | | auxin response factor 23-like |
| LOC103708133 | -1.33369 | | 0.530487 | | | | -2.51409 | | | 0.011934 | | | protein_coding | | | XP_008791133.1 | | auxin-induced protein X15-like |
| LOC103708220 | 1.143302 | | 0.238696 | | | | 4.789778 | | | 1.67E-06 | | | protein_coding | | | XP_008791264.1 | | auxin-responsive protein SAUR50 |
| LOC103708418 | -0.50609 | | 0.125318 | | | | -4.0384 | | | 5.38E-05 | | | protein_coding | | | XP_008791554.1 | | auxin response factor 18 |
| LOC108511370 | -0.70412 | | 2.833917 | | | | -0.24846 | | | 0.803776 | | | protein_coding | | | XP_017698715.1 | | auxin-responsive protein SAUR36-like |
| LOC103709322 | 0.396788 | | 0.125879 | | | | 3.152139 | | | 0.001621 | | | protein_coding | | | XP_008792838.2 | | auxin response factor 16-like |
| LOC103709827 | -0.69448 | | 2.482958 | | | | -0.2797 | | | 0.77971 | | | protein_coding | | | XP_008793565.1 | | auxin-responsive protein SAUR78 |
| LOC103709905 | 0.02874 | | 0.273959 | | | | 0.104905 | | | 0.916451 | | | protein_coding | | | XP_008793654.1; XP_008793651.1 | | auxin-binding protein 4 |
| LOC103710278 | 0.387147 | | 0.400216 | | | | 0.967345 | | | 0.333371 | | | protein_coding | | | XP_026661678.1; XP_026661677.1 | | auxin-responsive protein IAA7-like |
| LOC103710514 | 0.01683 | | 0.115798 | | | | 0.145338 | | | 0.884444 | | | protein_coding | | | XP_008794474.1; XP_026661767.1 | | auxin response factor 17 |
| LOC103712757 | 0.572271 | | 0.086871 | | | | 6.587579 | | | 4.47E-11 | | | protein_coding | | | XP_008797599.1 | | auxin transport protein BIG |
| LOC103713207 | -0.31241 | | 0.165326 | | | | -1.88967 | | | 0.058803 | | | protein_coding | | | XP_008798267.1; XP_008798269.1; XP_008798266.1; XP_008798270.1 | | auxin response factor 15-like |
| LOC103713388 | -0.02726 | | 0.357315 | | | | -0.07628 | | | 0.939195 | | | protein_coding | | | XP_008798522.1 | | auxin-responsive protein SAUR32-like |
| LOC103713744 | -3.37472 | | 1.353459 | | | | -2.49341 | | | 0.012652 | | | protein_coding | | | XP_017699908.1 | | auxin efflux carrier component 6 |
| LOC103714011 | -0.34938 | | 0.464883 | | | | -0.75154 | | | 0.452329 | | | protein_coding | | | XP_008799332.1 | | auxin response factor 12-like |
| LOC103714723 | -0.05093 | | 0.315376 | | | | -0.1615 | | | 0.871703 | | | protein_coding | | | XP_026663329.1; XP_008800316.1; XP_008800317.1; XP_026663330.1 | | auxin-responsive protein IAA4 |
| LOC103715141 | 2.188299 | | 1.470108 | | | | 1.48853 | | | 0.136611 | | | protein_coding | | | XP_008800898.1 | | auxin efflux carrier component 4 |
| LOC103715543 | -0.09724 | | 0.134914 | | | | -0.72077 | | | 0.471049 | | | protein_coding | | | XP_008801434.1 | | auxin response factor 19-like |
| LOC103715742 | -0.11783 | | 0.159774 | | | | -0.73745 | | | 0.460847 | | | protein_coding | | | XP_008801701.1 | | protein AUXIN RESPONSE 4 |
| LOC103716962 | -2.66015 | | 1.090994 | | | | -2.43828 | | | 0.014757 | | | protein_coding | | | XP_008803404.1 | | protein SMALL AUXIN UP-REGULATED RNA 10-like |
| LOC103717415 | 4.362794 | | 2.241131 | | | | 1.946692 | | | 0.051572 | | | protein_coding | | | XP_008804012.1 | | auxin-responsive protein SAUR50-like |
| LOC103717738 | 0.531976 | | 0.262874 | | | | 2.023693 | | | 0.043002 | | | protein_coding | | | XP_026664500.1; XP_008804453.1; XP_026664501.1 | | auxin-responsive protein IAA10-like |
| LOC103719667 | 0.091769 | | 0.16868 | | | | 0.544044 | | | 0.586411 | | | protein_coding | | | XP_008807236.1; XP_008807235.1 | | auxin response factor 11-like |
| LOC103720127 | -1.84268 | | 1.505361 | | | | -1.22408 | | | 0.220923 | | | protein_coding | | | XP_008807919.1; XP_008807918.1 | | auxin-responsive protein IAA31 |
| LOC103721008 | -0.40521 | | 0.496292 | | | | -0.81648 | | | 0.414228 | | | protein_coding | | | XP_008809247.1; XP_008809245.1; XP_008809246.1; XP_008809244.1; XP_008809249.1; XP_008809248.1 | | auxin-responsive protein IAA9-like |
| LOC103721888 | -1.03056 | | 0.188507 | | | | -5.46696 | | | 4.58E-08 | | | protein_coding | | | XP_026666141.1; XP_008810485.1; XP_017701849.1 | | auxin-responsive protein IAA6-like |
| LOC103722035 | 4.976894 | | 1.063434 | | | | 4.68002 | | | 2.87E-06 | | | protein_coding | | | XP_008810675.1 | | auxin-responsive protein SAUR40 |
| LOC103722503 | 4.508512 | | 3.17864 | | | | 1.418378 | | | 0.15608 | | | protein_coding | | | XP_008811310.1 | | auxin-responsive protein IAA33 |
| LOC103722704 | 2.08012 | | 1.345624 | | | | 1.545841 | | | 0.122143 | | | protein_coding | | | XP_008811576.1 | | auxin-responsive protein SAUR77 |
| LOC103722948 | 0.05906 | | 0.101208 | | | | 0.58355 | | | 0.559523 | | | protein_coding | | | XP_008811924.1; XP_026655713.1 | | auxin response factor 2A-like |
| LOC103724300 | 1.22968 | | 0.501253 | | | | 2.453209 | | | 0.014159 | | | protein_coding | | | XP_008813740.1; XP_008813739.1 | | auxin response factor 24-like |
| LOC103695681 | 0.260722 | | 0.330288 | | | | 0.789376 | | | 0.429893 | | | protein_coding | | | XP_008775284.1 | | auxin efflux carrier component 3a-like |
| LOC103696418 | 0.212175 | | 0.07445 | | | | 2.849901 | | | 0.004373 | | | protein_coding | | | XP_008776254.1 | | auxin-repressed 12.5 kDa protein |
| LOC103700433 | -0.2525 | | 2.982425 | | | | -0.08466 | | | 0.932529 | | | protein_coding | | | XP_008780599.1 | | auxin-responsive protein SAUR78-like |

Table 5: Abscisic acid involved differentially expressed genes in the different date palm seedling treatment groups

| **Abscisic acid associated differentially expressed genes in the Salt treated group** | | | | | | | | | | | | | | | | | | |
| --- | --- | --- | --- | --- | --- | --- | --- | --- | --- | --- | --- | --- | --- | --- | --- | --- | --- | --- |
| **Gene_Symbol** | **log2FoldChange** | | | **lfcSE** | | | **stat** | | | **pvalue** | | **Type** | | **Protein_ID** | | | | **product** |
| LOC103707144 | 0.322834 | | | 0.220279 | | | 1.46557 | | | 0.142766 | | protein_coding | | XP_008789785.1 | | | abscisic acid receptor PYL3-like | |
| LOC103702957 | -0.27173 | | | 0.194928 | | | -1.39401 | | | 0.163314 | | protein_coding | | XP_008783837.1 | | abscisic acid receptor PYL8 | | |
| LOC103704693 | -0.55401 | | | 0.321693 | | | -1.72216 | | | 0.08504 | | protein_coding | | XP_008786319.1 | | abscisic acid receptor PYR1 | | |
| LOC103705585 | -0.22872 | | | 0.163812 | | | -1.39623 | | | 0.162645 | | protein_coding | | XP_026659822.1; XP_026659821.1 | | ABSCISIC ACID-INSENSITIVE 5-like protein 2 | | |
| LOC103706891 | -0.25828 | | | 0.198696 | | | -1.29985 | | | 0.193652 | | protein_coding | | XP_008789382.1 | | abscisic acid receptor PYL12-like | | |
| LOC103708145 | -1.33173 | | | 0.411787 | | | -3.23402 | | | 0.001221 | | protein_coding | | XP_008791150.1 | | abscisic acid and environmental stress-inducible protein TAS14 | | |
| LOC103708332 | 0.042039 | | | 0.19738 | | | 0.212983 | | | 0.83134 | | protein_coding | | XP_008791430.1 | | abscisic acid receptor PYL8-like | | |
| LOC103709063 | -0.61849 | | | 0.170977 | | | -3.61742 | | | 0.000298 | | protein_coding | | XP_008792462.1 | | ABSCISIC ACID-INSENSITIVE 5-like protein 2 | | |
| LOC103711815 | 0.30438 | | | 0.354908 | | | 0.857631 | | | 0.391096 | | protein_coding | | XP_008796322.2 | | abscisic acid receptor PYR1-like | | |
| LOC103713001 | -0.03687 | | | 0.223256 | | | -0.16515 | | | 0.868823 | | protein_coding | | XP_008797969.1 | | abscisic acid 8'-hydroxylase 1 | | |
| LOC103714404 | 0.19027 | | | 0.296681 | | | 0.641329 | | | 0.521309 | | protein_coding | | XP_008799865.1 | | abscisic acid receptor PYL4 | | |
| LOC103716978 | 0.243046 | | | 0.187878 | | | 1.293633 | | | 0.195792 | | protein_coding | | XP_008803422.3 | | ABSCISIC ACID-INSENSITIVE 5-like protein 1 | | |
| LOC103717496 | -1.1286 | | | 1.22681 | | | -0.91995 | | | 0.357599 | | protein_coding | | XP_008804142.2 | | abscisic acid receptor PYL4-like | | |
| LOC103719731 | -0.28348 | | | 0.184576 | | | -1.53587 | | | 0.124571 | | protein_coding | | XP_008807330.1 | | ABSCISIC ACID-INSENSITIVE 5-like protein 2 | | |
| LOC103719844 | -0.03392 | | | 0.283512 | | | -0.11964 | | | 0.90477 | | protein_coding | | XP_008807492.1 | | abscisic acid and environmental stress-inducible protein-like | | |
| LOC103696437 | 0.074654 | | | 0.172338 | | | 0.43318 | | | 0.664884 | | protein_coding | | XP_008776287.1 | | abscisic acid receptor PYL4-like | | |
| LOC103698527 | 0.293816 | | | 0.203087 | | | 1.446748 | | | 0.147967 | | protein_coding | | XP_008778779.1 | | abscisic acid receptor PYL8-like | | |
| LOC103698590 | -0.22566 | | | 0.147652 | | | -1.52834 | | | 0.126427 | | protein_coding | | XP_008778846.1 | | abscisic acid 8'-hydroxylase 1-like | | |
| LOC103713276 | 0.596897 | | | 0.186137 | | | 3.206762 | | | 0.001342 | | protein_coding | | XP_008798405.1 | | ras-related protein Rab7-like | | |
| LOC103697713 | 0.813876 | | | 0.543361 | | | 1.497854 | | | 0.134171 | | protein_coding | | XP_008777885.1 | | ras-related protein RABA1f-like | | |
| LOC103704138 | -0.22031 | | | 0.144739 | | | -1.52211 | | | 0.127981 | | protein_coding | | XP_008785548.1; XP_008785557.1; XP_026659398.1; XP_026659393.1; XP_008785540.1; XP_008785563.1; XP_026659395.1 | | ras-related protein RABA5a-like | | |
| LOC103708722 | 0.362793 | | | 0.302 | | | 1.201303 | | | 0.229634 | | protein_coding | | XP_008792003.1 | | ras-related protein RABB1c-like | | |
| LOC103710797 | 0.171929 | | | 0.27673 | | | 0.621289 | | | 0.534409 | | protein_coding | | XP_008794918.1 | | ras-related protein RABE1c-like | | |
| LOC103717597 | -0.66007 | | | 0.390138 | | | -1.69189 | | | 0.090666 | | protein_coding | | XP_008804271.2 | | ras-related protein RHN1 | | |
| LOC103719268 | 0.407386 | | | 0.223567 | | | 1.822212 | | | 0.068423 | | protein_coding | | XP_008806672.1; XP_008806652.1; XP_008806661.1 | | ras-related protein RIC1-like | | |
| LOC103723680 | -0.2309 | | | 0.203626 | | | -1.13392 | | | 0.256827 | | protein_coding | | XP_008812900.1; XP_008812891.1 | | ras-related protein Rab5 | | |
| LOC103696546 | 0.157625 | | | 0.232933 | | | 0.676698 | | | 0.498598 | | protein_coding | | XP_008776447.1; XP_008776438.1 | | ras-related protein RABE1c | | |
| LOC103698825 | -0.03972 | | | 0.184335 | | | -0.21546 | | | 0.82941 | | protein_coding | | XP_026657577.1; XP_026657574.1; XP_008779100.1 | | ras-related protein RABE1c-like | | |
| LOC103701624 | 0.553936 | | | 0.31314 | | | 1.768973 | | | 0.076898 | | protein_coding | | XP_008781983.1 | | ras-related protein RABA2a-like | | |
| LOC103701625 | 0.683173 | | | 0.674483 | | | 1.012884 | | | 0.311116 | | protein_coding | | XP_008781984.1 | | ras-related protein RABD1-like | | |
| LOC103701756 | 0.112957 | | | 0.140611 | | | 0.803328 | | | 0.421785 | | protein_coding | | XP_008782142.1 | | ras-related protein RABH1e | | |
| LOC103701773 | -0.00757 | | | 0.21121 | | | -0.03584 | | | 0.971406 | | protein_coding | | XP_008782165.1 | | ras-related protein Rab11D-like | | |
| LOC103702086 | 0.624392 | | | 0.257003 | | | 2.429508 | | | 0.015119 | | protein_coding | | XP_026658424.1; XP_008782600.1 | | ras-related protein Rab-21-like | | |
| LOC103702427 | 0.455371 | | | 3.483329 | | | 0.130729 | | | 0.89599 | | protein_coding | | XP_008783089.2 | | ras-related protein RGP1-like | | |
| LOC103702698 | 0.137349 | | | 0.130823 | | | 1.049888 | | | 0.293769 | | protein_coding | | XP_026658659.1; XP_026658658.1; XP_026658657.1; XP_008783456.1 | | ras-related protein RHN1-like | | |
| LOC103703429 | -0.22757 | | | 1.144992 | | | -0.19875 | | | 0.842458 | | protein_coding | | XP_008784489.2 | | ras-related protein RABA1f-like | | |
| LOC103703432 | -0.81753 | | | 0.341697 | | | -2.39257 | | | 0.016731 | | protein_coding | | XP_008784493.1 | | ras-related protein RABF1 | | |
| LOC103703574 | 0.290079 | | | 0.415361 | | | 0.698377 | | | 0.484941 | | protein_coding | | XP_017697366.1; XP_008784700.1; XP_017697369.1 | | ras-related protein RABH1b | | |
| LOC103704113 | -0.16434 | | | 0.398988 | | | -0.4119 | | | 0.680415 | | protein_coding | | XP_008785489.1 | | ras-related protein Rab11C | | |
| LOC103704764 | 0.018662 | | | 0.14677 | | | 0.127153 | | | 0.898819 | | protein_coding | | XP_026659518.1 | | ras-related protein RABA2a-like | | |
| LOC103704765 | 0.458976 | | | 0.716576 | | | 0.640513 | | | 0.521839 | | protein_coding | | XP_026659519.1; XP_026659520.1; XP_008786408.1 | | ras-related protein RABD1-like | | |
| LOC103705373 | 0.332622 | | | 0.3262 | | | 1.019686 | | | 0.307878 | | protein_coding | | XP_008787280.1 | | ras-related protein RGP1 | | |
| LOC103705403 | -1.17084 | | | 0.887746 | | | -1.31889 | | | 0.187205 | | protein_coding | | XP_026659795.1; XP_026659798.1; XP_026659796.1; XP_026659797.1; XP_026659799.1; XP_008787322.1 | | ras-related protein RABF2b-like | | |
| LOC103705502 | -0.30974 | | | 0.215803 | | | -1.43528 | | | 0.151207 | | protein_coding | | XP_008787455.1 | | ras-related protein RABE1c-like | | |
| LOC103705946 | 0.516776 | | | 0.247664 | | | 2.086601 | | | 0.036924 | | protein_coding | | XP_008788085.1 | | ras-related protein RABE1c | | |
| LOC103706102 | -1.05387 | | | 0.458224 | | | -2.2999 | | | 0.021454 | | protein_coding | | XP_008788327.1 | | ras-related protein RABC2a-like | | |
| LOC103706107 | -0.07955 | | | 0.220503 | | | -0.36078 | | | 0.718262 | | protein_coding | | XP_008788335.1 | | ras-related protein RABA2a-like | | |
| LOC103707349 | -0.35723 | | | 0.174315 | | | -2.04933 | | | 0.04043 | | protein_coding | | XP_008790017.1 | | ras-related protein RIC2 | | |
| LOC103708820 | 3.127118 | | | 0.74381 | | | 4.204187 | | | 2.62E-05 | | protein_coding | | XP_008792135.1; XP_008792136.1; XP_026661115.1 | | ras-related protein RABA5a-like | | |
| LOC103708973 | 0.652002 | | | 0.323036 | | | 2.018355 | | | 0.043554 | | protein_coding | | XP_008792325.1 | | ras-related protein RIC2-like | | |
| LOC103708986 | -0.18743 | | | 0.325232 | | | -0.57629 | | | 0.564417 | | protein_coding | | XP_008792342.1 | | ras-related protein Rab7 | | |
| LOC103709424 | -0.03679 | | | 0.218324 | | | -0.16851 | | | 0.866181 | | protein_coding | | XP_008792967.1 | | ras-related protein RABA5d-like | | |
| LOC103710081 | -1.38911 | | | 3.803607 | | | -0.36521 | | | 0.714957 | | protein_coding | | XP_008793910.1; XP_026661614.1 | | ras-related protein RHN1-like | | |
| LOC103710900 | 0.076787 | | | 0.673287 | | | 0.114048 | | | 0.9092 | | protein_coding | | XP_026661967.1 | | ras-related protein Rab7 | | |
| LOC103711253 | 0.239452 | | | 0.143019 | | | 1.674266 | | | 0.094078 | | protein_coding | | XP_008795552.1 | | ras-related protein RABH1e | | |
| LOC103712476 | -0.67333 | | | 0.350605 | | | -1.92047 | | | 0.054798 | | protein_coding | | XP_008797229.1; XP_026662489.1 | | ras-related protein RIC1-like | | |
| LOC103712942 | 0.232415 | | | 0.275274 | | | 0.844303 | | | 0.3985 | | protein_coding | | XP_008797878.1 | | ras-related protein RIC1 | | |
| LOC103713216 | -0.1089 | | | 0.299486 | | | -0.36362 | | | 0.716145 | | protein_coding | | XP_008798287.1 | | ras-related protein RABA1f | | |
| LOC103713777 | 1.956605 | | | 1.713099 | | | 1.142143 | | | 0.253394 | | protein_coding | | XP_008799023.1 | | ras-related protein RHN1-like | | |
| LOC103717982 | 0.652797 | | | 0.306131 | | | 2.132406 | | | 0.032973 | | protein_coding | | XP_026664599.1 | | ras-related protein Rab11A-like | | |
| LOC103719074 | -0.1714 | | | 0.803327 | | | -0.21336 | | | 0.831049 | | protein_coding | | XP_008806367.1 | | ras-related protein RABA3 | | |
| LOC103719292 | -0.42859 | | | 0.12006 | | | -3.56979 | | | 0.000357 | | protein_coding | | XP_008806692.1 | | ras-related protein RABF2b-like | | |
| LOC103720006 | -0.12841 | | | 0.211354 | | | -0.60755 | | | 0.543486 | | protein_coding | | XP_026665376.1; XP_008807734.1; XP_008807736.1 | | ras-related protein RABE1c-like | | |
| LOC103720815 | 0.118301 | | | 2.027127 | | | 0.058359 | | | 0.953463 | | protein_coding | | XP_008808948.1 | | ras-related protein Rab7 | | |
| LOC103721537 | -0.22607 | | | 0.176829 | | | -1.27847 | | | 0.201085 | | protein_coding | | XP_008810026.1 | | ras-related protein Rab7-like | | |
| LOC103722201 | 0.118899 | | | 0.178633 | | | 0.665606 | | | 0.505663 | | protein_coding | | XP_008810899.1 | | ras-related protein RABC1 | | |
| LOC113460945 | -0.10906 | | | 0.184606 | | | -0.5908 | | | 0.554657 | | protein_coding | | XP_026666238.1 | | ras-related protein RABC1-like | | |
| LOC103722541 | 0.217041 | | | 0.29747 | | | 0.729623 | | | 0.465621 | | protein_coding | | XP_008811352.1 | | ras-related protein Rab11C-like | | |
| LOC103722564 | 0.009746 | | | 0.321117 | | | 0.030352 | | | 0.975787 | | protein_coding | | XP_008811389.2 | | ras-related protein RABH1b-like | | |
| LOC103722651 | 0.119252 | | | 0.179471 | | | 0.664466 | | | 0.506392 | | protein_coding | | XP_008811507.1 | | ras-related protein Rab11C | | |
| LOC103722689 | -0.51021 | | | 0.200085 | | | -2.54995 | | | 0.010774 | | protein_coding | | XP_008811552.1 | | ras-related protein RABA1f | | |
| LOC103722762 | 0.125991 | | | 0.257594 | | | 0.489105 | | | 0.624767 | | protein_coding | | XP_008811658.1 | | ras-related protein RABA5e | | |
| LOC103723224 | -0.96735 | | | 0.356943 | | | -2.71009 | | | 0.006726 | | protein_coding | | XP_008812295.1 | | ras-related protein Rab7 | | |
| LOC103723446 | -0.77479 | | | 1.201194 | | | -0.64502 | | | 0.518915 | | protein_coding | | XP_008812576.1 | | ras-related protein RABC2a-like | | |
| LOC103696208 | -0.23217 | | | 0.193747 | | | -1.1983 | | | 0.230799 | | protein_coding | | XP_008775968.1; XP_026656601.1 | | ras-related protein RABB1c | | |
| LOC103697731 | 0.556766 | | | 0.24116 | | | 2.308704 | | | 0.02096 | | protein_coding | | XP_008777871.1 | | ras-related protein RIC2-like | | |
| LOC103698345 | 1.891878 | | | 0.377136 | | | 5.016428 | | | 5.26E-07 | | protein_coding | | XP_008778565.3 | | ras-related protein RABC1 | | |
| LOC103699426 | -0.28382 | | | 0.447257 | | | -0.63457 | | | 0.525706 | | protein_coding | | XP_008779666.1 | | ras-related protein RABA5d-like | | |
| **Abscisic acid associated differentially expressed genes in the Fungi treated group** | | | | | | | | | | | | | | | | | | |
| Gene_Symbol | | | log2FoldChange | | lfcSE | | | stat | | pvalue | | Type | | Protein_ID | | Product | | |
| LOC103707144 | | | -0.41857 | | 0.218644 | | | -1.91441 | | 0.055568 | | protein_coding | | XP_008789785.1 | | abscisic acid receptor PYL3-like | | |
| LOC103702957 | | | -0.71102 | | 0.210579 | | | -3.37648 | | 0.000734 | | protein_coding | | XP_008783837.1 | | abscisic acid receptor PYL8 | | |
| LOC103704693 | | | 1.121314 | | 0.096948 | | | 11.56611 | | 6.12E-31 | | protein_coding | | XP_008786319.1 | | abscisic acid receptor PYR1 | | |
| LOC103705585 | | | -0.9963 | | 0.783455 | | | -1.27168 | | 0.203487 | | protein_coding | | XP_026659822.1; XP_026659821.1 | | ABSCISIC ACID-INSENSITIVE 5-like protein 2 | | |
| LOC103708145 | | | -2.4683 | | 0.821085 | | | -3.00614 | | 0.002646 | | protein_coding | | XP_008791150.1 | | abscisic acid and environmental stress-inducible protein TAS14 | | |
| LOC103708332 | | | -0.51675 | | 0.175303 | | | -2.94774 | | 0.003201 | | protein_coding | | XP_008791430.1 | | abscisic acid receptor PYL8-like | | |
| LOC103709063 | | | 1.298552 | | 0.596459 | | | 2.177101 | | 0.029473 | | protein_coding | | XP_008792462.1 | | ABSCISIC ACID-INSENSITIVE 5-like protein 2 | | |
| LOC103713001 | | | 1.933941 | | 0.262409 | | | 7.369949 | | 1.71E-13 | | protein_coding | | XP_008797969.1 | | abscisic acid 8'-hydroxylase 1 | | |
| LOC103717496 | | | -0.16468 | | 0.227304 | | | -0.72447 | | 0.468776 | | protein_coding | | XP_008804142.2 | | abscisic acid receptor PYL4-like | | |
| LOC103719731 | | | 0.079403 | | 0.302223 | | | 0.262729 | | 0.792759 | | protein_coding | | XP_008807330.1 | | ABSCISIC ACID-INSENSITIVE 5-like protein 2 | | |
| LOC103698527 | | | 0.57798 | | 0.150796 | | | 3.832854 | | 0.000127 | | protein_coding | | XP_008778779.1 | | abscisic acid receptor PYL8-like | | |
| LOC103698590 | | | -0.07336 | | 0.201667 | | | -0.36375 | | 0.716044 | | protein_coding | | XP_008778846.1 | | abscisic acid 8'-hydroxylase 1-like | | |
| LOC103713276 | | | 0.112891 | | 0.157615 | | | 0.716246 | | 0.47384 | | protein_coding | | XP_008798405.1 | | ras-related protein Rab7-like | | |
| LOC103697713 | | | -0.38065 | | 0.257726 | | | -1.47695 | | 0.139689 | | protein_coding | | XP_008777885.1 | | ras-related protein RABA1f-like | | |
| LOC103704138 | | | -0.13456 | | 0.143104 | | | -0.94027 | | 0.347079 | | protein_coding | | XP_008785548.1; XP_008785557.1; XP_026659398.1; XP_026659393.1; XP_008785540.1; XP_008785563.1; XP_026659395.1 | | ras-related protein RABA5a-like | | |
| LOC103706102 | | | 0.113429 | | 0.121202 | | | 0.935862 | | 0.349344 | | protein_coding | | XP_008788327.1 | | ras-related protein RABC2a-like | | |
| LOC103697731 | | | 0.775062 | | 0.835781 | | | 0.92735 | | 0.353745 | | protein_coding | | XP_008777871.1 | | ras-related protein RIC2-like | | |
| LOC103717982 | | | 0.096741 | | 0.113972 | | | 0.84882 | | 0.395982 | | protein_coding | | XP_026664599.1 | | ras-related protein Rab11A-like | | |
| LOC103713777 | | | -0.21254 | | 0.251805 | | | -0.84407 | | 0.398628 | | protein_coding | | XP_008799023.1 | | ras-related protein RHN1-like | | |
| LOC103710081 | | | 0.463075 | | 0.558634 | | | 0.828942 | | 0.407137 | | protein_coding | | XP_008793910.1; XP_026661614.1 | | ras-related protein RHN1-like | | |
| LOC103709424 | | | 0.244436 | | 0.30234 | | | 0.80848 | | 0.418815 | | protein_coding | | XP_008792967.1 | | ras-related protein RABA5d-like | | |
| LOC103699426 | | | -0.28328 | | 0.37792 | | | -0.74959 | | 0.453503 | | protein_coding | | XP_008779666.1 | | ras-related protein RABA5d-like | | |
| LOC103703429 | | | -0.30732 | | 0.412612 | | | -0.74482 | | 0.456378 | | protein_coding | | XP_008784489.2 | | ras-related protein RABA1f-like | | |
| LOC103701773 | | | 0.13399 | | 0.183227 | | | 0.731279 | | 0.464609 | | protein_coding | | XP_008782165.1 | | ras-related protein Rab11D-like | | |
| LOC103704765 | | | 0.104807 | | 0.144219 | | | 0.726718 | | 0.467399 | | protein_coding | | XP_026659519.1; XP_026659520.1; XP_008786408.1 | | ras-related protein RABD1-like | | |
| LOC103713276 | | | 0.112891 | | 0.157615 | | | 0.716246 | | 0.47384 | | protein_coding | | XP_008798405.1 | | ras-related protein Rab7-like | | |
| LOC103708722 | | | -0.11041 | | 0.157411 | | | -0.70141 | | 0.483049 | | protein_coding | | XP_008792003.1 | | ras-related protein RABB1c-like | | |
| LOC103719292 | | | -0.08456 | | 0.126939 | | | -0.66613 | | 0.505329 | | protein_coding | | XP_008806692.1 | | ras-related protein RABF2b-like | | |
| LOC103702086 | | | 0.100515 | | 0.153614 | | | 0.654334 | | 0.512897 | | protein_coding | | XP_026658424.1; XP_008782600.1 | | ras-related protein Rab-21-like | | |
| LOC103698345 | | | 0.103057 | | 0.171419 | | | 0.601198 | | 0.547708 | | protein_coding | | XP_008778565.3 | | ras-related protein RABC1 | | |
| LOC103703432 | | | -0.05926 | | 0.113999 | | | -0.51987 | | 0.603156 | | protein_coding | | XP_008784493.1 | | ras-related protein RABF1 | | |
| LOC103698825 | | | -0.09092 | | 0.188448 | | | -0.48248 | | 0.629466 | | protein_coding | | XP_026657577.1; XP_026657574.1; XP_008779100.1 | | ras-related protein RABE1c-like | | |
| LOC103696208 | | | 0.072464 | | 0.153981 | | | 0.470607 | | 0.637922 | | protein_coding | | XP_008775968.1; XP_026656601.1 | | ras-related protein RABB1c | | |
| LOC103710797 | | | 0.16936 | | 0.366734 | | | 0.461806 | | 0.644221 | | protein_coding | | XP_008794918.1 | | ras-related protein RABE1c-like | | |
| LOC103721537 | | | 0.045131 | | 0.117908 | | | 0.382765 | | 0.701894 | | protein_coding | | XP_008810026.1 | | ras-related protein Rab7-like | | |
| LOC103720815 | | | -0.2685 | | 0.708168 | | | -0.37915 | | 0.704577 | | protein_coding | | XP_008808948.1 | | ras-related protein Rab7 | | |
| LOC103705373 | | | -0.0685 | | 0.193094 | | | -0.35476 | | 0.72277 | | protein_coding | | XP_008787280.1 | | ras-related protein RGP1 | | |
| LOC103696546 | | | 0.049322 | | 0.15081 | | | 0.327047 | | 0.743633 | | protein_coding | | XP_008776447.1; XP_008776438.1 | | ras-related protein RABE1c | | |
| LOC103723224 | | | 0.034264 | | 0.149782 | | | 0.228757 | | 0.819058 | | protein_coding | | XP_008812295.1 | | ras-related protein Rab7 | | |
| LOC103705502 | | | -0.03492 | | 0.271423 | | | -0.12867 | | 0.897616 | | protein_coding | | XP_008787455.1 | | ras-related protein RABE1c-like | | |
| LOC103722201 | | | 0.018144 | | 0.144798 | | | 0.125308 | | 0.90028 | | protein_coding | | XP_008810899.1 | | ras-related protein RABC1 | | |
| LOC103702698 | | | 0.010042 | | 0.122612 | | | 0.0819 | | 0.934727 | | protein_coding | | XP_026658659.1; XP_026658658.1; XP_026658657.1; XP_008783456.1 | | ras-related protein RHN1-like | | |
| LOC103722541 | | | -0.00839 | | 0.128224 | | | -0.06541 | | 0.94785 | | protein_coding | | XP_008811352.1 | | ras-related protein Rab11C-like | | |
| LOC103705403 | | | -0.00786 | | 0.183183 | | | -0.04289 | | 0.965786 | | protein_coding | | XP_026659795.1; XP_026659798.1; XP_026659796.1; XP_026659797.1; XP_026659799.1; XP_008787322.1 | | ras-related protein RABF2b-like | | |
| LOC103723680 | | | 0.00108 | | 0.302781 | | | 0.003566 | | 0.997155 | | protein_coding | | XP_008812900.1; XP_008812891.1 | | ras-related protein Rab5 | | |
| **Abscisic acid associated differentially expressed genes in the Fungi+Salt treated group** | | | | | | | | | | | | | | | | | | |
| **Gene_Symbol** | | **log2FoldChange** | | | | **lfcSE** | | | **stat** | | **pvalue** | | **Type** | | **Gene_Symbol** | **Product** | | |
| LOC103707144 | | 0.185787 | | | | 0.179254 | | | 1.036448 | | 0.299993 | | protein_coding | | XP_008789785.1 | abscisic acid receptor PYL3-like | | |
| LOC103702957 | | 0.517842 | | | | 0.149148 | | | 3.472005 | | 0.000517 | | protein_coding | | XP_008783837.1 | abscisic acid receptor PYL8 | | |
| LOC103704693 | | -1.11408 | | | | 0.103511 | | | -10.7629 | | 5.15E-27 | | protein_coding | | XP_008786319.1 | abscisic acid receptor PYR1 | | |
| LOC103705585 | | 0.492358 | | | | 0.692545 | | | 0.71094 | | 0.477122 | | protein_coding | | XP_026659822.1; XP_026659821.1 | ABSCISIC ACID-INSENSITIVE 5-like protein 2 | | |
| LOC103706891 | | -0.02521 | | | | 2.494786 | | | -0.01011 | | 0.991937 | | protein_coding | | XP_008789382.1 | abscisic acid receptor PYL12-like | | |
| LOC103708145 | | -2.60475 | | | | 0.780136 | | | -3.33884 | | 0.000841 | | protein_coding | | XP_008791150.1 | abscisic acid and environmental stress-inducible protein TAS14 | | |
| LOC103708332 | | -0.07859 | | | | 0.164291 | | | -0.47838 | | 0.63238 | | protein_coding | | XP_008791430.1 | abscisic acid receptor PYL8-like | | |
| LOC103709063 | | -1.18129 | | | | 1.077986 | | | -1.09583 | | 0.273152 | | protein_coding | | XP_008792462.1 | ABSCISIC ACID-INSENSITIVE 5-like protein 2 | | |
| LOC103711815 | | 0.597193 | | | | 0.1559 | | | 3.830607 | | 0.000128 | | protein_coding | | XP_008796322.2 | abscisic acid receptor PYR1-like | | |
| LOC103713001 | | 0.997707 | | | | 0.270445 | | | 3.689132 | | 0.000225 | | protein_coding | | XP_008797969.1 | abscisic acid 8'-hydroxylase 1 | | |
| LOC103714404 | | -0.39514 | | | | 0.171028 | | | -2.31039 | | 0.020867 | | protein_coding | | XP_008799865.1 | abscisic acid receptor PYL4 | | |
| LOC103716978 | | -1.20809 | | | | 3.124812 | | | -0.38661 | | 0.699043 | | protein_coding | | XP_008803422.3 | ABSCISIC ACID-INSENSITIVE 5-like protein 1 | | |
| LOC103717496 | | -0.53396 | | | | 0.238435 | | | -2.23942 | | 0.025129 | | protein_coding | | XP_008804142.2 | abscisic acid receptor PYL4-like | | |
| LOC103719731 | | 0.099639 | | | | 0.268459 | | | 0.371153 | | 0.710524 | | protein_coding | | XP_008807330.1 | ABSCISIC ACID-INSENSITIVE 5-like protein 2 | | |
| LOC103719844 | | 2.353583 | | | | 1.346163 | | | 1.748364 | | 0.080401 | | protein_coding | | XP_008807492.1 | abscisic acid and environmental stress-inducible protein-like | | |
| LOC103696437 | | -0.3797 | | | | 0.159266 | | | -2.38408 | | 0.017122 | | protein_coding | | XP_008776287.1 | abscisic acid receptor PYL4-like | | |
| LOC103698268 | | 6.437003 | | | | 1.528617 | | | 4.210999 | | 2.54E-05 | | protein_coding | | XP_026657338.1 | abscisic acid 8'-hydroxylase 3-like | | |
| LOC103698527 | | 0.607928 | | | | 0.123739 | | | 4.912993 | | 8.97E-07 | | protein_coding | | XP_008778779.1 | abscisic acid receptor PYL8-like | | |
| LOC103698590 | | -0.97144 | | | | 0.206988 | | | -4.6932 | | 2.69E-06 | | protein_coding | | XP_008778846.1 | abscisic acid 8'-hydroxylase 1-like | | |
| LOC103713276 | | -0.15599 | | | | 0.13306 | | | -1.17236 | | 0.241053 | | protein_coding | | XP_008798405.1 | ras-related protein Rab7-like | | |
| LOC103697713 | | -0.20086 | | | | 0.258107 | | | -0.77822 | | 0.436437 | | protein_coding | | XP_008777885.1 | ras-related protein RABA1f-like | | |
| LOC103704138 | | -0.11252 | | | | 0.130155 | | | -0.86452 | | 0.387302 | | protein_coding | | XP_008785548.1; XP_008785557.1; XP_026659398.1; XP_026659393.1; XP_008785540.1; XP_008785563.1; XP_026659395.1 | ras-related protein RABA5a-like | | |
| LOC103708722 | | 0.264271 | | | | 0.144374 | | | 1.830457 | | 0.067182 | | protein_coding | | XP_008792003.1 | ras-related protein RABB1c-like | | |
| LOC103710797 | | -0.51131 | | | | 0.30976 | | | -1.65065 | | 0.09881 | | protein_coding | | XP_008794918.1 | ras-related protein RABE1c-like | | |
| LOC103717597 | | 0.033047 | | | | 0.126004 | | | 0.262271 | | 0.793113 | | protein_coding | | XP_008804271.2 | ras-related protein RHN1 | | |
| LOC103719268 | | -0.45401 | | | | 0.139425 | | | -3.25629 | | 0.001129 | | protein_coding | | XP_008806672.1; XP_008806652.1; XP_008806661.1 | ras-related protein RIC1-like | | |
| LOC103723680 | | 0.081867 | | | | 0.291711 | | | 0.280644 | | 0.778984 | | protein_coding | | XP_008812900.1; XP_008812891.1 | ras-related protein Rab5 | | |
| LOC103696546 | | 0.024842 | | | | 0.142872 | | | 0.173876 | | 0.861963 | | protein_coding | | XP_008776447.1; XP_008776438.1 | ras-related protein RABE1c | | |
| LOC103698825 | | 0.123958 | | | | 0.173263 | | | 0.715434 | | 0.474341 | | protein_coding | | XP_026657577.1; XP_026657574.1; XP_008779100.1 | ras-related protein RABE1c-like | | |
| LOC103701624 | | 0.106321 | | | | 0.166508 | | | 0.638532 | | 0.523128 | | protein_coding | | XP_008781983.1 | ras-related protein RABA2a-like | | |
| LOC103701625 | | -0.45189 | | | | 0.876891 | | | -0.51533 | | 0.606322 | | protein_coding | | XP_008781984.1 | ras-related protein RABD1-like | | |
| LOC103701756 | | 0.195523 | | | | 0.120473 | | | 1.622962 | | 0.104597 | | protein_coding | | XP_008782142.1 | ras-related protein RABH1e | | |
| LOC103701773 | | -0.06942 | | | | 0.166145 | | | -0.41782 | | 0.67608 | | protein_coding | | XP_008782165.1 | ras-related protein Rab11D-like | | |
| LOC103702086 | | 0.370266 | | | | 0.14448 | | | 2.562758 | | 0.010384 | | protein_coding | | XP_026658424.1; XP_008782600.1 | ras-related protein Rab-21-like | | |
| LOC103702427 | | 0.723566 | | | | 0.138572 | | | 5.221573 | | 1.77E-07 | | protein_coding | | XP_008783089.2 | ras-related protein RGP1-like | | |
| LOC103702698 | | 0.117506 | | | | 0.112285 | | | 1.046494 | | 0.295333 | | protein_coding | | XP_026658659.1; XP_026658658.1; XP_026658657.1; XP_008783456.1 | ras-related protein RHN1-like | | |
| LOC103703429 | | 0.957249 | | | | 0.36745 | | | 2.605114 | | 0.009184 | | protein_coding | | XP_008784489.2 | ras-related protein RABA1f-like | | |
| LOC103703432 | | 0.212876 | | | | 0.098065 | | | 2.170777 | | 0.029948 | | protein_coding | | XP_008784493.1 | ras-related protein RABF1 | | |
| LOC103703574 | | 0.321342 | | | | 0.318984 | | | 1.007392 | | 0.313747 | | protein_coding | | XP_017697366.1; XP_008784700.1; XP_017697369.1 | ras-related protein RABH1b | | |
| LOC103704113 | | 0.672936 | | | | 0.179981 | | | 3.738932 | | 0.000185 | | protein_coding | | XP_008785489.1 | ras-related protein Rab11C | | |
| LOC103704764 | | -0.68083 | | | | 0.203809 | | | -3.34055 | | 0.000836 | | protein_coding | | XP_026659518.1 | ras-related protein RABA2a-like | | |
| LOC103704765 | | -0.35734 | | | | 0.150991 | | | -2.36664 | | 0.01795 | | protein_coding | | XP_026659519.1; XP_026659520.1; XP_008786408.1 | ras-related protein RABD1-like | | |
| LOC103705373 | | 0.17163 | | | | 0.172768 | | | 0.993409 | | 0.32051 | | protein_coding | | XP_008787280.1 | ras-related protein RGP1 | | |
| LOC103705403 | | 0.286566 | | | | 0.160702 | | | 1.783217 | | 0.074551 | | protein_coding | | XP_026659795.1; XP_026659798.1; XP_026659796.1; XP_026659797.1; XP_026659799.1; XP_008787322.1 | ras-related protein RABF2b-like | | |
| LOC103705502 | | 0.17867 | | | | 0.259227 | | | 0.689244 | | 0.49067 | | protein_coding | | XP_008787455.1 | ras-related protein RABE1c-like | | |
| LOC103705946 | | 0.289077 | | | | 0.138102 | | | 2.093205 | | 0.036331 | | protein_coding | | XP_008788085.1 | ras-related protein RABE1c | | |
| LOC103706102 | | -0.0476 | | | | 0.115375 | | | -0.41257 | | 0.679921 | | protein_coding | | XP_008788327.1 | ras-related protein RABC2a-like | | |
| LOC103706107 | | 0.43241 | | | | 0.225022 | | | 1.921637 | | 0.054651 | | protein_coding | | XP_008788335.1 | ras-related protein RABA2a-like | | |
| LOC103707349 | | 0.193583 | | | | 0.104072 | | | 1.860085 | | 0.062873 | | protein_coding | | XP_008790017.1 | ras-related protein RIC2 | | |
| LOC103708973 | | -2.07741 | | | | 0.32992 | | | -6.29672 | | 3.04E-10 | | protein_coding | | XP_008792325.1 | ras-related protein RIC2-like | | |
| LOC103708986 | | 0.044732 | | | | 0.118864 | | | 0.376328 | | 0.706673 | | protein_coding | | XP_008792342.1 | ras-related protein Rab7 | | |
| LOC103709424 | | 0.321842 | | | | 0.293176 | | | 1.097778 | | 0.272301 | | protein_coding | | XP_008792967.1 | ras-related protein RABA5d-like | | |
| LOC103710081 | | -0.36207 | | | | 0.581788 | | | -0.62234 | | 0.53372 | | protein_coding | | XP_008793910.1; XP_026661614.1 | ras-related protein RHN1-like | | |
| LOC103710900 | | 0.026664 | | | | 0.158135 | | | 0.168614 | | 0.866101 | | protein_coding | | XP_026661967.1 | ras-related protein Rab7 | | |
| LOC103711253 | | 0.17693 | | | | 0.244138 | | | 0.724711 | | 0.468629 | | protein_coding | | XP_008795552.1 | ras-related protein RABH1e | | |
| LOC103712476 | | -0.21744 | | | | 0.190237 | | | -1.143 | | 0.253037 | | protein_coding | | XP_008797229.1; XP_026662489.1 | ras-related protein RIC1-like | | |
| LOC103712942 | | -0.06864 | | | | 0.082407 | | | -0.83295 | | 0.404876 | | protein_coding | | XP_008797878.1 | ras-related protein RIC1 | | |
| LOC103713216 | | -0.07327 | | | | 0.299644 | | | -0.24453 | | 0.80682 | | protein_coding | | XP_008798287.1 | ras-related protein RABA1f | | |
| LOC103713777 | | -0.46705 | | | | 0.235605 | | | -1.98236 | | 0.047439 | | protein_coding | | XP_008799023.1 | ras-related protein RHN1-like | | |
| LOC103717982 | | -0.33149 | | | | 0.123868 | | | -2.67614 | | 0.007448 | | protein_coding | | XP_026664599.1 | ras-related protein Rab11A-like | | |
| LOC103719074 | | -0.25222 | | | | 0.275216 | | | -0.91646 | | 0.359425 | | protein_coding | | XP_008806367.1 | ras-related protein RABA3 | | |
| LOC103719292 | | 0.287574 | | | | 0.114469 | | | 2.512249 | | 0.011996 | | protein_coding | | XP_008806692.1 | ras-related protein RABF2b-like | | |
| LOC103720006 | | 0.223304 | | | | 0.250368 | | | 0.891903 | | 0.372445 | | protein_coding | | XP_026665376.1; XP_008807734.1; XP_008807736.1 | ras-related protein RABE1c-like | | |
| LOC103720815 | | 0.343833 | | | | 0.662331 | | | 0.519125 | | 0.603674 | | protein_coding | | XP_008808948.1 | ras-related protein Rab7 | | |
| LOC103721537 | | -0.31748 | | | | 0.086221 | | | -3.68213 | | 0.000231 | | protein_coding | | XP_008810026.1 | ras-related protein Rab7-like | | |
| LOC103722201 | | 0.09463 | | | | 0.124124 | | | 0.762385 | | 0.44583 | | protein_coding | | XP_008810899.1 | ras-related protein RABC1 | | |
| LOC113460945 | | 0.337676 | | | | 2.022383 | | | 0.166969 | | 0.867394 | | protein_coding | | XP_026666238.1 | ras-related protein RABC1-like | | |
| LOC103722541 | | 0.015788 | | | | 0.11937 | | | 0.132258 | | 0.89478 | | protein_coding | | XP_008811352.1 | ras-related protein Rab11C-like | | |
| LOC103722564 | | 0.23823 | | | | 0.131832 | | | 1.807068 | | 0.070752 | | protein_coding | | XP_008811389.2 | ras-related protein RABH1b-like | | |
| LOC103722651 | | -1.22343 | | | | 0.128055 | | | -9.55399 | | 1.25E-21 | | protein_coding | | XP_008811507.1 | ras-related protein Rab11C | | |
| LOC103722689 | | 0.573258 | | | | 0.230479 | | | 2.487245 | | 0.012874 | | protein_coding | | XP_008811552.1 | ras-related protein RABA1f | | |
| LOC103722762 | | -1.49 | | | | 0.387865 | | | -3.84155 | | 0.000122 | | protein_coding | | XP_008811658.1 | ras-related protein RABA5e | | |
| LOC103723224 | | -0.00324 | | | | 0.144132 | | | -0.02245 | | 0.982091 | | protein_coding | | XP_008812295.1 | ras-related protein Rab7 | | |
| LOC103723446 | | -1.03061 | | | | 0.190777 | | | -5.40217 | | 6.58E-08 | | protein_coding | | XP_008812576.1 | ras-related protein RABC2a-like | | |
| LOC103696208 | | 0.659446 | | | | 0.132315 | | | 4.983927 | | 6.23E-07 | | protein_coding | | XP_008775968.1; XP_026656601.1 | ras-related protein RABB1c | | |
| LOC103697731 | | -0.62197 | | | | 0.785699 | | | -0.79161 | | 0.428585 | | protein_coding | | XP_008777871.1 | ras-related protein RIC2-like | | |
| LOC103698345 | | -0.14379 | | | | 0.175034 | | | -0.82149 | | 0.41137 | | protein_coding | | XP_008778565.3 | ras-related protein RABC1 | | |
| LOC103699426 | | -1.473 | | | | 0.514134 | | | -2.86501 | | 0.00417 | | protein_coding | | XP_008779666.1 | ras-related protein RABA5d-like | | |

Table S6: Differentially expressed ethylene-responsive transcription factors for the date palm seedling groups

| Characteristics of differentially expressed ethylene-responisive transcription factors in the salt group | | | | | | | | | | | | | | | | | | |
| --- | --- | --- | --- | --- | --- | --- | --- | --- | --- | --- | --- | --- | --- | --- | --- | --- | --- | --- |
| Gene_Symbol | | log2FoldChange | | | lfcSE | | | stat | | | pvalue | | Type | | Protein_ID | | product | |
| LOC113462365 | | -0.70051 | | | 1.30881 | | | -0.53522 | | | 0.592496 | | protein_coding | | XP_026658220.1 | | ethylene-responsive transcription factor ERF107-like | |
| LOC113461956 | | 0.344719 | | | 0.842613 | | | 0.409108 | | | 0.682461 | | protein_coding | | XP_026657762.1 | | ethylene-responsive transcription factor CRF4-like | |
| LOC108510684 | | -0.31807 | | | 3.878423 | | | -0.08201 | | | 0.934639 | | protein_coding | | XP_017697344.1 | | ethylene-responsive transcription factor ERF017-like | |
| LOC103724191 | | 0.25598 | | | 0.229158 | | | 1.117046 | | | 0.263975 | | protein_coding | | XP_008813598.1 | | ethylene-responsive transcription factor 2 | |
| LOC103723824 | | -0.05123 | | | 0.440011 | | | -0.11644 | | | 0.907307 | | protein_coding | | XP_008813098.1 | | ethylene-responsive transcription factor ERN1 | |
| LOC103722685 | | -1.62734 | | | 1.328227 | | | -1.2252 | | | 0.220501 | | protein_coding | | XP_008811549.2 | | ethylene-responsive transcription factor 1B-like | |
| LOC103722581 | | -1.02556 | | | 3.611213 | | | -0.28399 | | | 0.776415 | | protein_coding | | XP_008811407.1 | | ethylene-responsive transcription factor ERF071-like | |
| LOC103722354 | | 2.187414 | | | 0.926622 | | | 2.360632 | | | 0.018244 | | protein_coding | | XP_017701969.1; XP_008811102.1 | | ethylene-responsive transcription factor ERF113-like | |
| LOC103722021 | | 1.323972 | | | 2.279668 | | | 0.580774 | | | 0.561393 | | protein_coding | | XP_008810659.1 | | ethylene-responsive transcription factor TINY-like | |
| LOC103722008 | | 0.095233 | | | 0.152322 | | | 0.625206 | | | 0.531836 | | protein_coding | | XP_008810643.1; XP_026666181.1 | | ethylene-responsive transcription factor-like protein At4g13040 | |
| LOC103721225 | | 0.363255 | | | 0.23709 | | | 1.532143 | | | 0.125487 | | protein_coding | | XP_026665879.1 | | AP2-like ethylene-responsive transcription factor At2g41710 | |
| LOC103721201 | | 0.44916 | | | 1.488963 | | | 0.301659 | | | 0.762912 | | protein_coding | | XP_008809528.1 | | ethylene-responsive transcription factor LEP-like | |
| LOC103721161 | | -0.32538 | | | 0.46551 | | | -0.69897 | | | 0.484569 | | protein_coding | | XP_008809462.1 | | ethylene-responsive transcription factor 2-like | |
| LOC103720343 | | 2.728892 | | | 0.867398 | | | 3.146067 | | | 0.001655 | | protein_coding | | XP_008808223.1 | | ethylene-responsive transcription factor ERF003-like | |
| LOC103720309 | | 0.25823 | | | 0.193597 | | | 1.333854 | | | 0.182252 | | protein_coding | | XP_008808175.1 | | ethylene-responsive transcription factor ERF060-like | |
| LOC103720224 | | -0.21063 | | | 0.139772 | | | -1.50697 | | | 0.131819 | | protein_coding | | XP_008808053.1 | | AP2-like ethylene-responsive transcription factor AIL1 | |
| LOC103719993 | | 0.13014 | | | 0.143038 | | | 0.909824 | | | 0.362915 | | protein_coding | | XP_008807712.1; XP_008807713.1 | | AP2-like ethylene-responsive transcription factor BBM1 | |
| LOC103719783 | | 0.725515 | | | 0.648141 | | | 1.119379 | | | 0.262979 | | protein_coding | | XP_008807399.1 | | ethylene-responsive transcription factor ERF071-like | |
| LOC103719666 | | -1.17552 | | | 1.420487 | | | -0.82754 | | | 0.407929 | | protein_coding | | XP_008807234.1 | | ethylene-responsive transcription factor ERF023-like | |
| LOC103719441 | | 1.442496 | | | 1.164976 | | | 1.23822 | | | 0.215634 | | protein_coding | | XP_008806912.1 | | ethylene-responsive transcription factor 1B-like | |
| LOC103719025 | | -0.26174 | | | 0.783111 | | | -0.33423 | | | 0.738202 | | protein_coding | | XP_008806298.1 | | ethylene-responsive transcription factor ERF003-like | |
| LOC103718991 | | -0.07546 | | | 0.1771 | | | -0.4261 | | | 0.670033 | | protein_coding | | XP_026665033.1 | | ethylene-responsive transcription factor ERF039-like | |
| LOC103717663 | | -0.70837 | | | 0.582931 | | | -1.21519 | | | 0.224293 | | protein_coding | | XP_008804361.1 | | ethylene-responsive transcription factor ERN1-like | |
| LOC103717533 | | 0.146021 | | | 1.801516 | | | 0.081055 | | | 0.935399 | | protein_coding | | XP_008804192.2 | | AP2-like ethylene-responsive transcription factor BBM2 | |
| LOC103717275 | | -0.78231 | | | 0.487795 | | | -1.60377 | | | 0.108766 | | protein_coding | | XP_008803818.3 | | ethylene-responsive transcription factor ERF034-like | |
| LOC103717228 | | 0.944511 | | | 0.296119 | | | 3.189635 | | | 0.001425 | | protein_coding | | XP_008803760.1 | | ethylene-responsive transcription factor RAP2-4-like | |
| LOC103717086 | | 0.279745 | | | 0.313521 | | | 0.892266 | | | 0.37225 | | protein_coding | | XP_008803555.1 | | ethylene-responsive transcription factor CRF1-like | |
| LOC103717048 | | -0.33166 | | | 0.351237 | | | -0.94426 | | | 0.345036 | | protein_coding | | XP_026664229.1; XP_008803499.1; XP_008803501.1; XP_008803500.1 | | ethylene-responsive transcription factor-like protein At4g13040 | |
| LOC103717044 | | 1.079567 | | | 0.182914 | | | 5.902054 | | | 3.59E-09 | | protein_coding | | XP_008803496.1 | | ethylene-responsive transcription factor 3-like | |
| LOC103716571 | | 2.184587 | | | 0.535993 | | | 4.075779 | | | 4.59E-05 | | protein_coding | | XP_008802835.1 | | ethylene-responsive transcription factor 11-like | |
| LOC103716427 | | -0.18429 | | | 0.211742 | | | -0.87034 | | | 0.384115 | | protein_coding | | XP_008802631.1; XP_008802632.1 | | AP2-like ethylene-responsive transcription factor At2g41710 | |
| LOC103716011 | | -2.4072 | | | 0.442586 | | | -5.43894 | | | 5.36E-08 | | protein_coding | | XP_008802060.1 | | ethylene-responsive transcription factor ERF014-like | |
| LOC103715808 | | -1.32161 | | | 0.412659 | | | -3.20267 | | | 0.001362 | | protein_coding | | XP_008801788.1 | | ethylene-responsive transcription factor ERF105-like | |
| LOC103715562 | | -0.54967 | | | 3.742077 | | | -0.14689 | | | 0.88322 | | protein_coding | | XP_008801457.1 | | ethylene-responsive transcription factor ERF015 | |
| LOC103714855 | | -0.80518 | | | 3.929768 | | | -0.20489 | | | 0.837656 | | protein_coding | | XP_008800510.2 | | ethylene-responsive transcription factor ERF016-like | |
| LOC103714826 | | 0.41792 | | | 0.232908 | | | 1.79436 | | | 0.072756 | | protein_coding | | XP_008800472.1; XP_008800471.1 | | ethylene-responsive transcription factor 1-like | |
| LOC103713952 | | 0.837016 | | | 0.254902 | | | 3.283678 | | | 0.001025 | | protein_coding | | XP_008799235.1 | | ethylene-responsive transcription factor CRF2-like | |
| LOC103713506 | | 2.983774 | | | 2.473831 | | | 1.206135 | | | 0.227765 | | protein_coding | | XP_008798683.1 | | ethylene-responsive transcription factor CRF1-like | |
| LOC103713218 | | -0.1158 | | | 0.299604 | | | -0.3865 | | | 0.699127 | | protein_coding | | XP_008798289.3 | | ethylene-responsive transcription factor 2-like | |
| LOC103712913 | | -0.01003 | | | 0.145522 | | | -0.06894 | | | 0.945036 | | protein_coding | | XP_008797843.1 | | ethylene-responsive transcription factor CRF2 | |
| LOC103712588 | | 0.614209 | | | 0.203512 | | | 3.01805 | | | 0.002544 | | protein_coding | | XP_008797374.1 | | ethylene-responsive transcription factor 1-like | |
| LOC103712528 | | -1.51556 | | | 0.913589 | | | -1.65891 | | | 0.097134 | | protein_coding | | XP_008797298.1 | | ethylene-responsive transcription factor ERF014-like | |
| LOC103712373 | | 0.682631 | | | 1.125205 | | | 0.606672 | | | 0.544068 | | protein_coding | | XP_008797100.1 | | ethylene-responsive transcription factor LEP-like | |
| LOC103711902 | | 1.070503 | | | 0.710259 | | | 1.507202 | | | 0.131759 | | protein_coding | | XP_008796443.1 | | ethylene-responsive transcription factor 3-like | |
| LOC103711691 | | 1.939666 | | | 1.050565 | | | 1.846307 | | | 0.064848 | | protein_coding | | XP_017699428.1 | | ethylene-responsive transcription factor ERF039-like | |
| LOC103711505 | | 0.574276 | | | 0.300383 | | | 1.911811 | | | 0.0559 | | protein_coding | | XP_008795894.1; XP_026662179.1; XP_008795895.1 | | AP2-like ethylene-responsive transcription factor TOE3 | |
| LOC103711503 | | 0.262852 | | | 0.239255 | | | 1.09863 | | | 0.271929 | | protein_coding | | XP_017699377.1 | | ethylene-responsive transcription factor ERF011-like | |
| LOC103711129 | | -0.80205 | | | 2.161939 | | | -0.37099 | | | 0.710647 | | protein_coding | | XP_008795369.1 | | ethylene-responsive transcription factor ERF027 | |
| LOC103711088 | | -1.949 | | | 0.56063 | | | -3.47644 | | | 0.000508 | | protein_coding | | XP_008795312.1 | | ethylene-responsive transcription factor ERF038-like | |
| LOC103710089 | | 2.744389 | | | 1.167266 | | | 2.351125 | | | 0.018717 | | protein_coding | | XP_008793924.1; XP_008793923.1; XP_026661617.1 | | ethylene-responsive transcription factor ERF115-like | |
| LOC103709666 | | -0.42482 | | | 0.420925 | | | -1.00925 | | | 0.312855 | | protein_coding | | XP_008793348.3 | | AP2-like ethylene-responsive transcription factor AIL5 | |
| LOC103709326 | | -3.17597 | | | 1.522371 | | | -2.0862 | | | 0.036961 | | protein_coding | | XP_008792845.1 | | ethylene-responsive transcription factor ERF017-like | |
| LOC103709065 | | 0.31501 | | | 1.835682 | | | 0.171604 | | | 0.863749 | | protein_coding | | XP_026661158.1 | | ethylene-responsive transcription factor WRI1-like | |
| LOC103708748 | | -3.88485 | | | 3.422319 | | | -1.13515 | | | 0.256311 | | protein_coding | | XP_008792041.1 | | ethylene-responsive transcription factor WIN1-like | |
| LOC103708547 | | -0.15278 | | | 0.51012 | | | -0.2995 | | | 0.76456 | | protein_coding | | XP_008791735.1 | | AP2-like ethylene-responsive transcription factor AIL1 | |
| LOC103708353 | | -1.3011 | | | 2.109062 | | | -0.61691 | | | 0.537295 | | protein_coding | | XP_008791463.1 | | ethylene-responsive transcription factor ERF069-like | |
| LOC103708330 | | -1.44077 | | | 2.520101 | | | -0.57171 | | | 0.567519 | | protein_coding | | XP_008791428.1 | | ethylene-responsive transcription factor ERF061 | |
| LOC103708042 | | -0.17723 | | | 0.396455 | | | -0.44704 | | | 0.65485 | | protein_coding | | XP_008791015.1 | | ethylene-responsive transcription factor ERF113-like | |
| LOC103707892 | | 0.01706 | | | 0.206249 | | | 0.082717 | | | 0.934076 | | protein_coding | | XP_017698527.1 | | ethylene-responsive transcription factor RAP2-13-like | |
| LOC103707419 | | -0.34482 | | | 1.516136 | | | -0.22743 | | | 0.820086 | | protein_coding | | XP_008790117.1 | | AP2-like ethylene-responsive transcription factor At1g16060 | |
| LOC103707269 | | -0.54844 | | | 0.4974 | | | -1.10261 | | | 0.270198 | | protein_coding | | XP_008789913.1 | | ethylene-responsive transcription factor ERF071-like | |
| LOC103707265 | | 0.47491 | | | 1.284123 | | | 0.369832 | | | 0.711507 | | protein_coding | | XP_008789907.1 | | ethylene-responsive transcription factor ERF071-like | |
| LOC103707143 | | -0.09647 | | | 0.309417 | | | -0.31178 | | | 0.755207 | | protein_coding | | XP_008789749.1 | | ethylene-responsive transcription factor 9-like | |
| LOC103707142 | | 1.286307 | | | 0.421418 | | | 3.052329 | | | 0.002271 | | protein_coding | | XP_008789748.1 | | ethylene-responsive transcription factor 12-like | |
| LOC103707084 | | 0.305072 | | | 0.264475 | | | 1.153498 | | | 0.248706 | | protein_coding | | XP_008789665.1 | | AP2-like ethylene-responsive transcription factor AIL5 | |
| LOC103706643 | | 0.1537 | | | 0.169348 | | | 0.907599 | | | 0.36409 | | protein_coding | | XP_008789029.1; XP_008789031.1; XP_008789030.1 | | AP2-like ethylene-responsive transcription factor TOE3 | |
| LOC103706504 | | 0.67371 | | | 0.289475 | | | 2.327356 | | | 0.019946 | | protein_coding | | XP_008788844.1 | | ethylene-responsive transcription factor RAP2-1 | |
| LOC103705587 | | 0.880414 | | | 0.518234 | | | 1.698873 | | | 0.089343 | | protein_coding | | XP_008787571.1 | | ethylene-responsive transcription factor 4-like | |
| LOC103705326 | | -4.07987 | | | 1.4991 | | | -2.72155 | | | 0.006498 | | protein_coding | | XP_008787214.1 | | ethylene-responsive transcription factor ERN1-like | |
| LOC103705151 | | -1.2863 | | | 1.881519 | | | -0.68365 | | | 0.494197 | | protein_coding | | XP_008786998.1 | | ethylene-responsive transcription factor ERF018-like | |
| LOC103705139 | | 4.190783 | | | 3.351223 | | | 1.250523 | | | 0.211108 | | protein_coding | | XP_008786989.1 | | ethylene-responsive transcription factor ERF017 | |
| LOC103704965 | | -0.46621 | | | 0.703799 | | | -0.66243 | | | 0.507698 | | protein_coding | | XP_026659562.1 | | AP2-like ethylene-responsive transcription factor AIL1 | |
| LOC103704867 | | -2.15838 | | | 1.95167 | | | -1.10592 | | | 0.268763 | | protein_coding | | XP_008786563.1 | | ethylene-responsive transcription factor ERF019 | |
| LOC103704771 | | -1.22956 | | | 0.883013 | | | -1.39246 | | | 0.163784 | | protein_coding | | XP_008786418.1 | | ethylene-responsive transcription factor ERF038 | |
| LOC103704663 | | -0.45243 | | | 0.317203 | | | -1.42631 | | | 0.153778 | | protein_coding | | XP_008786271.1 | | ethylene-responsive transcription factor 2-like | |
| LOC103704662 | | -1.6943 | | | 0.523009 | | | -3.23952 | | | 0.001197 | | protein_coding | | XP_008786269.1 | | ethylene-responsive transcription factor ERF105-like | |
| LOC103704371 | | -0.52566 | | | 0.249844 | | | -2.10396 | | | 0.035382 | | protein_coding | | XP_008785849.1 | | ethylene-responsive transcription factor ERF110-like | |
| LOC103704280 | | -0.28578 | | | 2.697498 | | | -0.10594 | | | 0.915629 | | protein_coding | | XP_008785732.1 | | AP2-like ethylene-responsive transcription factor CRL5 | |
| LOC103703714 | | -5.05784 | | | 2.014473 | | | -2.51075 | | | 0.012048 | | protein_coding | | XP_008784884.2 | | ethylene-responsive transcription factor ERF084 | |
| LOC103703635 | | 0.941294 | | | 0.786305 | | | 1.19711 | | | 0.231264 | | protein_coding | | XP_008784774.1 | | ethylene-responsive transcription factor RAP2-3-like | |
| LOC103703634 | | 0.49078 | | | 0.248607 | | | 1.974121 | | | 0.048368 | | protein_coding | | XP_008784773.2 | | ethylene-responsive transcription factor ERF071-like | |
| LOC103703576 | | 1.813049 | | | 1.302264 | | | 1.392229 | | | 0.163853 | | protein_coding | | XP_008784708.1 | | ethylene-responsive transcription factor 1B-like | |
| LOC103703547 | | -0.34825 | | | 0.520035 | | | -0.66967 | | | 0.503065 | | protein_coding | | XP_017697237.1 | | AP2-like ethylene-responsive transcription factor CRL5 | |
| LOC103703325 | | -0.04205 | | | 0.114727 | | | -0.36651 | | | 0.713983 | | protein_coding | | XP_026658873.1; XP_008784363.1 | | ethylene-responsive transcription factor 1-like | |
| LOC103703306 | | -0.22498 | | | 3.659388 | | | -0.06148 | | | 0.950976 | | protein_coding | | XP_008784342.1 | | ethylene-responsive transcription factor ERF017 | |
| LOC103703252 | | 6.460151 | | | 2.337215 | | | 2.764037 | | | 0.005709 | | protein_coding | | XP_008784254.1 | | ethylene-responsive transcription factor ERF110-like | |
| LOC103703033 | | 1.661394 | | | 0.630957 | | | 2.633131 | | | 0.00846 | | protein_coding | | XP_008783954.1 | | ethylene-responsive transcription factor CRF1-like | |
| LOC103702850 | | -0.28588 | | | 0.243104 | | | -1.17596 | | | 0.239611 | | protein_coding | | XP_017697250.1 | | AP2-like ethylene-responsive transcription factor TOE3 | |
| LOC103702500 | | -2.2538 | | | 1.632768 | | | -1.38035 | | | 0.167478 | | protein_coding | | XP_008783177.1 | | ethylene-responsive transcription factor ERF023-like | |
| LOC103702455 | | 0.159497 | | | 0.165476 | | | 0.963868 | | | 0.335112 | | protein_coding | | XP_008783120.1 | | ethylene-responsive transcription factor 1-like | |
| LOC103702445 | | -0.18219 | | | 0.176206 | | | -1.03395 | | | 0.301158 | | protein_coding | | XP_008783109.1 | | ethylene-responsive transcription factor 1-like | |
| LOC103701823 | | -0.0577 | | | 0.560126 | | | -0.103 | | | 0.917959 | | protein_coding | | XP_008782235.1 | | ethylene-responsive transcription factor ERF014-like | |
| LOC103701744 | | -0.11009 | | | 0.371273 | | | -0.29652 | | | 0.766832 | | protein_coding | | XP_008782127.1 | | ethylene-responsive transcription factor RAP2-4-like | |
| LOC103701359 | | 1.92411 | | | 0.616707 | | | 3.119975 | | | 0.001809 | | protein_coding | | XP_008781621.3 | | ethylene-responsive transcription factor CRF1 | |
| LOC103701239 | | 3.539646 | | | 1.291878 | | | 2.739923 | | | 0.006145 | | protein_coding | | XP_008781454.1 | | ethylene-responsive transcription factor ABR1-like | |
| LOC103700921 | | 4.266388 | | | 3.933945 | | | 1.084506 | | | 0.27814 | | protein_coding | | XP_008781042.1 | | ethylene-responsive transcription factor ERN1-like | |
| LOC103700250 | | -0.06068 | | | 0.599596 | | | -0.1012 | | | 0.919389 | | protein_coding | | XP_008780429.1 | | ethylene-responsive transcription factor CRF4-like | |
| LOC103699420 | | 0.395289 | | | 0.282056 | | | 1.401458 | | | 0.161077 | | protein_coding | | XP_008779660.1 | | AP2-like ethylene-responsive transcription factor CRL5 | |
| LOC103699307 | | 0.829492 | | | 0.55099 | | | 1.505457 | | | 0.132207 | | protein_coding | | XP_008779564.1 | | ethylene-responsive transcription factor ERF054-like | |
| LOC103699305 | | 0.385216 | | | 0.289529 | | | 1.330492 | | | 0.183356 | | protein_coding | | XP_008779563.2 | | ethylene-responsive transcription factor 5-like | |
| LOC103699031 | | 0.459671 | | | 3.519366 | | | 0.130612 | | | 0.896082 | | protein_coding | | XP_008779295.1 | | ethylene-responsive transcription factor ERF003-like | |
| LOC103698111 | | 1.925268 | | | 0.511342 | | | 3.765126 | | | 0.000166 | | protein_coding | | XP_008778306.1 | | ethylene-responsive transcription factor 12-like | |
| LOC103697848 | | 0.306892 | | | 0.320479 | | | 0.957605 | | | 0.338262 | | protein_coding | | XP_008778008.1 | | ethylene-responsive transcription factor 12-like | |
| LOC103697556 | | -0.14783 | | | 0.298777 | | | -0.49479 | | | 0.620751 | | protein_coding | | XP_008777662.1 | | ethylene-responsive transcription factor 4-like | |
| LOC103697362 | | 0.07139 | | | 0.654346 | | | 0.109102 | | | 0.913122 | | protein_coding | | XP_008777425.1 | | AP2-like ethylene-responsive transcription factor ANT | |
| LOC103697259 | | 1.320901 | | | 2.659366 | | | 0.496698 | | | 0.619402 | | protein_coding | | XP_008777305.1 | | ethylene-responsive transcription factor ERF062-like | |
| LOC103696620 | | -0.66253 | | | 0.374332 | | | -1.7699 | | | 0.076744 | | protein_coding | | XP_026656764.1 | | ethylene-responsive transcription factor ERF011-like | |
| LOC103696378 | | 1.272897 | | | 0.858639 | | | 1.482458 | | | 0.138218 | | protein_coding | | XP_008776202.1 | | ethylene-responsive transcription factor ERF061-like | |
| LOC103696173 | | 0.160574 | | | 2.001918 | | | 0.08021 | | | 0.93607 | | protein_coding | | XP_026656596.1 | | AP2-like ethylene-responsive transcription factor AIL5 | |
| LOC103695579 | | 0.079991 | | | 0.592301 | | | 0.135051 | | | 0.892571 | | protein_coding | | XP_008775170.1 | | ethylene-responsive transcription factor TINY-like | |
| characteristics of differentially expressed ethylene-responsive transcription factors in the Fungi treated group | | | | | | | | | | | | | | | | | | |
| Gene_Symbol | log2FoldChange | | | lfcSE | | | stat | | | pvalue | | | | Type | | Protein_ID | product | |
| LOC113462365 | -0.91813 | | | 0.882434 | | | -1.04046 | | | 0.298128 | | | | protein_coding | | XP_026658220.1 | ethylene-responsive transcription factor ERF107-like | |
| LOC113461956 | -0.30951 | | | 0.813662 | | | -0.3804 | | | 0.703652 | | | | protein_coding | | XP_026657762.1 | ethylene-responsive transcription factor CRF4-like | |
| LOC103723824 | 0.067561 | | | 0.448158 | | | 0.150753 | | | 0.880171 | | | | protein_coding | | XP_008813098.1 | ethylene-responsive transcription factor ERN1 | |
| LOC103722685 | -0.01703 | | | 0.785408 | | | -0.02168 | | | 0.982702 | | | | protein_coding | | XP_008811549.2 | ethylene-responsive transcription factor 1B-like | |
| LOC103722354 | 1.461643 | | | 0.638365 | | | 2.289667 | | | 0.022041 | | | | protein_coding | | XP_017701969.1; XP_008811102.1 | ethylene-responsive transcription factor ERF113-like | |
| LOC103722008 | -0.30043 | | | 0.139252 | | | -2.15747 | | | 0.030969 | | | | protein_coding | | XP_008810643.1; XP_026666181.1 | ethylene-responsive transcription factor-like protein At4g13040 | |
| LOC103721225 | -0.61539 | | | 0.272337 | | | -2.25966 | | | 0.023843 | | | | protein_coding | | XP_026665879.1 | AP2-like ethylene-responsive transcription factor At2g41710 | |
| LOC103720343 | 4.697224 | | | 0.506793 | | | 9.268525 | | | 1.89E-20 | | | | protein_coding | | XP_008808223.1 | ethylene-responsive transcription factor ERF003-like | |
| LOC103720309 | -0.2053 | | | 0.113999 | | | -1.8009 | | | 0.071719 | | | | protein_coding | | XP_008808175.1 | ethylene-responsive transcription factor ERF060-like | |
| LOC103720224 | -0.12967 | | | 0.110209 | | | -1.17655 | | | 0.239374 | | | | protein_coding | | XP_008808053.1 | AP2-like ethylene-responsive transcription factor AIL1 | |
| LOC103719993 | 0.033532 | | | 0.111757 | | | 0.300045 | | | 0.764143 | | | | protein_coding | | XP_008807712.1; XP_008807713.1 | AP2-like ethylene-responsive transcription factor BBM1 | |
| LOC103719783 | 0.043676 | | | 0.624023 | | | 0.069992 | | | 0.9442 | | | | protein_coding | | XP_008807399.1 | ethylene-responsive transcription factor ERF071-like | |
| LOC103719666 | 0.746957 | | | 1.081018 | | | 0.690976 | | | 0.489581 | | | | protein_coding | | XP_008807234.1 | ethylene-responsive transcription factor ERF023-like | |
| LOC103719441 | 0.728501 | | | 1.268188 | | | 0.574443 | | | 0.565668 | | | | protein_coding | | XP_008806912.1 | ethylene-responsive transcription factor 1B-like | |
| LOC103719025 | 0.909399 | | | 0.618335 | | | 1.470722 | | | 0.141366 | | | | protein_coding | | XP_008806298.1 | ethylene-responsive transcription factor ERF003-like | |
| LOC103717663 | -0.85721 | | | 0.559249 | | | -1.53279 | | | 0.125327 | | | | protein_coding | | XP_008804361.1 | ethylene-responsive transcription factor ERN1-like | |
| LOC103717533 | 1.478672 | | | 0.988699 | | | 1.495574 | | | 0.134765 | | | | protein_coding | | XP_008804192.2 | AP2-like ethylene-responsive transcription factor BBM2 | |
| LOC103717275 | 0.135836 | | | 0.415719 | | | 0.32675 | | | 0.743857 | | | | protein_coding | | XP_008803818.3 | ethylene-responsive transcription factor ERF034-like | |
| LOC103717228 | -0.08864 | | | 0.146964 | | | -0.60315 | | | 0.546408 | | | | protein_coding | | XP_008803760.1 | ethylene-responsive transcription factor RAP2-4-like | |
| LOC103717110 | -0.54029 | | | 0.24276 | | | -2.22563 | | | 0.026039 | | | | protein_coding | | XP_008803593.1 | AP2-like ethylene-responsive transcription factor AIL5 | |
| LOC103717086 | 0.478492 | | | 0.268941 | | | 1.779172 | | | 0.075212 | | | | protein_coding | | XP_008803555.1 | ethylene-responsive transcription factor CRF1-like | |
| LOC103717048 | -0.25233 | | | 0.239672 | | | -1.0528 | | | 0.292433 | | | | protein_coding | | XP_026664229.1; XP_008803499.1; XP_008803501.1; XP_008803500.1 | ethylene-responsive transcription factor-like protein At4g13040 | |
| LOC103717044 | -0.28106 | | | 0.14356 | | | -1.95778 | | | 0.050256 | | | | protein_coding | | XP_008803496.1 | ethylene-responsive transcription factor 3-like | |
| LOC103716571 | -0.88958 | | | 0.562027 | | | -1.58281 | | | 0.113464 | | | | protein_coding | | XP_008802835.1 | ethylene-responsive transcription factor 11-like | |
| LOC103716427 | -0.0553 | | | 0.185124 | | | -0.29874 | | | 0.765136 | | | | protein_coding | | XP_008802631.1; XP_008802632.1 | AP2-like ethylene-responsive transcription factor At2g41710 | |
| LOC103716011 | -2.02179 | | | 0.402983 | | | -5.01706 | | | 5.25E-07 | | | | protein_coding | | XP_008802060.1 | ethylene-responsive transcription factor ERF014-like | |
| LOC103715808 | -1.86943 | | | 0.424188 | | | -4.40707 | | | 1.05E-05 | | | | protein_coding | | XP_008801788.1 | ethylene-responsive transcription factor ERF105-like | |
| LOC103713218 | -1.21158 | | | 0.275767 | | | -4.3935 | | | 1.12E-05 | | | | protein_coding | | XP_008798289.3 | ethylene-responsive transcription factor 2-like | |
| LOC103712528 | -1.52861 | | | 0.733708 | | | -2.0834 | | | 0.037214 | | | | protein_coding | | XP_008797298.1 | ethylene-responsive transcription factor ERF014-like | |
| LOC103712373 | 0.901091 | | | 0.942932 | | | 0.955626 | | | 0.339261 | | | | protein_coding | | XP_008797100.1 | ethylene-responsive transcription factor LEP-like | |
| LOC103711902 | 0.241725 | | | 0.77607 | | | 0.311474 | | | 0.75544 | | | | protein_coding | | XP_008796443.1 | ethylene-responsive transcription factor 3-like | |
| LOC103711691 | 2.412742 | | | 0.797007 | | | 3.027254 | | | 0.002468 | | | | protein_coding | | XP_017699428.1 | ethylene-responsive transcription factor ERF039-like | |
| LOC103711088 | -2.56455 | | | 0.660549 | | | -3.88245 | | | 0.000103 | | | | protein_coding | | XP_008795312.1 | ethylene-responsive transcription factor ERF038-like | |
| LOC103710089 | 1.096849 | | | 1.166908 | | | 0.939962 | | | 0.347237 | | | | protein_coding | | XP_008793924.1; XP_008793923.1; XP_026661617.1 | ethylene-responsive transcription factor ERF115-like | |
| LOC103709666 | 1.707255 | | | 0.320678 | | | 5.323899 | | | 1.02E-07 | | | | protein_coding | | XP_008793348.3 | AP2-like ethylene-responsive transcription factor AIL5 | |
| LOC103709065 | 0.398282 | | | 1.563518 | | | 0.254734 | | | 0.798928 | | | | protein_coding | | XP_026661158.1 | ethylene-responsive transcription factor WRI1-like | |
| LOC103708547 | 0.277949 | | | 0.416578 | | | 0.667219 | | | 0.504632 | | | | protein_coding | | XP_008791735.1 | AP2-like ethylene-responsive transcription factor AIL1 | |
| LOC103708042 | 0.152245 | | | 0.195605 | | | 0.778332 | | | 0.436373 | | | | protein_coding | | XP_008791015.1 | ethylene-responsive transcription factor ERF113-like | |
| LOC103707892 | -0.39184 | | | 0.160984 | | | -2.43405 | | | 0.014931 | | | | protein_coding | | XP_017698527.1 | ethylene-responsive transcription factor RAP2-13-like | |
| LOC103707419 | 1.506258 | | | 1.061563 | | | 1.418906 | | | 0.155926 | | | | protein_coding | | XP_008790117.1 | AP2-like ethylene-responsive transcription factor At1g16060 | |
| LOC103707265 | 0.326665 | | | 0.801081 | | | 0.40778 | | | 0.683435 | | | | protein_coding | | XP_008789907.1 | ethylene-responsive transcription factor ERF071-like | |
| LOC103707142 | -0.40276 | | | 0.449215 | | | -0.89658 | | | 0.369941 | | | | protein_coding | | XP_008789748.1 | ethylene-responsive transcription factor 12-like | |
| LOC103707084 | -0.17214 | | | 0.219471 | | | -0.78435 | | | 0.432838 | | | | protein_coding | | XP_008789665.1 | AP2-like ethylene-responsive transcription factor AIL5 | |
| LOC103706504 | -0.33811 | | | 0.28171 | | | -1.20019 | | | 0.230064 | | | | protein_coding | | XP_008788844.1 | ethylene-responsive transcription factor RAP2-1 | |
| LOC103705326 | -0.77412 | | | 0.856709 | | | -0.9036 | | | 0.366207 | | | | protein_coding | | XP_008787214.1 | ethylene-responsive transcription factor ERN1-like | |
| LOC103704771 | -2.52267 | | | 1.055098 | | | -2.39093 | | | 0.016806 | | | | protein_coding | | XP_008786418.1 | ethylene-responsive transcription factor ERF038 | |
| LOC103704371 | -1.99809 | | | 0.222678 | | | -8.97302 | | | 2.88E-19 | | | | protein_coding | | XP_008785849.1 | ethylene-responsive transcription factor ERF110-like | |
| LOC103703714 | -0.9399 | | | 1.245155 | | | -0.75485 | | | 0.450341 | | | | protein_coding | | XP_008784884.2 | ethylene-responsive transcription factor ERF084 | |
| LOC103703635 | -0.32015 | | | 0.801131 | | | -0.39962 | | | 0.689437 | | | | protein_coding | | XP_008784774.1 | ethylene-responsive transcription factor RAP2-3-like | |
| LOC103703547 | -0.80213 | | | 0.551537 | | | -1.45436 | | | 0.145847 | | | | protein_coding | | XP_017697237.1 | AP2-like ethylene-responsive transcription factor CRL5 | |
| LOC103703325 | -0.04297 | | | 0.08555 | | | -0.50226 | | | 0.615485 | | | | protein_coding | | XP_026658873.1; XP_008784363.1 | ethylene-responsive transcription factor 1-like | |
| LOC103703252 | 7.996615 | | | 1.303886 | | | 6.132908 | | | 8.63E-10 | | | | protein_coding | | XP_008784254.1 | ethylene-responsive transcription factor ERF110-like | |
| LOC103702850 | 0.146025 | | | 0.139983 | | | 1.043157 | | | 0.296876 | | | | protein_coding | | XP_017697250.1 | AP2-like ethylene-responsive transcription factor TOE3 | |
| LOC103702500 | 0.445711 | | | 1.081918 | | | 0.411964 | | | 0.680366 | | | | protein_coding | | XP_008783177.1 | ethylene-responsive transcription factor ERF023-like | |
| LOC103702455 | 0.117455 | | | 0.117258 | | | 1.001673 | | | 0.316502 | | | | protein_coding | | XP_008783120.1 | ethylene-responsive transcription factor 1-like | |
| LOC103701823 | 0.601575 | | | 0.405721 | | | 1.482731 | | | 0.138146 | | | | protein_coding | | XP_008782235.1 | ethylene-responsive transcription factor ERF014-like | |
| LOC103701359 | 1.10549 | | | 0.551989 | | | 2.00274 | | | 0.045205 | | | | protein_coding | | XP_008781621.3 | ethylene-responsive transcription factor CRF1 | |
| LOC103701239 | -0.9732 | | | 1.288486 | | | -0.7553 | | | 0.450068 | | | | protein_coding | | XP_008781454.1 | ethylene-responsive transcription factor ABR1-like | |
| LOC103700250 | -0.11976 | | | 0.548517 | | | -0.21834 | | | 0.827166 | | | | protein_coding | | XP_008780429.1 | ethylene-responsive transcription factor CRF4-like | |
| LOC103699420 | 0.212654 | | | 0.257033 | | | 0.82734 | | | 0.408045 | | | | protein_coding | | XP_008779660.1 | AP2-like ethylene-responsive transcription factor CRL5 | |
| LOC103699305 | -0.18894 | | | 0.244633 | | | -0.77236 | | | 0.439904 | | | | protein_coding | | XP_008779563.2 | ethylene-responsive transcription factor 5-like | |
| LOC103698111 | -0.09018 | | | 0.339638 | | | -0.26553 | | | 0.7906 | | | | protein_coding | | XP_008778306.1 | ethylene-responsive transcription factor 12-like | |
| LOC103697848 | 0.673876 | | | 0.297271 | | | 2.266878 | | | 0.023398 | | | | protein_coding | | XP_008778008.1 | ethylene-responsive transcription factor 12-like | |
| LOC103697556 | -0.30947 | | | 0.20712 | | | -1.49416 | | | 0.135133 | | | | protein_coding | | XP_008777662.1 | ethylene-responsive transcription factor 4-like | |
| LOC103697362 | -2.01604 | | | 0.825506 | | | -2.44219 | | | 0.014598 | | | | protein_coding | | XP_008777425.1 | AP2-like ethylene-responsive transcription factor ANT | |
| LOC103696620 | -0.27873 | | | 0.313793 | | | -0.88825 | | | 0.374405 | | | | protein_coding | | XP_026656764.1 | ethylene-responsive transcription factor ERF011-like | |
| LOC103696378 | 0.318703 | | | 0.706454 | | | 0.451131 | | | 0.651895 | | | | protein_coding | | XP_008776202.1 | ethylene-responsive transcription factor ERF061-like | |
| LOC103695579 | 0.91451 | | | 0.399515 | | | 2.28905 | | | 0.022076 | | | | protein_coding | | XP_008775170.1 | ethylene-responsive transcription factor TINY-like | |
| Characteristics of differentially expressed ethylene-responsive transcription factors in the Fungi+Salt treated group | | | | | | | | | | | | | | | | | | |
| Gene_Symbol | | | log2FoldChange | | | lfcSE | | | pvalue | | | padj | | Type | | Protein_ID | product |  |
| LOC113462365 | | | 0.451972 | | | 0.734289 | | | 0.538209 | | | 0.697915 | | protein_conding | | XP_026658220.1 | ethylene-responsive transcription factor ERF107-like | |
| LOC113461956 | | | 0.071253 | | | 0.863323 | | | 0.934223 | | | 0.965834 | | protein_conding | | XP_026657762.1 | ethylene-responsive transcription factor CRF4-like | |
| LOC108510684 | | | -0.11537 | | | 3.153427 | | | 0.970816 | | | 0.983616 | | protein_conding | | XP_017697344.1 | ethylene-responsive transcription factor ERF017-like | |
| LOC103724191 | | | -0.92651 | | | 0.154595 | | | 2.06E-09 | | | 2.95E-08 | | protein_conding | | XP_008813598.1 | ethylene-responsive transcription factor 2 | |
| LOC103723824 | | | 0.208104 | | | 0.476138 | | | 0.662063 | | | 0.795237 | | protein_conding | | XP_008813098.1 | ethylene-responsive transcription factor ERN1 | |
| LOC103722685 | | | -1.44355 | | | 0.953255 | | | 0.129941 | | | 0.258662 | | protein_conding | | XP_008811549.2 | ethylene-responsive transcription factor 1B-like | |
| LOC103722581 | | | -0.8377 | | | 2.923565 | | | 0.77447 | | | 0.870437 | | protein_conding | | XP_008811407.1 | ethylene-responsive transcription factor ERF071-like | |
| LOC103722354 | | | 2.286149 | | | 0.33843 | | | 1.43E-11 | | | 2.73E-10 | | protein_conding | | XP_017701969.1; XP_008811102.1 | ethylene-responsive transcription factor ERF113-like | |
| LOC103722021 | | | -0.22297 | | | 2.973472 | | | 0.940225 | | | 0.968605 | | protein_conding | | XP_008810659.1 | ethylene-responsive transcription factor TINY-like | |
| LOC103722008 | | | 0.23171 | | | 0.124418 | | | 0.062554 | | | 0.146777 | | protein_conding | | XP_008810643.1; XP_026666181.1 | ethylene-responsive transcription factor-like protein At4g13040 | |
| LOC103721682 | | | 2.329393 | | | 2.006768 | | | 0.245736 | | | 0.411806 | | protein_conding | | XP_008810207.1 | ethylene-responsive transcription factor ERF071-like | |
| LOC103721225 | | | 0.089036 | | | 0.210957 | | | 0.672982 | | | 0.803576 | | protein_conding | | XP_026665879.1 | AP2-like ethylene-responsive transcription factor At2g41710 | |
| LOC103721201 | | | 0.726643 | | | 1.319177 | | | 0.58175 | | | 0.734398 | | protein_conding | | XP_008809528.1 | ethylene-responsive transcription factor LEP-like | |
| LOC103721161 | | | 0.209378 | | | 0.333548 | | | 0.530181 | | | 0.69127 | | protein_conding | | XP_008809462.1 | ethylene-responsive transcription factor 2-like | |
| LOC103720343 | | | -1.34436 | | | 1.427587 | | | 0.346347 | | | 0.523005 | | protein_conding | | XP_008808223.1 | ethylene-responsive transcription factor ERF003-like | |
| LOC103720309 | | | 0.26349 | | | 0.09217 | | | 0.004253 | | | 0.01623 | | protein_conding | | XP_008808175.1 | ethylene-responsive transcription factor ERF060-like | |
| LOC103720224 | | | 0.192339 | | | 0.098729 | | | 0.051397 | | | 0.126025 | | protein_conding | | XP_008808053.1 | AP2-like ethylene-responsive transcription factor AIL1 | |
| LOC103719993 | | | 0.413554 | | | 0.094784 | | | 1.28E-05 | | | 9.58E-05 | | protein_conding | | XP_008807712.1; XP_008807713.1 | AP2-like ethylene-responsive transcription factor BBM1 | |
| LOC103719783 | | | -0.68422 | | | 0.713505 | | | 0.337581 | | | 0.513773 | | protein_conding | | XP_008807399.1 | ethylene-responsive transcription factor ERF071-like | |
| LOC103719666 | | | 0.615917 | | | 1.15727 | | | 0.594577 | | | 0.745181 | | protein_conding | | XP_008807234.1 | ethylene-responsive transcription factor ERF023-like | |
| LOC103719442 | | | 4.990456 | | | 2.084415 | | | 0.016658 | | | 0.050624 | | protein_conding | | XP_008806913.1 | ethylene-responsive transcription factor ERF096-like | |
| LOC103719441 | | | 0.93772 | | | 0.965243 | | | 0.331306 | | | 0.506884 | | protein_conding | | XP_008806912.1 | ethylene-responsive transcription factor 1B-like | |
| LOC103719025 | | | -3.96126 | | | 1.176944 | | | 0.000763 | | | 0.003703 | | protein_conding | | XP_008806298.1 | ethylene-responsive transcription factor ERF003-like | |
| LOC103718991 | | | 0.35097 | | | 0.12665 | | | 0.005585 | | | 0.020459 | | protein_conding | | XP_026665033.1 | ethylene-responsive transcription factor ERF039-like | |
| LOC103717663 | | | -2.74937 | | | 0.822795 | | | 0.000833 | | | 0.003994 | | protein_conding | | XP_008804361.1 | ethylene-responsive transcription factor ERN1-like | |
| LOC103717533 | | | -2.17061 | | | 1.696139 | | | 0.200638 | | | 0.356234 | | protein_conding | | XP_008804192.2 | AP2-like ethylene-responsive transcription factor BBM2 | |
| LOC103717275 | | | -1.61953 | | | 0.441534 | | | 0.000244 | | | 0.001348 | | protein_conding | | XP_008803818.3 | ethylene-responsive transcription factor ERF034-like | |
| LOC103717228 | | | 1.116118 | | | 0.110539 | | | 5.69E-24 | | | 3.25E-22 | | protein_conding | | XP_008803760.1 | ethylene-responsive transcription factor RAP2-4-like | |
| LOC103717086 | | | 0.431595 | | | 0.250436 | | | 0.084821 | | | 0.186941 | | protein_conding | | XP_008803555.1 | ethylene-responsive transcription factor CRF1-like | |
| LOC103717048 | | | -0.27909 | | | 0.243199 | | | 0.25115 | | | 0.417904 | | protein_conding | | XP_026664229.1; XP_008803499.1; XP_008803501.1; XP_008803500.1 | ethylene-responsive transcription factor-like protein At4g13040 | |
| LOC103717044 | | | 1.330496 | | | 0.110363 | | | 1.81E-33 | | | 1.87E-31 | | protein_conding | | XP_008803496.1 | ethylene-responsive transcription factor 3-like | |
| LOC103716571 | | | 1.30342 | | | 0.40567 | | | 0.001314 | | | 0.00591 | | protein_conding | | XP_008802835.1 | ethylene-responsive transcription factor 11-like | |
| LOC103716427 | | | 0.386459 | | | 0.177592 | | | 0.029547 | | | 0.080836 | | protein_conding | | XP_008802631.1; XP_008802632.1 | AP2-like ethylene-responsive transcription factor At2g41710 | |
| LOC103716011 | | | -5.62281 | | | 0.603869 | | | 1.26E-20 | | | 5.62E-19 | | protein_conding | | XP_008802060.1 | ethylene-responsive transcription factor ERF014-like | |
| LOC103715808 | | | -0.94571 | | | 0.354869 | | | 0.0077 | | | 0.026771 | | protein_conding | | XP_008801788.1 | ethylene-responsive transcription factor ERF105-like | |
| LOC103715562 | | | -2.87353 | | | 3.958554 | | | 0.467898 | | | 0.638399 | | protein_conding | | XP_008801457.1 | ethylene-responsive transcription factor ERF015 | |
| LOC103714855 | | | -0.62479 | | | 3.361999 | | | 0.85257 | | | 0.920143 | | protein_conding | | XP_008800510.2 | ethylene-responsive transcription factor ERF016-like | |
| LOC103714826 | | | 0.223864 | | | 0.090067 | | | 0.012936 | | | 0.041099 | | protein_conding | | XP_008800472.1; XP_008800471.1 | ethylene-responsive transcription factor 1-like | |
| LOC103713952 | | | 0.914155 | | | 0.105274 | | | 3.83E-18 | | | 1.35E-16 | | protein_conding | | XP_008799235.1 | ethylene-responsive transcription factor CRF2-like | |
| LOC103713218 | | | 1.254672 | | | 0.215327 | | | 5.65E-09 | | | 7.63E-08 | | protein_conding | | XP_008798289.3 | ethylene-responsive transcription factor 2-like | |
| LOC103712913 | | | 0.338955 | | | 0.104134 | | | 0.001134 | | | 0.005189 | | protein_conding | | XP_008797843.1 | ethylene-responsive transcription factor CRF2 | |
| LOC103712588 | | | -0.8799 | | | 0.108827 | | | 6.2E-16 | | | 1.82E-14 | | protein_conding | | XP_008797374.1 | ethylene-responsive transcription factor 1-like | |
| LOC103712528 | | | -5.09519 | | | 1.294652 | | | 0.000083 | | | 0.000512 | | protein_conding | | XP_008797298.1 | ethylene-responsive transcription factor ERF014-like | |
| LOC103712373 | | | -1.83713 | | | 1.495136 | | | 0.219169 | | | 0.379732 | | protein_conding | | XP_008797100.1 | ethylene-responsive transcription factor LEP-like | |
| LOC103711902 | | | 0.618032 | | | 0.648511 | | | 0.340589 | | | 0.51717 | | protein_conding | | XP_008796443.1 | ethylene-responsive transcription factor 3-like | |
| LOC103711691 | | | -2.1118 | | | 1.908746 | | | 0.268561 | | | 0.438239 | | protein_conding | | XP_017699428.1 | ethylene-responsive transcription factor ERF039-like | |
| LOC103711505 | | | -0.64727 | | | 0.223086 | | | 0.003715 | | | 0.014483 | | protein_conding | | XP_008795894.1; XP_026662179.1; XP_008795895.1 | AP2-like ethylene-responsive transcription factor TOE3 | |
| LOC103711503 | | | 0.87034 | | | 0.165977 | | | 1.57E-07 | | | 1.68E-06 | | protein_conding | | XP_017699377.1 | ethylene-responsive transcription factor ERF011-like | |
| LOC103711129 | | | -1.6609 | | | 2.432406 | | | 0.49472 | | | 0.660613 | | protein_conding | | XP_008795369.1 | ethylene-responsive transcription factor ERF027 | |
| LOC103711088 | | | -7.37784 | | | 1.31114 | | | 1.83E-08 | | | 2.29E-07 | | protein_conding | | XP_008795312.1 | ethylene-responsive transcription factor ERF038-like | |
| LOC103710089 | | | 1.818866 | | | 1.031053 | | | 0.077718 | | | 0.174294 | | protein_conding | | XP_008793924.1; XP_008793923.1; XP_026661617.1 | ethylene-responsive transcription factor ERF115-like | |
| LOC103709666 | | | 0.029963 | | | 0.323164 | | | 0.926127 | | | 0.961185 | | protein_conding | | XP_008793348.3 | AP2-like ethylene-responsive transcription factor AIL5 | |
| LOC103709326 | | | -6.41016 | | | 1.559292 | | | 3.94E-05 | | | 0.000265 | | protein_conding | | XP_008792845.1 | ethylene-responsive transcription factor ERF017-like | |
| LOC103709065 | | | -1.35548 | | | 1.811337 | | | 0.454261 | | | 0.626632 | | protein_conding | | XP_026661158.1 | ethylene-responsive transcription factor WRI1-like | |
| LOC103708748 | | | -3.67024 | | | 2.868473 | | | 0.200718 | | | 0.356327 | | protein_conding | | XP_008792041.1 | ethylene-responsive transcription factor WIN1-like | |
| LOC103708547 | | | -1.92445 | | | 0.523505 | | | 0.000237 | | | 0.00131 | | protein_conding | | XP_008791735.1 | AP2-like ethylene-responsive transcription factor AIL1 | |
| LOC103708353 | | | -0.10995 | | | 1.319844 | | | 0.933606 | | | 0.965444 | | protein_conding | | XP_008791463.1 | ethylene-responsive transcription factor ERF069-like | |
| LOC103708330 | | | -4.09348 | | | 2.622772 | | | 0.118584 | | | 0.241928 | | protein_conding | | XP_008791428.1 | ethylene-responsive transcription factor ERF061 | |
| LOC103708042 | | | 0.895791 | | | 0.112156 | | | 1.38E-15 | | | 3.95E-14 | | protein_conding | | XP_008791015.1 | ethylene-responsive transcription factor ERF113-like | |
| LOC103707892 | | | 0.036315 | | | 0.152481 | | | 0.811754 | | | 0.894156 | | protein_conding | | XP_017698527.1 | ethylene-responsive transcription factor RAP2-13-like | |
| LOC103707419 | | | -0.10446 | | | 1.630597 | | | 0.948918 | | | 0.973181 | | protein_conding | | XP_008790117.1 | AP2-like ethylene-responsive transcription factor At1g16060 | |
| LOC103707269 | | | -1.05945 | | | 0.350215 | | | 0.002485 | | | 0.010236 | | protein_conding | | XP_008789913.1 | ethylene-responsive transcription factor ERF071-like | |
| LOC103707265 | | | -1.7695 | | | 1.219361 | | | 0.146733 | | | 0.283108 | | protein_conding | | XP_008789907.1 | ethylene-responsive transcription factor ERF071-like | |
| LOC103707143 | | | -1.12701 | | | 0.212456 | | | 1.13E-07 | | | 1.23E-06 | | protein_conding | | XP_008789749.1 | ethylene-responsive transcription factor 9-like | |
| LOC103707142 | | | -0.32499 | | | 0.453837 | | | 0.473937 | | | 0.643236 | | protein_conding | | XP_008789748.1 | ethylene-responsive transcription factor 12-like | |
| LOC103707084 | | | -2.29924 | | | 0.308482 | | | 9.1E-14 | | | 2.17E-12 | | protein_conding | | XP_008789665.1 | AP2-like ethylene-responsive transcription factor AIL5 | |
| LOC103706643 | | | -0.10648 | | | 0.090015 | | | 0.236831 | | | 0.401232 | | protein_conding | | XP_008789029.1; XP_008789031.1; XP_008789030.1 | AP2-like ethylene-responsive transcription factor TOE3 | |
| LOC103706504 | | | 1.301441 | | | 0.211419 | | | 7.47E-10 | | | 1.14E-08 | | protein_conding | | XP_008788844.1 | ethylene-responsive transcription factor RAP2-1 | |
| LOC103705587 | | | -0.04367 | | | 0.202274 | | | 0.829077 | | | 0.905497 | | protein_conding | | XP_008787571.1 | ethylene-responsive transcription factor 4-like | |
| LOC103705326 | | | -0.25843 | | | 0.710824 | | | 0.716187 | | | 0.83119 | | protein_conding | | XP_008787214.1 | ethylene-responsive transcription factor ERN1-like | |
| LOC103705151 | | | -1.68299 | | | 1.48195 | | | 0.2561 | | | 0.42356 | | protein_conding | | XP_008786998.1 | ethylene-responsive transcription factor ERF018-like | |
| LOC103705139 | | | 3.789442 | | | 3.007319 | | | 0.207643 | | | 0.365361 | | protein_conding | | XP_008786989.1 | ethylene-responsive transcription factor ERF017 | |
| LOC103704965 | | | -3.47905 | | | 0.603715 | | | 8.28E-09 | | | 1.1E-07 | | protein_conding | | XP_026659562.1 | AP2-like ethylene-responsive transcription factor AIL1 | |
| LOC103704867 | | | -1.99664 | | | 2.043682 | | | 0.328577 | | | 0.504151 | | protein_conding | | XP_008786563.1 | ethylene-responsive transcription factor ERF019 | |
| LOC103704771 | | | -2.23976 | | | 1.023001 | | | 0.028567 | | | 0.078603 | | protein_conding | | XP_008786418.1 | ethylene-responsive transcription factor ERF038 | |
| LOC103704663 | | | -0.02321 | | | 0.22002 | | | 0.915973 | | | 0.955356 | | protein_conding | | XP_008786271.1 | ethylene-responsive transcription factor 2-like | |
| LOC103704662 | | | 0.075456 | | | 0.357574 | | | 0.832869 | | | 0.907739 | | protein_conding | | XP_008786269.1 | ethylene-responsive transcription factor ERF105-like | |
| LOC103704371 | | | 0.363856 | | | 0.180172 | | | 0.043436 | | | 0.110146 | | protein_conding | | XP_008785849.1 | ethylene-responsive transcription factor ERF110-like | |
| LOC103704280 | | | -3.35532 | | | 3.085333 | | | 0.276813 | | | 0.44665 | | protein_conding | | XP_008785732.1 | AP2-like ethylene-responsive transcription factor CRL5 | |
| LOC103703714 | | | -0.81671 | | | 1.094773 | | | 0.455662 | | | 0.627761 | | protein_conding | | XP_008784884.2 | ethylene-responsive transcription factor ERF084 | |
| LOC103703635 | | | 0.686847 | | | 0.70074 | | | 0.327 | | | 0.502356 | | protein_conding | | XP_008784774.1 | ethylene-responsive transcription factor RAP2-3-like | |
| LOC103703634 | | | 0.321 | | | 0.157572 | | | 0.041634 | | | 0.106534 | | protein_conding | | XP_008784773.2 | ethylene-responsive transcription factor ERF071-like | |
| LOC103703576 | | | 0.325884 | | | 1.343954 | | | 0.808407 | | | 0.89184 | | protein_conding | | XP_008784708.1 | ethylene-responsive transcription factor 1B-like | |
| LOC103703547 | | | -2.92246 | | | 0.618978 | | | 2.34E-06 | | | 2.03E-05 | | protein_conding | | XP_017697237.1 | AP2-like ethylene-responsive transcription factor CRL5 | |
| LOC103703325 | | | -0.21421 | | | 0.07001 | | | 0.002216 | | | 0.009289 | | protein_conding | | XP_026658873.1; XP_008784363.1 | ethylene-responsive transcription factor 1-like | |
| LOC103703306 | | | -2.87353 | | | 3.958554 | | | 0.467898 | | | 0.638399 | | protein_conding | | XP_008784342.1 | ethylene-responsive transcription factor ERF017 | |
| LOC103703252 | | | 6.152023 | | | 1.5952 | | | 0.000115 | | | 0.000689 | | protein_conding | | XP_008784254.1 | ethylene-responsive transcription factor ERF110-like | |
| LOC103703033 | | | 0.654882 | | | 0.690707 | | | 0.343062 | | | 0.51969 | | protein_conding | | XP_008783954.1 | ethylene-responsive transcription factor CRF1-like | |
| LOC103702850 | | | -0.13997 | | | 0.135346 | | | 0.30106 | | | 0.473408 | | protein_conding | | XP_017697250.1 | AP2-like ethylene-responsive transcription factor TOE3 | |
| LOC103702500 | | | -5.10533 | | | 1.731314 | | | 0.00319 | | | 0.012685 | | protein_conding | | XP_008783177.1 | ethylene-responsive transcription factor ERF023-like | |
| LOC103702455 | | | -0.7673 | | | 0.106236 | | | 5.1E-13 | | | 1.14E-11 | | protein_conding | | XP_008783120.1 | ethylene-responsive transcription factor 1-like | |
| LOC103702445 | | | -0.07676 | | | 0.068553 | | | 0.262812 | | | 0.431419 | | protein_conding | | XP_008783109.1 | ethylene-responsive transcription factor 1-like | |
| LOC103701823 | | | -5.35352 | | | 1.034179 | | | 2.26E-07 | | | 2.35E-06 | | protein_conding | | XP_008782235.1 | ethylene-responsive transcription factor ERF014-like | |
| LOC103701744 | | | 0.235038 | | | 0.141337 | | | 0.09632 | | | 0.206364 | | protein_conding | | XP_008782127.1 | ethylene-responsive transcription factor RAP2-4-like | |
| LOC103701359 | | | 1.196891 | | | 0.48177 | | | 0.012978 | | | 0.041209 | | protein_conding | | XP_008781621.3 | ethylene-responsive transcription factor CRF1 | |
| LOC103701239 | | | 5.189127 | | | 0.428775 | | | 1.03E-33 | | | 1.08E-31 | | protein_conding | | XP_008781454.1 | ethylene-responsive transcription factor ABR1-like | |
| LOC103701013 | | | 3.76212 | | | 3.713878 | | | 0.311065 | | | 0.483935 | | protein_conding | | XP_008781163.2 | AP2-like ethylene-responsive transcription factor AIL7 | |
| LOC103700250 | | | 0.052222 | | | 0.544919 | | | 0.923652 | | | 0.959893 | | protein_conding | | XP_008780429.1 | ethylene-responsive transcription factor CRF4-like | |
| LOC103699420 | | | 0.877557 | | | 0.230075 | | | 0.000137 | | | 0.000801 | | protein_conding | | XP_008779660.1 | AP2-like ethylene-responsive transcription factor CRL5 | |
| LOC103699307 | | | 0.728124 | | | 0.242361 | | | 0.002662 | | | 0.010844 | | protein_conding | | XP_008779564.1 | ethylene-responsive transcription factor ERF054-like | |
| LOC103699305 | | | -0.01314 | | | 0.211386 | | | 0.950421 | | | 0.973949 | | protein_conding | | XP_008779563.2 | ethylene-responsive transcription factor 5-like | |
| LOC103699031 | | | -0.94578 | | | 3.230706 | | | 0.769716 | | | 0.867229 | | protein_conding | | XP_008779295.1 | ethylene-responsive transcription factor ERF003-like | |
| LOC103698149 | | | 3.529844 | | | 3.202632 | | | 0.270388 | | | 0.440268 | | protein_conding | | XP_008778360.1 | ethylene-responsive transcription factor ERF020-like | |
| LOC103698111 | | | 1.608032 | | | 0.252138 | | | 1.8E-10 | | | 3E-09 | | protein_conding | | XP_008778306.1 | ethylene-responsive transcription factor 12-like | |
| LOC103697848 | | | 0.330602 | | | 0.295185 | | | 0.262721 | | | 0.43137 | | protein_conding | | XP_008778008.1 | ethylene-responsive transcription factor 12-like | |
| LOC103697556 | | | -0.05055 | | | 0.196037 | | | 0.7965 | | | 0.884407 | | protein_conding | | XP_008777662.1 | ethylene-responsive transcription factor 4-like | |
| LOC103697362 | | | -1.25121 | | | 0.754612 | | | 0.0973 | | | 0.207909 | | protein_conding | | XP_008777425.1 | AP2-like ethylene-responsive transcription factor ANT | |
| LOC103696620 | | | 0.522361 | | | 0.268564 | | | 0.051773 | | | 0.126754 | | protein_conding | | XP_026656764.1 | ethylene-responsive transcription factor ERF011-like | |
| LOC103696378 | | | 2.197234 | | | 0.479188 | | | 4.53E-06 | | | 3.71E-05 | | protein_conding | | XP_008776202.1 | ethylene-responsive transcription factor ERF061-like | |
| LOC103696173 | | | -2.98702 | | | 2.386695 | | | 0.210742 | | | 0.368941 | | protein_conding | | XP_026656596.1 | AP2-like ethylene-responsive transcription factor AIL5 | |
| LOC103695579 | | | -1.47281 | | | 0.602419 | | | 0.014492 | | | 0.045066 | | protein_conding | | XP_008775170.1 | ethylene-responsive transcription factor TINY-like | |

Table S7: WRKY transcrips factors DEGs in the date palm treatment groups

| Characteristics of differentially expressed WRKY transcription factors in the Fungi treated group | | | | | | | | | | | | | |
| --- | --- | --- | --- | --- | --- | --- | --- | --- | --- | --- | --- | --- | --- |
| Gene_Symbol | log2FoldChange | lfcSE | pvalue | | Type | | | Protein_ID | | | product | | |
|  |  |  |  | |  | | |  | | |  | | |
| LOC108511557 | 0.403964 | 0.414096 | 0.329296 | | protein_coding | | | XP_026663206.1 | | | WRKY transcription factor WRKY24-like | | |
| LOC103724141 | -0.29476 | 0.171903 | 0.086407 | | protein_coding | | | XP_008813530.2 | | | probable WRKY transcription factor 72 | | |
| LOC103723396 | -0.03035 | 0.384551 | 0.937099 | | protein_coding | | | XP_008812523.1 | | | probable WRKY transcription factor 14 | | |
| LOC103721327 | -1.04685 | 0.209046 | 5.51E-07 | | protein_coding | | | XP_017701724.1; XP_017701728.1 | | | probable WRKY transcription factor 72 | | |
| LOC103721211 | -0.18208 | 0.2724 | 0.503849 | | protein_coding | | | XP_008809548.1 | | | probable WRKY transcription factor 75 | | |
| LOC103720998 | -0.63074 | 0.183767 | 0.000599 | | protein_coding | | | XP_008809222.1 | | | probable WRKY transcription factor 14 | | |
| LOC103720653 | 0.725866 | 0.459592 | 0.114251 | | protein_coding | | | XP_008808698.1 | | | probable WRKY transcription factor 13 | | |
| LOC103720433 | 2.284939 | 0.603047 | 0.000151 | | protein_coding | | | XP_026665098.1 | | | probable WRKY transcription factor 72 | | |
| LOC103720275 | 0.667579 | 0.812053 | 0.411027 | | protein_coding | | | XP_008808114.1 | | | probable WRKY transcription factor 75 | | |
| LOC103718944 | -0.15028 | 0.098751 | 0.128064 | | protein_coding | | | XP_026665021.1; XP_026665022.1; XP_008806182.1; XP_017701159.1 | | | probable WRKY transcription factor 2 | | |
| LOC103718774 | -0.33285 | 0.176264 | 0.05898 | | protein_coding | | | XP_008805954.1; XP_008805955.1 | | | protein WRKY1-like | | |
| LOC103717512 | -0.45944 | 0.114232 | 5.77E-05 | | protein_coding | | | XP_008804160.1 | | | WRKY transcription factor WRKY24-like | | |
| LOC103717201 | -0.13699 | 0.143016 | 0.338114 | | protein_coding | | | XP_008803719.1; XP_008803718.1 | | | probable WRKY transcription factor 4 | | |
| LOC103716572 | 0.047244 | 0.104482 | 0.651145 | | protein_coding | | | XP_026664051.1; XP_008802836.1 | | | protein WRKY1 | | |
| LOC103716221 | -0.94888 | 0.312774 | 0.002415 | | protein_coding | | | XP_008802353.1 | | | WRKY transcription factor WRKY71-like | | |
| LOC103715618 | 0.330048 | 0.120711 | 0.006253 | | protein_coding | | | XP_008801524.2 | | | WRKY transcription factor WRKY51-like | | |
| LOC103715016 | 4.554261 | 0.836863 | 5.27E-08 | | protein_coding | | | XP_017700189.1 | | | probable WRKY transcription factor 31 | | |
| LOC103714938 | 0.024607 | 0.120039 | 0.837579 | | protein_coding | | | XP_008800640.1; XP_026663441.1; XP_008800638.1 | | | WRKY transcription factor SUSIBA2-like | | |
| LOC103714242 | 0.13549 | 0.377018 | 0.719316 | | protein_coding | | | XP_008799651.1 | | | WRKY transcription factor WRKY76-like | | |
| LOC103714091 | 0.149516 | 0.144836 | 0.301924 | | protein_coding | | | XP_008799448.1; XP_008799447.1 | | | WRKY transcription factor WRKY51-like | | |
| LOC103713914 | 0.147759 | 0.223714 | 0.508946 | | protein_coding | | | XP_008799188.2 | | | probable WRKY transcription factor 3 | | |
| LOC103713754 | 0.166336 | 0.125576 | 0.18531 | | protein_coding | | | XP_008802713.1 | | | probable WRKY transcription factor 65 | | |
| LOC103713231 | 2.900427 | 0.448733 | 1.02E-10 | | protein_coding | | | XP_008798309.1 | | | WRKY transcription factor 28-like | | |
| LOC103712650 | 1.086684 | 0.72312 | 0.132898 | | protein_coding | | | XP_008797453.1 | | | probable WRKY transcription factor 14 | | |
| LOC103712469 | 1.399714 | 0.224959 | 4.91E-10 | | protein_coding | | | XP_026661036.1 | | | WRKY transcription factor 22-like | | |
| LOC103712162 | -0.02207 | 0.221561 | 0.920635 | | protein_coding | | | XP_008796831.1 | | | probable WRKY transcription factor 17 | | |
| LOC103710925 | -0.33795 | 0.299581 | 0.259283 | | protein_coding | | | XP_017699232.1; XP_008795077.1 | | | WRKY transcription factor 22-like | | |
| LOC103710854 | 0.410953 | 0.25466 | 0.106585 | | protein_coding | | | XP_008794985.2 | | | probable WRKY transcription factor 13 | | |
| LOC103710681 | -3.00847 | 2.43574 | 0.216781 | | protein_coding | | | XP_026661880.1 | | | probable WRKY transcription factor 2 | | |
| LOC103710430 | -0.42393 | 0.176431 | 0.016269 | | protein_coding | | | XP_008794350.1 | | | probable WRKY transcription factor 3 | | |
| LOC103710422 | 5.039311 | 2.173772 | 0.020437 | | protein_coding | | | XP_008794348.1 | | | WRKY transcription factor WRKY76-like | | |
| LOC103710421 | -0.0287 | 0.948484 | 0.975864 | | protein_coding | | | XP_008794347.1 | | | probable WRKY transcription factor 40 | | |
| LOC103709750 | 0.040099 | 0.241585 | 0.868172 | | protein_coding | | | XP_008793465.1 | | | probable WRKY transcription factor 13 | | |
| LOC103709614 | 1.03127 | 0.944716 | 0.275001 | | protein_coding | | | XP_008793281.3 | | | probable WRKY transcription factor 51 | | |
| LOC103709613 | 1.420933 | 0.545935 | 0.009248 | | protein_coding | | | XP_008793280.1 | | | probable WRKY transcription factor 51 | | |
| LOC103709034 | 0.179586 | 0.521883 | 0.730762 | | protein_coding | | | XP_008792410.1 | | | probable WRKY transcription factor 75 | | |
| LOC103709031 | 0.289359 | 0.215574 | 0.179509 | | protein_coding | | | XP_008792407.1; XP_026661134.1 | | | WRKY transcription factor 22-like | | |
| LOC103708975 | -0.00152 | 0.726506 | 0.998325 | | protein_coding | | | XP_026661136.1 | | | probable WRKY transcription factor 65 | | |
| LOC103708974 | -0.33148 | 0.097855 | 0.000705 | | protein_coding | | | XP_008792328.1; XP_008792327.1 | | | probable WRKY transcription factor 65 | | |
| LOC103708913 | -0.39863 | 0.282686 | 0.158491 | | protein_coding | | | XP_008792248.1 | | | probable WRKY transcription factor 70 | | |
| LOC103708865 | -1.08505 | 0.602213 | 0.071581 | | protein_coding | | | XP_008792187.1 | | | WRKY transcription factor WRKY24 | | |
| LOC103708157 | -1.6242 | 1.587424 | 0.306229 | | protein_coding | | | XP_008791211.3 | | | probable WRKY transcription factor 41 | | |
| LOC103707788 | -0.668 | 0.976152 | 0.493775 | | protein_coding | | | XP_008790665.1 | | | WRKY transcription factor WRKY71-like | | |
| LOC103707403 | -0.40528 | 0.134332 | 0.002553 | | protein_coding | | | XP_008790093.1; XP_008790094.1 | | | probable WRKY transcription factor 4 | | |
| LOC103707350 | 0.620082 | 0.227726 | 0.006471 | | protein_coding | | | XP_008790018.1 | | | probable WRKY transcription factor 65 | | |
| LOC103707065 | 0.232015 | 0.314275 | 0.46036 | | protein_coding | | | XP_008789639.1; XP_008789640.1 | | | WRKY transcription factor WRKY51-like | | |
| LOC103706394 | -0.44962 | 0.157114 | 0.004213 | | protein_coding | | | XP_008788702.1 | | | probable WRKY transcription factor 14 | | |
| LOC103706249 | 0.006971 | 0.246541 | 0.977443 | | protein_coding | | | XP_008788525.1 | | | probable WRKY transcription factor 70 | | |
| LOC103704540 | 0.316207 | 0.144109 | 0.02822 | | protein_coding | | | XP_008786102.1; XP_008786101.1; XP_026659415.1; XP_008786100.1 | | | protein WRKY1 | | |
| LOC103704459 | 0.321687 | 0.282203 | 0.254322 | | protein_coding | | | XP_008785961.1; XP_026659378.1; XP_017697605.1 | | | probable WRKY transcription factor 57 | | |
| LOC103702950 | 0.503834 | 0.577319 | 0.38282 | | protein_coding | | | XP_008783826.1 | | | probable WRKY transcription factor 41 | | |
| LOC103702844 | 0.235771 | 0.391289 | 0.546809 | | protein_coding | | | XP_026658703.1; XP_026658701.1; XP_026658702.1; XP_008783654.1 | | | WRKY transcription factor 44-like | | |
| LOC103702224 | 0.800016 | 1.154822 | 0.48846 | | protein_coding | | | XP_008782779.1 | | | probable WRKY transcription factor 70 | | |
| LOC103702223 | -0.2129 | 0.28055 | 0.447937 | | protein_coding | | | XP_017697073.1 | | | probable WRKY transcription factor 70 | | |
| LOC103702139 | -0.36013 | 0.284094 | 0.204929 | | protein_coding | | | XP_008782668.1 | | | probable WRKY transcription factor 70 | | |
| LOC103701508 | 0.103341 | 0.113639 | 0.363147 | | protein_coding | | | XP_008781804.1 | | | WRKY transcription factor WRKY51-like | | |
| LOC103701007 | -0.19517 | 0.156444 | 0.212208 | | protein_coding | | | XP_008781154.1 | | | probable WRKY transcription factor 48 | | |
| LOC103700856 | -0.34812 | 0.185689 | 0.060828 | | protein_coding | | | XP_008780961.2 | | | probable WRKY transcription factor 3 | | |
| LOC103699521 | 0.435851 | 0.242277 | 0.072021 | | protein_coding | | | XP_008779787.1; XP_008779832.1 | | | probable WRKY transcription factor 31 | | |
| LOC103698690 | -0.25123 | 0.112545 | 0.025598 | | protein_coding | | | XP_008778954.1 | | | probable WRKY transcription factor 4 | | |
| LOC103698036 | -0.55163 | 0.157374 | 0.000456 | | protein_coding | | | XP_008778211.2 | | | probable WRKY transcription factor 9 | | |
| LOC103697729 | -0.24951 | 0.208243 | 0.23086 | | protein_coding | | | XP_008777869.1 | | | probable WRKY transcription factor 65 | | |
| LOC103697177 | -0.80404 | 0.401566 | 0.045256 | | protein_coding | | | XP_008777211.3 | | | probable WRKY transcription factor 49 | | |
| LOC103697157 | 2.136727 | 0.439078 | 1.14E-06 | | protein_coding | | | XP_008777186.1 | | | WRKY transcription factor 28-like | | |
| LOC103697011 | 0.416257 | 0.222619 | 0.06151 | | protein_coding | | | XP_008776993.1; XP_008776994.1 | | | probable WRKY transcription factor 57 | | |
| LOC103696886 | -0.16205 | 0.153931 | 0.292447 | | protein_coding | | | XP_008776831.1 | | | probable WRKY transcription factor 48 | | |
| LOC103696470 | -0.49113 | 0.55026 | 0.372104 | | protein_coding | | | XP_008776340.1 | | | WRKY transcription factor 22-like | | |
| LOC103696297 | 0.083262 | 0.50809 | 0.869832 | | protein_coding | | | XP_008776094.1; XP_017695865.1; XP_026656828.1 | | | WRKY transcription factor 44 | | |
| LOC103696206 | 0.492508 | 0.27762 | 0.076057 | | protein_coding | | | XP_008775965.1 | | | WRKY transcription factor 42-like | | |
| LOC103695902 | -0.9066 | 0.345866 | 0.008761 | | protein_coding | | | XP_008775571.1 | | | probable WRKY transcription factor 41 | | |
| LOC103695537 | 0.306053 | 0.467408 | 0.512605 | | protein_coding | | | XP_008775118.1 | | | probable WRKY transcription factor 24 | | |
| Characteristics of differentially expressed WRKY transcription factors in the Salt treated group | | | | | | | | | | | | | |
| Gene_Symbol | log2FoldChange | lfcSE | pvalue | | | Type | | | Protein_ID | | | product | |
| LOC113463770 | 2.44743 | 2.672501 | 0.359781 | | | protein_coding | | | XP_026666347.1 | | | WRKY transcription factor 71-like | |
| LOC113461100 | 4.488014 | 3.090636 | 0.146465 | | | protein_coding | | | XP_026656314.1; XP_026656313.1 | | | probable WRKY transcription factor 75 | |
| LOC108511557 | -0.91061 | 0.53796 | 0.09051 | | | protein_coding | | | XP_026663206.1 | | | WRKY transcription factor WRKY24-like | |
| LOC103724141 | -2.29066 | 0.29255 | 4.88E-15 | | | protein_coding | | | XP_008813530.2 | | | probable WRKY transcription factor 72 | |
| LOC103723396 | -0.47403 | 0.524826 | 0.366416 | | | protein_coding | | | XP_008812523.1 | | | probable WRKY transcription factor 14 | |
| LOC103723227 | 4.388675 | 3.201491 | 0.17043 | | | protein_coding | | | XP_008812299.1 | | | probable WRKY transcription factor 51 | |
| LOC103723223 | 6.797579 | 1.746177 | 9.91E-05 | | | protein_coding | | | XP_008812294.1 | | | probable WRKY transcription factor 50 | |
| LOC103721580 | 3.415531 | 3.955649 | 0.387887 | | | protein_coding | | | XP_008810071.1 | | | probable WRKY transcription factor 50 | |
| LOC103721507 | 0.0457 | 0.240958 | 0.849575 | | | protein_coding | | | XP_008809977.1 | | | WRKY transcription factor WRKY24-like | |
| LOC103721327 | -3.1223 | 0.382965 | 3.55E-16 | | | protein_coding | | | XP_017701724.1; XP_017701728.1 | | | probable WRKY transcription factor 72 | |
| LOC103721211 | 0.428926 | 0.391915 | 0.273764 | | | protein_coding | | | XP_008809548.1 | | | probable WRKY transcription factor 75 | |
| LOC103720998 | -1.69333 | 0.295331 | 9.83E-09 | | | protein_coding | | | XP_008809222.1 | | | probable WRKY transcription factor 14 | |
| LOC103720799 | -1.09464 | 3.589006 | 0.760368 | | | protein_coding | | | XP_008808912.1 | | | probable WRKY transcription factor 50 | |
| LOC103720653 | 1.138917 | 0.950261 | 0.23071 | | | protein_coding | | | XP_008808698.1 | | | probable WRKY transcription factor 13 | |
| LOC103720433 | 1.953552 | 0.784968 | 0.012821 | | | protein_coding | | | XP_026665098.1 | | | probable WRKY transcription factor 72 | |
| LOC103720275 | 1.281308 | 1.270481 | 0.313204 | | | protein_coding | | | XP_008808114.1 | | | probable WRKY transcription factor 75 | |
| LOC103718944 | 0.061466 | 0.114374 | 0.590981 | | | protein_coding | | | XP_026665021.1; XP_026665022.1; XP_008806182.1; XP_017701159.1 | | | probable WRKY transcription factor 2 | |
| LOC103718774 | 0.125841 | 0.238157 | 0.597227 | | | protein_coding | | | XP_008805954.1; XP_008805955.1 | | | protein WRKY1-like | |
| LOC103717512 | 0.023536 | 0.271774 | 0.930988 | | | protein_coding | | | XP_008804160.1 | | | WRKY transcription factor WRKY24-like | |
| LOC103717201 | -0.17359 | 0.159301 | 0.27585 | | | protein_coding | | | XP_008803719.1; XP_008803718.1 | | | probable WRKY transcription factor 4 | |
| LOC103716572 | 0.189254 | 0.158975 | 0.233864 | | | protein_coding | | | XP_026664051.1; XP_008802836.1 | | | protein WRKY1 | |
| LOC103716221 | -0.54757 | 0.586699 | 0.350659 | | | protein_coding | | | XP_008802353.1 | | | WRKY transcription factor WRKY71-like | |
| LOC103715618 | 0.175951 | 0.248838 | 0.47951 | | | protein_coding | | | XP_008801524.2 | | | WRKY transcription factor WRKY51-like | |
| LOC103715016 | 3.720257 | 1.608549 | 0.020733 | | | protein_coding | | | XP_017700189.1 | | | probable WRKY transcription factor 31 | |
| LOC103714938 | -0.10317 | 0.235047 | 0.660725 | | | protein_coding | | | XP_008800640.1; XP_026663441.1; XP_008800638.1 | | | WRKY transcription factor SUSIBA2-like | |
| LOC103714242 | 0.338641 | 0.479947 | 0.48045 | | | protein_coding | | | XP_008799651.1 | | | WRKY transcription factor WRKY76-like | |
| LOC103714091 | -0.71018 | 0.251758 | 0.004789 | | | protein_coding | | | XP_008799448.1; XP_008799447.1 | | | WRKY transcription factor WRKY51-like | |
| LOC103713914 | -0.34972 | 0.258118 | 0.175455 | | | protein_coding | | | XP_008799188.2 | | | probable WRKY transcription factor 3 | |
| LOC103713754 | -0.38304 | 0.166487 | 0.021409 | | | protein_coding | | | XP_008802713.1 | | | probable WRKY transcription factor 65 | |
| LOC103713231 | 3.233827 | 0.862381 | 0.000177 | | | protein_coding | | | XP_008798309.1 | | | WRKY transcription factor 28-like | |
| LOC103712724 | 1.12994 | 2.153585 | 0.599806 | | | protein_coding | | | XP_008797553.1 | | | probable WRKY transcription factor 31 | |
| LOC103712650 | -0.38751 | 0.933689 | 0.67812 | | | protein_coding | | | XP_008797453.1 | | | probable WRKY transcription factor 14 | |
| LOC103712469 | -0.53568 | 0.541389 | 0.322437 | | | protein_coding | | | XP_026661036.1 | | | WRKY transcription factor 22-like | |
| LOC103712162 | -0.0214 | 0.221594 | 0.923064 | | | protein_coding | | | XP_008796831.1 | | | probable WRKY transcription factor 17 | |
| LOC103711983 | 0.244445 | 0.188589 | 0.194913 | | | protein_coding | | | XP_008796564.2; XP_008796561.1; XP_026662352.1; XP_008796562.1; XP_017699506.1; XP_017699505.1 | | | probable WRKY transcription factor 2 | |
| LOC103710925 | -2.76433 | 0.903715 | 0.002222 | | | protein_coding | | | XP_017699232.1; XP_008795077.1 | | | WRKY transcription factor 22-like | |
| LOC103710854 | -0.02712 | 0.289324 | 0.925306 | | | protein_coding | | | XP_008794985.2 | | | probable WRKY transcription factor 13 | |
| LOC103710681 | -5.14142 | 3.677735 | 0.162118 | | | protein_coding | | | XP_026661880.1 | | | probable WRKY transcription factor 2 | |
| LOC103710430 | -0.96427 | 0.226429 | 2.06E-05 | | | protein_coding | | | XP_008794350.1 | | | probable WRKY transcription factor 3 | |
| LOC103710422 | 2.609926 | 1.421454 | 0.066343 | | | protein_coding | | | XP_008794348.1 | | | WRKY transcription factor WRKY76-like | |
| LOC103710421 | 2.195462 | 1.349185 | 0.103684 | | | protein_coding | | | XP_008794347.1 | | | probable WRKY transcription factor 40 | |
| LOC103709750 | -0.27503 | 0.653834 | 0.674013 | | | protein_coding | | | XP_008793465.1 | | | probable WRKY transcription factor 13 | |
| LOC103709614 | 2.43911 | 0.949939 | 0.010239 | | | protein_coding | | | XP_008793281.3 | | | probable WRKY transcription factor 51 | |
| LOC103709613 | 2.168737 | 0.594787 | 0.000266 | | | protein_coding | | | XP_008793280.1 | | | probable WRKY transcription factor 51 | |
| LOC103709034 | -0.66269 | 0.90513 | 0.46408 | | | protein_coding | | | XP_008792410.1 | | | probable WRKY transcription factor 75 | |
| LOC103709031 | -2.70693 | 0.389181 | 3.51E-12 | | | protein_coding | | | XP_008792407.1; XP_026661134.1 | | | WRKY transcription factor 22-like | |
| LOC103708975 | 0.850495 | 0.680943 | 0.211667 | | | protein_coding | | | XP_026661136.1 | | | probable WRKY transcription factor 65 | |
| LOC103708974 | -0.94254 | 0.323036 | 0.003526 | | | protein_coding | | | XP_008792328.1; XP_008792327.1 | | | probable WRKY transcription factor 65 | |
| LOC103708913 | 0.178118 | 0.245332 | 0.467822 | | | protein_coding | | | XP_008792248.1 | | | probable WRKY transcription factor 70 | |
| LOC103708865 | -1.30293 | 0.59444 | 0.028389 | | | protein_coding | | | XP_008792187.1 | | | WRKY transcription factor WRKY24 | |
| LOC103708338 | 1.223025 | 3.922892 | 0.755218 | | | protein_coding | | | XP_008791438.1 | | | probable WRKY transcription factor 53 | |
| LOC103708157 | -1.18191 | 1.830539 | 0.518497 | | | protein_coding | | | XP_008791211.3 | | | probable WRKY transcription factor 41 | |
| LOC103707788 | 2.156743 | 0.74872 | 0.00397 | | | protein_coding | | | XP_008790665.1 | | | WRKY transcription factor WRKY71-like | |
| LOC103707403 | -0.35765 | 0.209927 | 0.088441 | | | protein_coding | | | XP_008790093.1; XP_008790094.1 | | | probable WRKY transcription factor 4 | |
| LOC103707350 | 0.373038 | 0.279513 | 0.182007 | | | protein_coding | | | XP_008790018.1 | | | probable WRKY transcription factor 65 | |
| LOC103707065 | -0.3025 | 0.365326 | 0.407649 | | | protein_coding | | | XP_008789639.1; XP_008789640.1 | | | WRKY transcription factor WRKY51-like | |
| LOC103706394 | -1.21991 | 0.152952 | 1.51E-15 | | | protein_coding | | | XP_008788702.1 | | | probable WRKY transcription factor 14 | |
| LOC103706249 | 0.348231 | 0.202725 | 0.085843 | | | protein_coding | | | XP_008788525.1 | | | probable WRKY transcription factor 70 | |
| LOC103705538 | -1.89016 | 0.288494 | 5.68E-11 | | | protein_coding | | | XP_026658453.1 | | | probable WRKY transcription factor 72 | |
| LOC103705233 | -0.39306 | 0.153645 | 0.010521 | | | protein_coding | | | XP_008787096.1; XP_008787093.1; XP_008787094.1; XP_008787095.1 | | | probable WRKY transcription factor 4 | |
| LOC103704540 | 0.727691 | 0.248115 | 0.003358 | | | protein_coding | | | XP_008786102.1; XP_008786101.1; XP_026659415.1; XP_008786100.1 | | | protein WRKY1 | |
| LOC103704459 | 2.022444 | 0.347487 | 5.88E-09 | | | protein_coding | | | XP_008785961.1; XP_026659378.1; XP_017697605.1 | | | probable WRKY transcription factor 57 | |
| LOC103703339 | 1.199576 | 0.346024 | 0.000527 | | | protein_coding | | | XP_008784385.1 | | | probable WRKY transcription factor 31 | |
| LOC103702950 | 0.763056 | 0.705118 | 0.279178 | | | protein_coding | | | XP_008783826.1 | | | probable WRKY transcription factor 41 | |
| LOC103702844 | 0.707164 | 0.398627 | 0.076063 | | | protein_coding | | | XP_026658703.1; XP_026658701.1; XP_026658702.1; XP_008783654.1 | | | WRKY transcription factor 44-like | |
| LOC103702224 | 1.311847 | 1.032738 | 0.203992 | | | protein_coding | | | XP_008782779.1 | | | probable WRKY transcription factor 70 | |
| LOC103702223 | -2.21394 | 0.310947 | 1.08E-12 | | | protein_coding | | | XP_017697073.1 | | | probable WRKY transcription factor 70 | |
| LOC103702139 | 1.040855 | 0.440328 | 0.018088 | | | protein_coding | | | XP_008782668.1 | | | probable WRKY transcription factor 70 | |
| LOC103701508 | 0.430309 | 0.142522 | 0.002534 | | | protein_coding | | | XP_008781804.1 | | | WRKY transcription factor WRKY51-like | |
| LOC103701007 | 0.447796 | 0.353432 | 0.205157 | | | protein_coding | | | XP_008781154.1 | | | probable WRKY transcription factor 48 | |
| LOC103700856 | -1.02359 | 0.292234 | 0.000461 | | | protein_coding | | | XP_008780961.2 | | | probable WRKY transcription factor 3 | |
| LOC103699722 | -1.7021 | 0.418055 | 4.67E-05 | | | protein_coding | | | XP_008779946.1 | | | probable WRKY transcription factor 14 | |
| LOC103699521 | 0.429084 | 0.412747 | 0.298535 | | | protein_coding | | | XP_008779787.1; XP_008779832.1 | | | probable WRKY transcription factor 31 | |
| LOC103698763 | 5.166891 | 2.844423 | 0.069294 | | | protein_coding | | | XP_008779030.1 | | | probable WRKY transcription factor 75 | |
| LOC103698690 | 0.075528 | 0.140693 | 0.591386 | | | protein_coding | | | XP_008778954.1 | | | probable WRKY transcription factor 4 | |
| LOC103698036 | -2.2553 | 0.4052 | 2.61E-08 | | | protein_coding | | | XP_008778211.2 | | | probable WRKY transcription factor 9 | |
| LOC103697729 | -0.29434 | 0.221176 | 0.18326 | | | protein_coding | | | XP_008777869.1 | | | probable WRKY transcription factor 65 | |
| LOC103697265 | -0.61833 | 3.613365 | 0.864127 | | | protein_coding | | | XP_008777314.1 | | | probable WRKY transcription factor 49 | |
| LOC103697177 | -0.749 | 0.525852 | 0.154344 | | | protein_coding | | | XP_008777211.3 | | | probable WRKY transcription factor 49 | |
| LOC103697157 | 1.173677 | 0.91479 | 0.199491 | | | protein_coding | | | XP_008777186.1 | | | WRKY transcription factor 28-like | |
| LOC103697011 | 1.327309 | 0.233965 | 1.4E-08 | | | protein_coding | | | XP_008776993.1; XP_008776994.1 | | | probable WRKY transcription factor 57 | |
| LOC103696886 | -0.5431 | 0.233106 | 0.019815 | | | protein_coding | | | XP_008776831.1 | | | probable WRKY transcription factor 48 | |
| LOC103696592 | -0.77505 | 0.19453 | 6.77E-05 | | | protein_coding | | | XP_017695965.1 | | | probable WRKY transcription factor 4 | |
| LOC103696470 | -0.19879 | 0.518911 | 0.701654 | | | protein_coding | | | XP_008776340.1 | | | WRKY transcription factor 22-like | |
| LOC103696297 | -0.78653 | 0.672167 | 0.241945 | | | protein_coding | | | XP_008776094.1; XP_017695865.1; XP_026656828.1 | | | WRKY transcription factor 44 | |
| LOC103696206 | 0.136262 | 0.348266 | 0.695605 | | | protein_coding | | | XP_008775965.1 | | | WRKY transcription factor 42-like | |
| LOC103695902 | -0.48847 | 0.356592 | 0.170739 | | | protein_coding | | | XP_008775571.1 | | | probable WRKY transcription factor 41 | |
| LOC103695537 | -1.55087 | 1.215037 | 0.201815 | | | protein_coding | | | XP_008775118.1 | | | probable WRKY transcription factor 24 | |
| Characteristics of differentially expressed WRKY transcription factors in the Fungi+Salt treated group | | | | | | | | | | | | | |
| Gene_Symbol | log2FoldChange | lfcSE | | pvalue | | | Type | | | Protein_ID | | | Product |
| LOC113463770 | 3.498682 | 1.615412 | | 0.030325 | | | protein_coding | | | XP_026666347.1 | | | WRKY transcription factor 71-like |
| LOC108511557 | 0.503751 | 0.364011 | | 0.166393 | | | protein_coding | | | XP_026663206.1 | | | WRKY transcription factor WRKY24-like |
| LOC103724141 | -0.55188 | 0.139666 | | 7.77E-05 | | | protein_coding | | | XP_008813530.2 | | | probable WRKY transcription factor 72 |
| LOC103723396 | 0.237667 | 0.362158 | | 0.511661 | | | protein_coding | | | XP_008812523.1 | | | probable WRKY transcription factor 14 |
| LOC103723223 | 4.890807 | 1.990238 | | 0.013995 | | | protein_coding | | | XP_008812294.1 | | | probable WRKY transcription factor 50 |
| LOC103721507 | 0.520791 | 0.100279 | | 2.06E-07 | | | protein_coding | | | XP_008809977.1 | | | WRKY transcription factor WRKY24-like |
| LOC103721327 | -1.33848 | 0.16674 | | 9.96E-16 | | | protein_coding | | | XP_017701724.1; XP_017701728.1 | | | probable WRKY transcription factor 72 |
| LOC103721211 | 0.831639 | 0.212219 | | 0.000089 | | | protein_coding | | | XP_008809548.1 | | | probable WRKY transcription factor 75 |
| LOC103720998 | 0.103955 | 0.161297 | | 0.519256 | | | protein_coding | | | XP_008809222.1 | | | probable WRKY transcription factor 14 |
| LOC103720799 | -0.89482 | 2.513275 | | 0.721814 | | | protein_coding | | | XP_008808912.1 | | | probable WRKY transcription factor 50 |
| LOC103720653 | 1.876713 | 0.357432 | | 1.52E-07 | | | protein_coding | | | XP_008808698.1 | | | probable WRKY transcription factor 13 |
| LOC103720433 | 2.99856 | 0.535875 | | 2.2E-08 | | | protein_coding | | | XP_026665098.1 | | | probable WRKY transcription factor 72 |
| LOC103720275 | 2.381164 | 0.461475 | | 2.47E-07 | | | protein_coding | | | XP_008808114.1 | | | probable WRKY transcription factor 75 |
| LOC103718944 | 0.034299 | 0.081743 | | 0.674785 | | | protein_coding | | | XP_026665021.1; XP_026665022.1; XP_008806182.1; XP_017701159.1 | | | probable WRKY transcription factor 2 |
| LOC103718774 | 0.786199 | 0.157226 | | 5.72E-07 | | | protein_coding | | | XP_008805954.1; XP_008805955.1 | | | protein WRKY1-like |
| LOC103717512 | 0.286641 | 0.089563 | | 0.001372 | | | protein_coding | | | XP_008804160.1 | | | WRKY transcription factor WRKY24-like |
| LOC103717201 | -0.06685 | 0.134607 | | 0.619462 | | | protein_coding | | | XP_008803719.1; XP_008803718.1 | | | probable WRKY transcription factor 4 |
| LOC103716572 | 0.555523 | 0.089599 | | 5.64E-10 | | | protein_coding | | | XP_026664051.1; XP_008802836.1 | | | protein WRKY1 |
| LOC103716221 | -0.19744 | 0.213032 | | 0.354028 | | | protein_coding | | | XP_008802353.1 | | | WRKY transcription factor WRKY71-like |
| LOC103715618 | 1.085255 | 0.103441 | | 9.45E-26 | | | protein_coding | | | XP_008801524.2 | | | WRKY transcription factor WRKY51-like |
| LOC103715016 | 4.497903 | 0.707782 | | 2.09E-10 | | | protein_coding | | | XP_017700189.1 | | | probable WRKY transcription factor 31 |
| LOC103714938 | -0.48567 | 0.121204 | | 6.15E-05 | | | protein_coding | | | XP_008800640.1; XP_026663441.1; XP_008800638.1 | | | WRKY transcription factor SUSIBA2-like |
| LOC103714242 | 0.54447 | 0.156846 | | 0.000518 | | | protein_coding | | | XP_008799651.1 | | | WRKY transcription factor WRKY76-like |
| LOC103714091 | 0.281219 | 0.1334 | | 0.035023 | | | protein_coding | | | XP_008799448.1; XP_008799447.1 | | | WRKY transcription factor WRKY51-like |
| LOC103713914 | -0.17882 | 0.232455 | | 0.441733 | | | protein_coding | | | XP_008799188.2 | | | probable WRKY transcription factor 3 |
| LOC103713754 | 0.476835 | 0.109189 | | 1.26E-05 | | | protein_coding | | | XP_008802713.1 | | | probable WRKY transcription factor 65 |
| LOC103713231 | 3.13578 | 0.387332 | | 5.69E-16 | | | protein_coding | | | XP_008798309.1 | | | WRKY transcription factor 28-like |
| LOC103712724 | 0.827516 | 1.559462 | | 0.595667 | | | protein_coding | | | XP_008797553.1 | | | probable WRKY transcription factor 31 |
| LOC103712650 | -5.27726 | 1.707596 | | 0.001998 | | | protein_coding | | | XP_008797453.1 | | | probable WRKY transcription factor 14 |
| LOC103712469 | 0.76097 | 0.224628 | | 0.000705 | | | protein_coding | | | XP_026661036.1 | | | WRKY transcription factor 22-like |
| LOC103712162 | -1.62542 | 0.252557 | | 1.23E-10 | | | protein_coding | | | XP_008796831.1 | | | probable WRKY transcription factor 17 |
| LOC103711983 | -0.19875 | 0.139278 | | 0.153571 | | | protein_coding | | | XP_008796564.2; XP_008796561.1; XP_026662352.1; XP_008796562.1; XP_017699506.1; XP_017699505.1 | | | probable WRKY transcription factor 2 |
| LOC103710925 | -1.07274 | 0.326695 | | 0.001025 | | | protein_coding | | | XP_017699232.1; XP_008795077.1 | | | WRKY transcription factor 22-like |
| LOC103710854 | -0.21372 | 0.268043 | | 0.42526 | | | protein_coding | | | XP_008794985.2 | | | probable WRKY transcription factor 13 |
| LOC103710681 | -4.92183 | 2.825581 | | 0.081529 | | | protein_coding | | | XP_026661880.1 | | | probable WRKY transcription factor 2 |
| LOC103710430 | -0.33645 | 0.165309 | | 0.041826 | | | protein_coding | | | XP_008794350.1 | | | probable WRKY transcription factor 3 |
| LOC103710422 | 0.246943 | 1.889941 | | 0.896043 | | | protein_coding | | | XP_008794348.1 | | | WRKY transcription factor WRKY76-like |
| LOC103710421 | -0.30807 | 0.97053 | | 0.750919 | | | protein_coding | | | XP_008794347.1 | | | probable WRKY transcription factor 40 |
| LOC103709750 | -0.08492 | 0.231985 | | 0.714335 | | | protein_coding | | | XP_008793465.1 | | | probable WRKY transcription factor 13 |
| LOC103709614 | 1.550204 | 0.789505 | | 0.049586 | | | protein_coding | | | XP_008793281.3 | | | probable WRKY transcription factor 51 |
| LOC103709613 | 2.070323 | 0.400308 | | 2.32E-07 | | | protein_coding | | | XP_008793280.1 | | | probable WRKY transcription factor 51 |
| LOC103709034 | -2.01976 | 0.668557 | | 0.002519 | | | protein_coding | | | XP_008792410.1 | | | probable WRKY transcription factor 75 |
| LOC103709031 | -0.02183 | 0.204182 | | 0.914843 | | | protein_coding | | | XP_008792407.1; XP_026661134.1 | | | WRKY transcription factor 22-like |
| LOC103708975 | 0.911479 | 0.624614 | | 0.144492 | | | protein_coding | | | XP_026661136.1 | | | probable WRKY transcription factor 65 |
| LOC103708974 | -1.37598 | 0.188198 | | 2.65E-13 | | | protein_coding | | | XP_008792328.1; XP_008792327.1 | | | probable WRKY transcription factor 65 |
| LOC103708913 | -0.97206 | 0.144478 | | 1.72E-11 | | | protein_coding | | | XP_008792248.1 | | | probable WRKY transcription factor 70 |
| LOC103708865 | -0.42034 | 0.467915 | | 0.369017 | | | protein_coding | | | XP_008792187.1 | | | WRKY transcription factor WRKY24 |
| LOC103708157 | -2.13787 | 1.878931 | | 0.255199 | | | protein_coding | | | XP_008791211.3 | | | probable WRKY transcription factor 41 |
| LOC103707788 | -1.42384 | 1.141005 | | 0.212076 | | | protein_coding | | | XP_008790665.1 | | | WRKY transcription factor WRKY71-like |
| LOC103707403 | -0.32198 | 0.154256 | | 0.036857 | | | protein_coding | | | XP_008790093.1; XP_008790094.1 | | | probable WRKY transcription factor 4 |
| LOC103707350 | -0.02263 | 0.232761 | | 0.922545 | | | protein_coding | | | XP_008790018.1 | | | probable WRKY transcription factor 65 |
| LOC103707065 | -0.33801 | 0.318318 | | 0.288294 | | | protein_coding | | | XP_008789639.1; XP_008789640.1 | | | WRKY transcription factor WRKY51-like |
| LOC103706394 | 0.300161 | 0.103169 | | 0.003621 | | | protein_coding | | | XP_008788702.1 | | | probable WRKY transcription factor 14 |
| LOC103706249 | 0.183771 | 0.157138 | | 0.242208 | | | protein_coding | | | XP_008788525.1 | | | probable WRKY transcription factor 70 |
| LOC103705538 | -0.37805 | 0.131266 | | 0.003976 | | | protein_coding | | | XP_026658453.1 | | | probable WRKY transcription factor 72 |
| LOC103705233 | 0.120978 | 0.101277 | | 0.232273 | | | protein_coding | | | XP_008787096.1; XP_008787093.1; XP_008787094.1; XP_008787095.1 | | | probable WRKY transcription factor 4 |
| LOC103704540 | 1.009755 | 0.129256 | | 5.63E-15 | | | protein_coding | | | XP_008786102.1; XP_008786101.1; XP_026659415.1; XP_008786100.1 | | | protein WRKY1 |
| LOC103704459 | 1.012793 | 0.294172 | | 0.000576 | | | protein_coding | | | XP_008785961.1; XP_026659378.1; XP_017697605.1 | | | probable WRKY transcription factor 57 |
| LOC103703339 | 1.609049 | 0.133503 | | 1.88E-33 | | | protein_coding | | | XP_008784385.1 | | | probable WRKY transcription factor 31 |
| LOC103702950 | -0.69724 | 0.650577 | | 0.283845 | | | protein_coding | | | XP_008783826.1 | | | probable WRKY transcription factor 41 |
| LOC103702844 | 0.00892 | 0.370679 | | 0.980802 | | | protein_coding | | | XP_026658703.1; XP_026658701.1; XP_026658702.1; XP_008783654.1 | | | WRKY transcription factor 44-like |
| LOC103702224 | -4.11043 | 2.199636 | | 0.061667 | | | protein_coding | | | XP_008782779.1 | | | probable WRKY transcription factor 70 |
| LOC103702223 | -1.1073 | 0.291154 | | 0.000143 | | | protein_coding | | | XP_017697073.1 | | | probable WRKY transcription factor 70 |
| LOC103702139 | 1.427675 | 0.201787 | | 1.49E-12 | | | protein_coding | | | XP_008782668.1 | | | probable WRKY transcription factor 70 |
| LOC103702138 | 4.997539 | 2.003657 | | 0.012624 | | | protein_coding | | | XP_008782667.1 | | | probable WRKY transcription factor 46 |
| LOC103701508 | 0.192052 | 0.100507 | | 0.056027 | | | protein_coding | | | XP_008781804.1 | | | WRKY transcription factor WRKY51-like |
| LOC103701007 | 0.857065 | 0.10861 | | 2.99E-15 | | | protein_coding | | | XP_008781154.1 | | | probable WRKY transcription factor 48 |
| LOC103700856 | -0.7144 | 0.18027 | | 0.000074 | | | protein_coding | | | XP_008780961.2 | | | probable WRKY transcription factor 3 |
| LOC103699722 | -0.19002 | 0.151372 | | 0.209357 | | | protein_coding | | | XP_008779946.1 | | | probable WRKY transcription factor 14 |
| LOC103699521 | 0.604434 | 0.192596 | | 0.001699 | | | protein_coding | | | XP_008779787.1; XP_008779832.1 | | | probable WRKY transcription factor 31 |
| LOC103698763 | 6.660126 | 1.470419 | | 5.91E-06 | | | protein_coding | | | XP_008779030.1 | | | probable WRKY transcription factor 75 |
| LOC103698690 | 0.373287 | 0.093764 | | 6.86E-05 | | | protein_coding | | | XP_008778954.1 | | | probable WRKY transcription factor 4 |
| LOC103698036 | -0.54476 | 0.130495 | | 2.99E-05 | | | protein_coding | | | XP_008778211.2 | | | probable WRKY transcription factor 9 |
| LOC103697729 | 0.267528 | 0.155266 | | 0.084884 | | | protein_coding | | | XP_008777869.1 | | | probable WRKY transcription factor 65 |
| LOC103697265 | -3.12921 | 3.950189 | | 0.428263 | | | protein_coding | | | XP_008777314.1 | | | probable WRKY transcription factor 49 |
| LOC103697177 | 1.182829 | 0.310652 | | 0.00014 | | | protein_coding | | | XP_008777211.3 | | | probable WRKY transcription factor 49 |
| LOC103697157 | 1.747562 | 0.366253 | | 1.83E-06 | | | protein_coding | | | XP_008777186.1 | | | WRKY transcription factor 28-like |
| LOC103697011 | 1.214633 | 0.176243 | | 5.51E-12 | | | protein_coding | | | XP_008776993.1; XP_008776994.1 | | | probable WRKY transcription factor 57 |
| LOC103696886 | -0.06712 | 0.142532 | | 0.637691 | | | protein_coding | | | XP_008776831.1 | | | probable WRKY transcription factor 48 |
| LOC103696592 | -0.23561 | 0.113355 | | 0.037663 | | | protein_coding | | | XP_017695965.1 | | | probable WRKY transcription factor 4 |
| LOC103696470 | 0.267026 | 0.416803 | | 0.521748 | | | protein_coding | | | XP_008776340.1 | | | WRKY transcription factor 22-like |
| LOC103696297 | -0.89694 | 0.587296 | | 0.126703 | | | protein_coding | | | XP_008776094.1; XP_017695865.1; XP_026656828.1 | | | WRKY transcription factor 44 |
| LOC103696206 | -0.02489 | 0.336865 | | 0.941097 | | | protein_coding | | | XP_008775965.1 | | | WRKY transcription factor 42-like |
| LOC103695902 | -0.55578 | 0.31072 | | 0.073663 | | | protein_coding | | | XP_008775571.1 | | | probable WRKY transcription factor 41 |
| LOC103695537 | -2.20515 | 0.606824 | | 0.000279 | | | protein_coding | | | XP_008775118.1 | | | probable WRKY transcription factor 24 |

Table 8: differentially expressed MYB transcription factors in the date palm treatment groups

| Characteristics of differentially expressed MYB transcription factors in the Fungi treated group | | | | | | | | | | | | | | | | | | | |
| --- | --- | --- | --- | --- | --- | --- | --- | --- | --- | --- | --- | --- | --- | --- | --- | --- | --- | --- | --- |
| Gene_Symbol | log2FoldChange | | | lfcSE | | | stat | pvalue | | | | Type | | | Protein_ID | | | Product | |
| LOC103720469 | -1.33986 | | | 0.156074 | | | -8.58478 | 9.1E-18 | | | | protein_coding | | | XP_008808405.1 | | | myb-related protein Hv1-like | |
| LOC103720775 | -1.18219 | | | 0.184849 | | | -6.39542 | 1.6E-10 | | | | protein_coding | | | XP_008808878.1 | | | myb-related protein 308 | |
| LOC103716390 | 1.360684 | | | 0.24305 | | | 5.598376 | 2.16E-08 | | | | protein_coding | | | XP_017700511.1; XP_017700513.1; XP_017700512.1; XP_008802582.1; XP_008802581.1; XP_017700514.1; XP_017700515.1 | | | myb-related protein 2-like | |
| LOC103723368 | -7.2676 | | | 1.434403 | | | -5.06664 | 4.05E-07 | | | | protein_coding | | | XP_008812482.1 | | | myb-related protein 305-like | |
| LOC103719961 | 1.714364 | | | 0.425628 | | | 4.027846 | 5.63E-05 | | | | protein_coding | | | XP_008807672.1 | | | myb-related protein 308-like | |
| LOC103713917 | 4.073227 | | | 1.135682 | | | 3.58659 | 0.000335 | | | | protein_coding | | | XP_008799190.1 | | | myb-related protein 308-like | |
| LOC103708148 | 2.411069 | | | 0.845359 | | | 2.852123 | 0.004343 | | | | protein_coding | | | XP_008791156.1 | | | myb-related protein Hv33-like | |
| LOC103714603 | 3.205467 | | | 1.173006 | | | 2.732694 | 0.006282 | | | | protein_coding | | | XP_008800133.1 | | | myb-related protein Hv1-like | |
| LOC103701316 | 1.124573 | | | 0.418682 | | | 2.685982 | 0.007232 | | | | protein_coding | | | XP_008781556.1 | | | myb-related protein 308-like | |
| LOC103696506 | -0.66089 | | | 0.18328 | | | -3.60591 | 0.000311 | | | | protein_coding | | | XP_008776389.1 | | | myb-related protein MYBAS2-like | |
| LOC103705940 | -0.53662 | | | 0.160857 | | | -3.33603 | 0.00085 | | | | protein_coding | | | XP_008788078.1; XP_026660015.1 | | | myb-related protein MYBAS2 | |
| LOC103703205 | 2.277392 | | | 1.269937 | | | 1.793312 | 0.072923 | | | | protein_coding | | | XP_008784211.1 | | | myb-related protein 306 | |
| LOC103709655 | 0.404375 | | | 0.228759 | | | 1.767688 | 0.077113 | | | | protein_coding | | | XP_008793332.1; XP_017698938.1; XP_008793331.1; XP_026661444.1 | | | myb-related protein 2 | |
| LOC113463338 | 2.200245 | | | 1.28073 | | | 1.717962 | 0.085804 | | | | protein_coding | | | XP_026664012.1 | | | myb-related protein 306-like | |
| LOC103702922 | -1.24124 | | | 0.781721 | | | -1.58783 | 0.112326 | | | | protein_coding | | | XP_008783782.1 | | | myb-related protein 340-like | |
| LOC103704727 | 0.692025 | | | 0.440041 | | | 1.572636 | 0.115803 | | | | protein_coding | | | XP_008786362.1 | | | myb-related protein P-like | |
| LOC103709092 | -1.10775 | | | 0.707203 | | | -1.56639 | 0.117258 | | | | protein_coding | | | XP_026661214.1; XP_017698819.2 | | | myb-related protein 2-like | |
| LOC103709300 | 0.42527 | | | 0.33619 | | | 1.264966 | 0.205883 | | | | protein_coding | | | XP_008792804.1 | | | myb-related protein Hv33 | |
| LOC103696754 | 1.278136 | | | 1.134956 | | | 1.126155 | 0.2601 | | | | protein_coding | | | XP_008776690.1 | | | myb-related protein Zm1-like | |
| LOC103714577 | 1.690364 | | | 1.511878 | | | 1.118056 | 0.263543 | | | | protein_coding | | | XP_008800091.1 | | | myb-related protein Zm1-like | |
| LOC103701054 | -0.42831 | | | 0.425041 | | | -1.00768 | 0.313608 | | | | protein_coding | | | XP_008781220.2; XP_008781221.2 | | | myb-related protein 2-like | |
| LOC103703771 | 0.582729 | | | 0.619737 | | | 0.940284 | 0.347072 | | | | protein_coding | | | XP_026659090.1 | | | myb-related protein Hv33-like | |
| LOC103720607 | -0.22095 | | | 0.252425 | | | -0.87533 | 0.381396 | | | | protein_coding | | | XP_008808618.1 | | | myb-related protein Hv1 | |
| LOC103719749 | -0.149 | | | 0.251632 | | | -0.59215 | 0.553751 | | | | protein_coding | | | XP_026665294.1; XP_017701356.1; XP_008807359.1; XP_008807360.1; XP_026665293.1 | | | myb-related protein 2-like | |
| LOC103702988 | -0.17758 | | | 0.354715 | | | -0.50061 | 0.616643 | | | | protein_coding | | | XP_008783889.1; XP_017697086.1; XP_026658864.1 | | | myb-related protein 2-like | |
| LOC103711302 | -0.10193 | | | 0.326559 | | | -0.31213 | 0.754943 | | | | protein_coding | | | XP_008795619.1 | | | myb-related protein MYBAS1-like | |
| LOC103706180 | 0.124531 | | | 0.681519 | | | 0.182726 | 0.855013 | | | | protein_coding | | | XP_008788440.1 | | | myb-related protein Hv1-like | |
| LOC103711433 | -0.05114 | | | 0.341457 | | | -0.14977 | 0.880946 | | | | protein_coding | | | XP_026662314.1; XP_008795797.1 | | | myb-related protein MYBAS1-like | |
| LOC103700432 | 0.095642 | | | 0.717803 | | | 0.133243 | 0.894001 | | | | protein_coding | | | XP_008780601.1 | | | myb-related protein 306-like | |
| Characteristics of differentially expressed MYB transcription factors in the Salt treated group | | | | | | | | | | | | | | | | | | | |
| Gene_Symbol | log2FoldChange | | | lfcSE | | stat | | | | pvalue | | Type | | Protein_ID | | | Product | | |
| LOC103702988 | -0.1047 | | | 0.395752 | | -0.26457 | | | | 0.791343 | | protein_coding | | XP_008783889.1; XP_017697086.1; XP_026658864.1 | | | myb-related protein 2-like | | |
| LOC103703205 | 1.686015 | | | 2.230799 | | 0.75579 | | | | 0.449775 | | protein_coding | | XP_008784211.1 | | | myb-related protein 306 | | |
| LOC103711302 | 1.959483 | | | 0.20448 | | 9.582779 | | | | 9.45E-22 | | protein_coding | | XP_008795619.1 | | | myb-related protein MYBAS1-like | | |
| LOC103711433 | 0.555355 | | | 0.410919 | | 1.351494 | | | | 0.176537 | | protein_coding | | XP_026662314.1; XP_008795797.1 | | | myb-related protein MYBAS1-like | | |
| LOC103696506 | -1.63508 | | | 0.260495 | | -6.27681 | | | | 3.46E-10 | | protein_coding | | XP_008776389.1 | | | myb-related protein MYBAS2-like | | |
| LOC103700432 | 1.574042 | | | 0.493315 | | 3.190742 | | | | 0.001419 | | protein_coding | | XP_008780601.1 | | | myb-related protein 306-like | | |
| LOC103701054 | 0.968935 | | | 0.431338 | | 2.246349 | | | | 0.024682 | | protein_coding | | XP_008781220.2; XP_008781221.2 | | | myb-related protein 2-like | | |
| LOC103701316 | 0.376953 | | | 0.739007 | | 0.51008 | | | | 0.609995 | | protein_coding | | XP_008781556.1 | | | myb-related protein 308-like | | |
| LOC103702922 | -0.07584 | | | 0.741849 | | -0.10223 | | | | 0.918574 | | protein_coding | | XP_008783782.1 | | | myb-related protein 340-like | | |
| LOC103703771 | 0.515906 | | | 0.728487 | | 0.708189 | | | | 0.478828 | | protein_coding | | XP_026659090.1 | | | myb-related protein Hv33-like | | |
| LOC103704727 | -0.29695 | | | 0.67492 | | -0.43998 | | | | 0.659953 | | protein_coding | | XP_008786362.1 | | | myb-related protein P-like | | |
| LOC103705940 | -0.84323 | | | 0.192387 | | -4.38296 | | | | 1.17E-05 | | protein_coding | | XP_008788078.1; XP_026660015.1 | | | myb-related protein MYBAS2 | | |
| LOC103706180 | 0.333855 | | | 0.69994 | | 0.476977 | | | | 0.633379 | | protein_coding | | XP_008788440.1 | | | myb-related protein Hv1-like | | |
| LOC103708148 | 2.617369 | | | 0.909064 | | 2.879193 | | | | 0.003987 | | protein_coding | | XP_008791156.1 | | | myb-related protein Hv33-like | | |
| LOC103709092 | -0.27365 | | | 0.773791 | | -0.35365 | | | | 0.723604 | | protein_coding | | XP_026661214.1; XP_017698819.2 | | | myb-related protein 2-like | | |
| LOC103709300 | -0.04339 | | | 0.443112 | | -0.09792 | | | | 0.921994 | | protein_coding | | XP_008792804.1 | | | myb-related protein Hv33 | | |
| LOC103709655 | 0.740676 | | | 0.38622 | | 1.917756 | | | | 0.055142 | | protein_coding | | XP_008793332.1; XP_017698938.1; XP_008793331.1; XP_026661444.1 | | | myb-related protein 2 | | |
| LOC103712448 | 0.609899 | | | 3.882532 | | 0.157088 | | | | 0.875176 | | protein_coding | | XP_008797192.1 | | | myb-related protein P | | |
| LOC103712917 | 5.137396 | | | 2.80339 | | 1.832566 | | | | 0.066867 | | protein_coding | | XP_008797850.1 | | | myb-related protein 308-like | | |
| LOC103713917 | 3.119237 | | | 1.626206 | | 1.918106 | | | | 0.055098 | | protein_coding | | XP_008799190.1 | | | myb-related protein 308-like | | |
| LOC103714577 | 0.226515 | | | 2.062222 | | 0.10984 | | | | 0.912536 | | protein_coding | | XP_008800091.1 | | | myb-related protein Zm1-like | | |
| LOC103714603 | 3.144446 | | | 1.419284 | | 2.215516 | | | | 0.026725 | | protein_coding | | XP_008800133.1 | | | myb-related protein Hv1-like | | |
| LOC103715730 | 2.426329 | | | 1.923226 | | 1.261593 | | | | 0.207095 | | protein_coding | | XP_008801687.1 | | | myb-related protein 306-like | | |
| LOC103716390 | 0.209832 | | | 0.341577 | | 0.614306 | | | | 0.539013 | | protein_coding | | XP_017700511.1; XP_017700513.1; XP_017700512.1; XP_008802582.1; XP_008802581.1; XP_017700514.1; XP_017700515.1 | | | myb-related protein 2-like | | |
| LOC113463338 | -3.14569 | | | 3.505858 | | -0.89727 | | | | 0.369576 | | protein_coding | | XP_026664012.1 | | | myb-related protein 306-like | | |
| LOC103719749 | 0.545135 | | | 0.280203 | | 1.945505 | | | | 0.051714 | | protein_coding | | XP_026665294.1; XP_017701356.1; XP_008807359.1; XP_008807360.1; XP_026665293.1 | | | myb-related protein 2-like | | |
| LOC103719961 | 0.200926 | | | 0.955415 | | 0.210302 | | | | 0.833432 | | protein_coding | | XP_008807672.1 | | | myb-related protein 308-like | | |
| LOC103720469 | -1.01391 | | | 0.335794 | | -3.01943 | | | | 0.002532 | | protein_coding | | XP_008808405.1 | | | myb-related protein Hv1-like | | |
| LOC103720607 | -0.8518 | | | 0.567492 | | -1.501 | | | | 0.133356 | | protein_coding | | XP_008808618.1 | | | myb-related protein Hv1 | | |
| LOC103720775 | -1.6182 | | | 0.409888 | | -3.9479 | | | | 7.88E-05 | | protein_coding | | XP_008808878.1 | | | myb-related protein 308 | | |
| LOC103723368 | -5.15608 | | | 1.320657 | | -3.90418 | | | | 9.45E-05 | | protein_coding | | XP_008812482.1 | | | myb-related protein 305-like | | |
| LOC103696754 | -0.34421 | | | 1.949898 | | -0.17653 | | | | 0.85988 | | protein_coding | | XP_008776690.1 | | | myb-related protein Zm1-like | | |
| LOC108510996 | -0.87257 | | | 3.926627 | | -0.22222 | | | | 0.824143 | | protein_coding | | XP_017696697.1 | | | myb-related protein P-like | | |
| Characteristics of differentially expressed MYB transcription factors in the Salt treated group | | | | | | | | | | | | | | | | | | |  |
| Gene_Symbol | | log2FoldChange | lfcSE | | pvalue | | | | padj | | Type | | Protein_ID | | | Product | | |  |
| LOC103702988 | | -0.68877 | 0.40526 | | 0.089211 | | | | 0.194463 | | protein_coding | | XP_008783889.1; XP_017697086.1; XP_026658864.1 | | | myb-related protein 2-like | | |  |
| LOC103703205 | | 1.690798 | 1.444629 | | 0.241839 | | | | 0.407109 | | protein_coding | | XP_008784211.1 | | | myb-related protein 306 | | |  |
| LOC103711302 | | 2.004679 | 0.165512 | | 9.13E-34 | | | | 9.68E-32 | | protein_coding | | XP_008795619.1 | | | myb-related protein MYBAS1-like | | |  |
| LOC103711433 | | -0.23994 | 0.733942 | | 0.743726 | | | | 0.850823 | | protein_coding | | XP_026662314.1; XP_008795797.1 | | | myb-related protein MYBAS1-like | | |  |
| LOC103696506 | | 0.111732 | 0.163877 | | 0.495363 | | | | 0.661319 | | protein_coding | | XP_008776389.1 | | | myb-related protein MYBAS2-like | | |  |
| LOC103700432 | | 0.316721 | 0.594612 | | 0.594275 | | | | 0.744951 | | protein_coding | | XP_008780601.1 | | | myb-related protein 306-like | | |  |
| LOC103701054 | | -0.27402 | 0.429272 | | 0.52325 | | | | 0.684814 | | protein_coding | | XP_008781220.2; XP_008781221.2 | | | myb-related protein 2-like | | |  |
| LOC103701316 | | -3.59058 | 1.019602 | | 0.000429 | | | | 0.002217 | | protein_coding | | XP_008781556.1 | | | myb-related protein 308-like | | |  |
| LOC103702922 | | -0.11906 | 0.711543 | | 0.867108 | | | | 0.926952 | | protein_coding | | XP_008783782.1 | | | myb-related protein 340-like | | |  |
| LOC103703771 | | -7.04806 | 1.469307 | | 1.61E-06 | | | | 1.45E-05 | | protein_coding | | XP_026659090.1 | | | myb-related protein Hv33-like | | |  |
| LOC103704727 | | -0.37704 | 0.570562 | | 0.50873 | | | | 0.672839 | | protein_coding | | XP_008786362.1 | | | myb-related protein P-like | | |  |
| LOC103705940 | | -0.38795 | 0.147188 | | 0.008395 | | | | 0.028744 | | protein_coding | | XP_008788078.1; XP_026660015.1 | | | myb-related protein MYBAS2 | | |  |
| LOC103706180 | | 0.426281 | 0.69778 | | 0.541259 | | | | 0.700485 | | protein_coding | | XP_008788440.1 | | | myb-related protein Hv1-like | | |  |
| LOC103708148 | | -3.99017 | 2.280893 | | 0.080223 | | | | 0.178711 | | protein_coding | | XP_008791156.1 | | | myb-related protein Hv33-like | | |  |
| LOC103709092 | | -2.57066 | 0.806288 | | 0.001431 | | | | 0.006367 | | protein_coding | | XP_026661214.1; XP_017698819.2 | | | myb-related protein 2-like | | |  |
| LOC103709300 | | -0.914 | 0.340326 | | 0.007239 | | | | 0.025438 | | protein_coding | | XP_008792804.1 | | | myb-related protein Hv33 | | |  |
| LOC103709655 | | 0.76557 | 0.217815 | | 0.00044 | | | | 0.002265 | | protein_coding | | XP_008793332.1; XP_017698938.1; XP_008793331.1; XP_026661444.1 | | | myb-related protein 2 | | |  |
| LOC103712917 | | 4.204535 | 2.73936 | | 0.124818 | | | | 0.251015 | | protein_coding | | XP_008797850.1 | | | myb-related protein 308-like | | |  |
| LOC103713917 | | 2.026706 | 1.702173 | | 0.233788 | | | | 0.397467 | | protein_coding | | XP_008799190.1 | | | myb-related protein 308-like | | |  |
| LOC103714577 | | -0.38847 | 2.192489 | | 0.859366 | | | | 0.92368 | | protein_coding | | XP_008800091.1 | | | myb-related protein Zm1-like | | |  |
| LOC103714603 | | 2.235249 | 1.421875 | | 0.115941 | | | | 0.237745 | | protein_coding | | XP_008800133.1 | | | myb-related protein Hv1-like | | |  |
| LOC103715005 | | 4.186614 | 3.38922 | | 0.216729 | | | | 0.376495 | | protein_coding | | XP_008800726.1 | | | myb-related protein 305-like | | |  |
| LOC103716390 | | -3.39801 | 0.540853 | | 3.33E-10 | | | | 5.32E-09 | | protein_coding | | XP_017700511.1; XP_017700513.1; XP_017700512.1; XP_008802582.1; XP_008802581.1; XP_017700514.1; XP_017700515.1 | | | myb-related protein 2-like | | |  |
| LOC113463338 | | -0.0334 | 2.541389 | | 0.989515 | | | | 0.99428 | | protein_coding | | XP_026664012.1 | | | myb-related protein 306-like | | |  |
| LOC103719749 | | 0.532135 | 0.229321 | | 0.020315 | | | | 0.059627 | | protein_coding | | XP_026665294.1; XP_017701356.1; XP_008807359.1; XP_008807360.1; XP_026665293.1 | | | myb-related protein 2-like | | |  |
| LOC103719961 | | -0.37959 | 0.703565 | | 0.589525 | | | | 0.740585 | | protein_coding | | XP_008807672.1 | | | myb-related protein 308-like | | |  |
| LOC103720469 | | -0.48644 | 0.13472 | | 0.000305 | | | | 0.001636 | | protein_coding | | XP_008808405.1 | | | myb-related protein Hv1-like | | |  |
| LOC103720607 | | -1.09041 | 0.269843 | | 5.33E-05 | | | | 0.000347 | | protein_coding | | XP_008808618.1 | | | myb-related protein Hv1 | | |  |
| LOC103720775 | | -1.04718 | 0.182438 | | 9.47E-09 | | | | 1.24E-07 | | protein_coding | | XP_008808878.1 | | | myb-related protein 308 | | |  |
| LOC103723368 | | -7.31325 | 1.440123 | | 3.81E-07 | | | | 3.84E-06 | | protein_coding | | XP_008812482.1 | | | myb-related protein 305-like | | |  |
| LOC103696754 | | -4.20813 | 2.564102 | | 0.100762 | | | | 0.213529 | | protein_coding | | XP_008776690.1 | | | myb-related protein Zm1-like | | |  |
| LOC108510996 | | -1.58147 | 3.559162 | | 0.656798 | | | | 0.791767 | | protein_coding | | XP_017696697.1 | | | myb-related protein P-like | | |  |

Table S9: Differentially expressed genes involved in jasmonate synthesis of the different date palm groups

| Characteristics of differentially expressed genes involved in jasmonate synthesis in the Fungi treated group | | | | | | | | | | | | | | | | | | | | | |
| --- | --- | --- | --- | --- | --- | --- | --- | --- | --- | --- | --- | --- | --- | --- | --- | --- | --- | --- | --- | --- | --- |
| Gene_Symbol | | log2FoldChange | | | | lfcSE | | | stat | | pvalue | | | Type | | | Protein_ID | | | Product | |
| LOC103708705 | | 1.433273 | | | | 0.177859 | | | 8.058466 | | 7.73E-16 | | | protein_coding | | | XP_017696990.1; XP_026662013.1; XP_026662029.1; XP_008792017.1 | | | jasmonic acid-amido synthetase JAR1-like | |
| LOC103716872 | | 0.821671 | | | | 0.335616 | | | 2.448249 | | 0.014355 | | | protein_coding | | | XP_026664418.1; XP_008803285.1; XP_017700619.1; XP_017700616.1; XP_017700611.1 | | | jasmonic acid-amido synthetase JAR1-like | |
| Characteristics of differentially expressed genes involved in jasmonate synthesis in the Salt treated group | | | | | | | | | | | | | | | | | | | | | |
| Gene_Symbol | | | log2FoldChange | | lfcSE | | | stat | | pvalue | | | Type | | | Protein_ID | | | Product | | |
| LOC103699381 | | | 1.7768 | | 2.884335 | | | 0.616017 | | 0.537883 | | | Protein_coding | | | XP_008779626.1 | | | 60 kDa jasmonate-induced protein-like | | |
| LOC103708705 | | | 0.274946 | | 0.282862 | | | 0.972015 | | 0.331043 | | | Protein_coding | | | XP_017696990.1; XP_026662013.1; XP_026662029.1; XP_008792017.1 | | | jasmonic acid-amido synthetase JAR1-like | | |
| LOC103716872 | | | 0.06572 | | 0.413718 | | | 0.158853 | | 0.873785 | | | Protein_coding | | | XP_026664418.1; XP_008803285.1; XP_017700619.1; XP_017700616.1; XP_017700611.1 | | | jasmonic acid-amido synthetase JAR1-like | | |
| LOC103708344 | | | -0.40412 | | 0.140741 | | | -2.87139 | | 0.004087 | | | Protein_coding | | | XP_008791451.1; XP_008791450.1; XP_008791449.1; XP_026660926.1; XP_026660927.1 | | | jasmonic acid-amido synthetase JAR1-like | | |
| Characteristics of differentially expressed genes involved in jasmonate synthesis in the Fungi+Salt treated group | | | | | | | | | | | | | | | | | | | | | |
| Type | log2FoldChange | | | lfcSE | | | pvalue | | | | | Type | | | padj | | | Protein_ID | | | Product |
| LOC103708705 | 1.579322 | | | 0.143831 | | | 4.75E-28 | | | | | Protein_coding | | | 3.47E-26 | | | XP_017696990.1; XP_026662013.1; XP_026662029.1; XP_008792017.1 | | | jasmonic acid-amido synthetase JAR1-like |
| LOC103708344 | -0.20354 | | | 0.067831 | | | 0.002694 | | | | | Protein_coding | | | 0.010959 | | | XP_008791451.1; XP_008791450.1; XP_008791449.1; XP_026660926.1; XP_026660927.1 | | | jasmonic acid-amido synthetase JAR1-like |
| LOC103716872 | -1.71746 | | | 0.44907 | | | 0.000131 | | | | | Protein_coding | | | 0.000773 | | | XP_026664418.1; XP_008803285.1; XP_017700619.1; XP_017700616.1; XP_017700611.1 | | | jasmonic acid-amido synthetase JAR1-like |

Table S10: Differentially expressed genes of the lipoxygenase biosynthesis in the date palm treatment groups

| Characteristics of differentially expressed genes involved in lipoxygenase biosynthesis in the Fungi treated group | | | | | | | | | | | | | | | |
| --- | --- | --- | --- | --- | --- | --- | --- | --- | --- | --- | --- | --- | --- | --- | --- |
| Gene_Symbol | log2FoldChange | | lfcSE | stat | pvalue | | | Type | | Protein_ID | | | | Product | |
| LOC103697518 | 1.107506 | | 0.389871 | 2.840696 | 0.004502 | | | protein_coding | | XP_008777614.1 | | | | linoleate 9S-lipoxygenase 6-like | |
| LOC103703268 | 0.954797 | | 0.481608 | 1.982519 | 0.047421 | | | protein_coding | | XP_008784273.1 | | | | putative lipoxygenase 5 | |
| LOC103705383 | 0.445885 | | 0.770309 | 0.578839 | 0.562698 | | | protein_coding | | XP_008787302.1 | | | | linoleate 13S-lipoxygenase 2-1, chloroplastic-like | |
| LOC103702133 | 0.403159 | | 0.145285 | 2.77496 | 0.005521 | | | protein_coding | | XP_008782663.1 | | | | probable lipoxygenase 6 | |
| LOC103708655 | 0.110296 | | 0.446561 | 0.24699 | 0.804916 | | | protein_coding | | XP_008791914.1 | | | | putative lipoxygenase 5 | |
| LOC103719871 | 0.105415 | | 0.120585 | 0.874194 | 0.382013 | | | protein_coding | | XP_008807551.1; XP_008807550.1 | | | | lipoxygenase 6, chloroplastic | |
| LOC103708564 | 0.048015 | | 0.152596 | 0.314653 | 0.753025 | | | protein_coding | | XP_008791769.1 | | | | probable linoleate 9S-lipoxygenase 4 | |
| LOC103715746 | -0.02766 | | 0.10234 | -0.27032 | 0.786912 | | | protein_coding | | XP_008801705.1 | | | | probable linoleate 9S-lipoxygenase 4 | |
| Characteristics of differentially expressed genes involved in lipoxygenase biosynthesis in the Salt treated group | | | | | | | | | | | | | | | |
| gene | | log2FoldChange | lfcSE | stat | | pvalue | padj | | Protein_ID | | | Product | | | |
| LOC103719871 | | -0.42565 | 0.332669 | -1.27951 | | 0.200719 | 0.446951 | | XP_008807551.1; XP_008807550.1 | | | lipoxygenase 6, chloroplastic | | | |
| LOC103715746 | | -0.02933 | 0.188373 | -0.15568 | | 0.876282 | 0.949722 | | XP_008801705.1 | | | probable linoleate 9S-lipoxygenase 4 | | | |
| LOC103708655 | | 0.552596 | 0.486427 | 1.136032 | | 0.255943 | 0.515455 | | XP_008791914.1 | | | putative lipoxygenase 5 | | | |
| LOC103708564 | | 0.082849 | 0.272812 | 0.303685 | | 0.761368 | 0.894616 | | XP_008791769.1 | | | probable linoleate 9S-lipoxygenase 4 | | | |
| LOC103705383 | | -1.24867 | 1.087367 | -1.14834 | | 0.250829 | 0.508429 | | XP_008787302.1 | | | linoleate 13S-lipoxygenase 2-1, chloroplastic-like | | | |
| LOC103703268 | | 1.273622 | 0.689752 | 1.846492 | | 0.064821 | 0.224016 | | XP_008784273.1 | | | putative lipoxygenase 5 | | | |
| LOC103702133 | | 0.569649 | 0.163345 | 3.487393 | | 0.000488 | 0.005987 | | XP_008782663.1 | | | probable lipoxygenase 6 | | | |
| LOC103697518 | | -1.60613 | 0.527718 | -3.04353 | | 0.002338 | 0.020335 | | XP_008777614.1 | | | linoleate 9S-lipoxygenase 6-like | | | |
| Characteristics of differentially expressed genes involved in lipoxygenase biosynthesis in the Fungi+Salt treated group | | | | | | | | | | | | | | |  |
| Gene_Symbol | | log2FoldChange | lfcSE | pvalue | | padj | Type | | | | Protein_ID | | Product | |  |
| LOC103719871 | | -1.01196 | 0.120147 | 3.68E-17 | | 1.21E-15 | Protein_coding | | | | XP_008807551.1; XP_008807550.1 | | lipoxygenase 6, chloroplastic | |  |
| LOC103715746 | | -0.23779 | 0.083381 | 0.004347 | | 0.016537 | Protein_coding | | | | XP_008801705.1 | | probable linoleate 9S-lipoxygenase 4 | |  |
| LOC103708655 | | 0.498937 | 0.40582 | 0.218902 | | 0.379371 | Protein_coding | | | | XP_008791914.1 | | putative lipoxygenase 5 | |  |
| LOC103708564 | | -0.26527 | 0.139567 | 0.057345 | | 0.13733 | Protein_coding | | | | XP_008791769.1 | | probable linoleate 9S-lipoxygenase 4 | |  |
| LOC103705383 | | -5.817 | 1.608559 | 0.000299 | | 0.001606 | Protein_coding | | | | XP_008787302.1 | | linoleate 13S-lipoxygenase 2-1, chloroplastic-like | |  |
| LOC103703268 | | -0.43597 | 0.518299 | 0.400264 | | 0.576669 | Protein_coding | | | | XP_008784273.1 | | putative lipoxygenase 5 | |  |
| LOC103702133 | | 0.683221 | 0.12238 | 2.37E-08 | | 2.89E-07 | Protein_coding | | | | XP_008782663.1 | | probable lipoxygenase 6 | |  |
| LOC103697518 | | -8.75927 | 1.252178 | 2.65E-12 | | 5.49E-11 | Protein_coding | | | | XP_008777614.1 | | linoleate 9S-lipoxygenase 6-like | |  |

Table S11: Cation transport differentially expressed genes in date palm treatment groups

| Differentially expressed genes involved in cation transport in the Fungi treated group | | | | | | | |
| --- | --- | --- | --- | --- | --- | --- | --- |
| Gene_Symbol | log2FoldChange | lfcSE | stat | pvalue | Type | Protein_ID | Product |
| LOC103701573 | -1.84355 | 0.143675 | -12.8314 | 1.09E-37 | protein_coding | XP_008781916.2 | cation transporter HKT8-like |
| LOC103701574 | -0.90757 | 0.295732 | -3.06888 | 0.002149 | protein_coding | XP_008781917.1 | cation transporter HKT8-like |
| LOC103701575 | -0.82683 | 0.852706 | -0.96965 | 0.332219 | protein_coding | XP_026658222.1 | probable cation transporter HKT6 |
| LOC103713118 | -0.37141 | 0.335249 | -1.10787 | 0.26792 | protein_coding | XP_008798150.1 | cation transporter HKT1-like |
| LOC103715558 | 3.487624 | 0.858201 | 4.06388 | 4.83E-05 | protein_coding | XP_026663658.1 | cation transporter HKT1-like |
| Differentially expressed genes involved in cation transport in the Salt treated group | | | | | | | |
| Gene_Symbol | log2FoldChange | lfcSE | stat | pvalue | Type | Protein_ID | Product |
| LOC103701573 | -0.75616 | 0.199488 | -3.79049 | 0.00015 | protein_coding | XP_008781916.2 | cation transporter HKT8-like |
| LOC103701574 | -1.9617 | 0.343424 | -5.71217 | 1.12E-08 | protein_coding | XP_008781917.1 | cation transporter HKT8-like |
| LOC103701575 | -5.94915 | 1.678915 | -3.54345 | 0.000395 | protein_coding | XP_026658222.1 | probable cation transporter HKT6 |
| LOC103713118 | -3.57069 | 0.474381 | -7.52704 | 5.19E-14 | protein_coding | XP_008798150.1 | cation transporter HKT1-like |
| LOC103715558 | -1.02318 | 1.750128 | -0.58463 | 0.558796 | protein_coding | XP_026663658.1 | cation transporter HKT1-like |
| Differentially expressed genes involved in cation transport in the Fungi+Salt treated group | | | | | | | |
| Gene_Symbol | log2FoldChange | lfcSE | stat | pvalue | Type | Protein_ID | product |
| LOC103701573 | -2.62008 | 0.145529 | 1.82E-72 | 8.17E-70 | protein_coding | XP_008781916.2 | cation transporter HKT8-like |
| LOC103701574 | -3.35288 | 0.338811 | 4.33E-23 | 2.31E-21 | protein_coding | XP_008781917.1 | cation transporter HKT8-like |
| LOC103701575 | -5.72675 | 1.569111 | 0.000263 | 0.001434 | protein_coding | XP_026658222.1 | probable cation transporter HKT6 |
| LOC103713118 | -3.91641 | 0.448407 | 2.46E-18 | 8.88E-17 | protein_coding | XP_008798150.1 | cation transporter HKT1-like |
| LOC103715558 | -4.23891 | 2.633119 | 0.107432 | 0.224148 | protein_coding | XP_026663658.1 | cation transporter HKT1-like |

Table S12: Sodium/Hydrogen exchange differentially expressed genes in the date palm treatment groups

| Characteristics of differentially expressed genes involved in Sodium/Hydrogen exchange in the Fungi treated group | | | | | | | |
| --- | --- | --- | --- | --- | --- | --- | --- |
| Gene_Symbol | log2FoldChange | lfcSE | stat | pvalue | Type | Protein_ID | Product |
| LOC103723229 | -0.70228 | 0.302743 | -2.31971 | 0.020357 | protein_coding | XP_026655817.1; XP_008812301.1 | sodium/hydrogen exchanger 2-like |
| LOC103722382 | -0.24926 | 0.128135 | -1.94527 | 0.051742 | protein_coding | XP_008811144.1 | sodium/hydrogen exchanger 6-like |
| LOC103714902 | -0.90091 | 0.512667 | -1.75731 | 0.078866 | protein_coding | XP_026663406.1 | sodium/hydrogen exchanger 2-like |
| LOC103713551 | -0.13846 | 0.105334 | -1.31449 | 0.188681 | protein_coding | XP_008798740.1 | sodium/hydrogen exchanger 6-like |
| LOC103713975 | 0.069701 | 0.103844 | 0.671205 | 0.50209 | protein_coding | XP_026663051.1; XP_008799262.1 | sodium/hydrogen exchanger 2 |
| LOC103722426 | 0.25829 | 0.415413 | 0.621767 | 0.534095 | protein_coding | XP_026666326.1; XP_008811204.1 | sodium/hydrogen exchanger 6-like |
| LOC103713094 | 0.062108 | 0.112781 | 0.550693 | 0.581844 | protein_coding | XP_008798100.1; XP_026662993.1 | sodium/hydrogen exchanger 8 |
| LOC103696778 | -0.15875 | 0.849798 | -0.18681 | 0.851813 | protein_coding | XP_008776714.1 | sodium/hydrogen exchanger 2-like |
| Characteristics of differentially expressed genes involved in Sodium/Hydrogen exchange in the Salt treated group | | | | | | | |
| Gene_Symbol | log2FoldChange | lfcSE | stat | pvalue | Type | Protein_ID | product |
| LOC103713094 | 1.109229 | 0.369558 | 3.001504 | 0.002686 | protein_coding | XP_008798100.1; XP_026662993.1 | sodium/hydrogen exchanger 8 |
| LOC103713551 | -0.182 | 0.226669 | -0.80292 | 0.422018 | protein_coding | XP_008798740.1 | sodium/hydrogen exchanger 6-like |
| LOC103713975 | -0.05526 | 0.136313 | -0.40539 | 0.685192 | protein_coding | XP_026663051.1; XP_008799262.1 | sodium/hydrogen exchanger 2 |
| LOC103714902 | -0.84299 | 0.799496 | -1.0544 | 0.291699 | protein_coding | XP_026663406.1 | sodium/hydrogen exchanger 2-like |
| LOC103722382 | 0.013786 | 0.372774 | 0.036983 | 0.970498 | protein_coding | XP_008811144.1 | sodium/hydrogen exchanger 6-like |
| LOC103722426 | -0.19761 | 0.396577 | -0.49828 | 0.618286 | protein_coding | XP_026666326.1; XP_008811204.1 | sodium/hydrogen exchanger 6-like |
| LOC103723229 | 0.694177 | 0.564597 | 1.229508 | 0.218881 | protein_coding | XP_026655817.1; XP_008812301.1 | sodium/hydrogen exchanger 2-like |
| LOC103696778 | 0.670013 | 0.750482 | 0.892776 | 0.371977 | protein_coding | XP_008776714.1 | sodium/hydrogen exchanger 2-like |
| Characteristics of differentially expressed genes involved in Sodium/Hydrogen exchange in the Fungi+Salt treated group | | | | | | | |
| Gene_Symbol | log2FoldChange | lfcSE | pvalue | padj | Type | Protein_ID | Product |
| LOC103713094 | 1.288242 | 0.099185 | 1.42E-38 | 1.97E-36 | protein_coding | XP_008798100.1; XP_026662993.1 | sodium/hydrogen exchanger 8 |
| LOC103713551 | 0.03449 | 0.102617 | 0.736789 | 0.846374 | protein_coding | XP_008798740.1 | sodium/hydrogen exchanger 6-like |
| LOC103713975 | -0.07136 | 0.098195 | 0.467428 | 0.638215 | protein_coding | XP_026663051.1; XP_008799262.1 | sodium/hydrogen exchanger 2 |
| LOC103714902 | -0.38857 | 0.458526 | 0.396759 | 0.57339 | protein_coding | XP_026663406.1 | sodium/hydrogen exchanger 2-like |
| LOC103722382 | 0.566269 | 0.11132 | 3.64E-07 | 3.68E-06 | protein_coding | XP_008811144.1 | sodium/hydrogen exchanger 6-like |
| LOC103722426 | 0.558869 | 0.346149 | 0.106412 | 0.222471 | protein_coding | XP_026666326.1; XP_008811204.1 | sodium/hydrogen exchanger 6-like |
| LOC103723229 | -1.60693 | 0.325453 | 7.91E-07 | 7.52E-06 | protein_coding | XP_026655817.1; XP_008812301.1 | sodium/hydrogen exchanger 2-like |
| LOC103696778 | -0.5971 | 0.815989 | 0.464319 | 0.635289 | protein_coding | XP_008776714.1 | sodium/hydrogen exchanger 2-like |

Table S13: ABC transporter differentially expressed genes in the date palm treatment groups

| Characteristics of differentially expressed ABC transporter genes in the Fungi treated group | | | | | | | | | | | |  |
| --- | --- | --- | --- | --- | --- | --- | --- | --- | --- | --- | --- | --- |
| Gene_name | log2FoldChange | lfcSE | pvalue | type | | gene_ID | | | product |  |  |  |
| LOC103705291 | -2.17644 | 0.230856 | 4.19E-21 | protein_coding | | XP_026660692.1; XP_008787206.1 | | | ABC transporter D family member 2, chloroplastic-like | | |  |
| LOC103718320 | 1.028679 | 0.14175 | 3.96E-13 | protein_coding | | XP_008805333.1; XP_008805406.1 | | | ABC transporter E family member 2-like | | |  |
| LOC103696749 | 3.011594 | 0.446049 | 1.46E-11 | protein_coding | | XP_026658224.1 | | | ABC transporter G family member 11-like | | |  |
| LOC103705162 | 1.625164 | 0.256986 | 2.55E-10 | protein_coding | | XP_008787010.1 | | | ABC transporter B family member 19-like | | |  |
| LOC103703336 | 8.635159 | 1.411671 | 9.54E-10 | protein_coding | | XP_008784383.1 | | | ABC transporter B family member 19 | | |  |
| LOC103704182 | 1.524451 | 0.270842 | 1.82E-08 | protein_coding | | XP_008785597.1 | | | ABC transporter G family member 39 | | |  |
| LOC103705647 | 2.680259 | 0.481299 | 2.57E-08 | protein_coding | | XP_026660079.1; XP_026660073.1; XP_008787683.1; XP_008787688.1 | | | ABC transporter B family member 29, chloroplastic | | |  |
| LOC103708150 | 2.594967 | 0.508017 | 3.26E-07 | protein_coding | | XP_026660591.1 | | | ABC transporter G family member 25-like | | |  |
| LOC103707342 | -1.0924 | 0.222993 | 9.64E-07 | protein_coding | | XP_026660737.1; XP_026660741.1; XP_017698344.1; XP_017698339.1; XP_008790019.1; XP_008790010.1; XP_017698341.1 | | | ABC transporter G family member 22 | | |  |
| LOC103708414 | 1.091785 | 0.227183 | 1.54E-06 | protein_coding | | XP_008791552.1 | | | ABC transporter G family member 42-like | | |  |
| LOC103710662 | 2.219021 | 0.490972 | 6.19E-06 | protein_coding | | XP_008794734.1; XP_026662087.1 | | | ABC transporter B family member 20-like | | |  |
| LOC103711386 | 6.244842 | 1.595335 | 9.06E-05 | protein_coding | | XP_008795733.1 | | | ABC transporter B family member 20-like | | |  |
| LOC103721732 | 1.913592 | 0.548361 | 0.000484 | protein_coding | | XP_008810273.1 | | | ABC transporter I family member 19-like | | |  |
| LOC103697782 | 4.724913 | 1.429424 | 0.000948 | protein_coding | | XP_008777932.2 | | | ABC transporter G family member 6 | | |  |
| LOC103698293 | 2.071896 | 0.655305 | 0.001568 | protein_coding | | XP_008778513.1 | | | ABC transporter F family member 3 | | |  |
| LOC103701030 | -2.01795 | 0.732737 | 0.005887 | protein_coding | | XP_008781186.1 | | | ABC transporter I family member 20 | | |  |
| LOC103701308 | -0.58956 | 0.161997 | 0.000273 | protein_coding | | XP_017696858.1 | | | ABC transporter G family member 25 | | |  |
| LOC103701335 | -0.51279 | 0.143146 | 0.000341 | protein_coding | | XP_008781581.1 | | | ABC transporter D family member 1-like | | |  |
| LOC103702232 | 0.284276 | 0.079556 | 0.000353 | protein_coding | | XP_026658438.1 | | | ABC transporter I family member 6, chloroplastic-like | | |  |
| LOC103702171 | -0.59475 | 0.170229 | 0.000476 | protein_coding | | XP_026658443.1; XP_008782716.1 | | | ABC transporter B family member 25, mitochondrial-like | | |  |
| LOC103702532 | 0.799458 | 0.231836 | 0.000564 | protein_coding | | XP_008783221.1; XP_008783223.1 | | | ABC transporter E family member 2-like | | |  |
| LOC103702624 | -0.76029 | 0.220534 | 0.000566 | protein_coding | | XP_008783350.1; XP_008783348.1; XP_026658634.1; XP_008783347.1; XP_026658635.1 | | | ABC transporter D family member 1-like | | |  |
| LOC103702981 | 0.388762 | 0.113273 | 0.000599 | protein_coding | | XP_017697293.1; XP_008783877.1; XP_026658763.1; XP_008783876.1 | | | ABC transporter D family member 2, chloroplastic-like | | |  |
| LOC103703562 | -0.42323 | 0.12464 | 0.000685 | protein_coding | | XP_008784678.1 | | | putative ABC transporter C family member 15 | | |  |
| LOC103704782 | 0.647132 | 0.190803 | 0.000695 | protein_coding | | XP_008786438.2 | | | ABC transporter G family member 45-like | | |  |
| LOC103706998 | 0.484709 | 0.143131 | 0.000708 | protein_coding | | XP_008789538.2 | | | ABC transporter B family member 1-like | | |  |
| LOC103707002 | -0.88964 | 0.264126 | 0.000756 | protein_coding | | XP_008789541.1; XP_026660417.1 | | | ABC transporter F family member 1-like | | |  |
| LOC103707665 | -0.2516 | 0.07827 | 0.001306 | protein_coding | | XP_017698423.1 | | | ABC transporter B family member 9-like | | |  |
| LOC103707953 | 0.610678 | 0.194035 | 0.001648 | protein_coding | | XP_008790912.2 | | | ABC transporter G family member 11-like | | |  |
| LOC103707982 | -0.93589 | 0.307367 | 0.002328 | protein_coding | | XP_008790942.1 | | | ABC transporter A family member 1 | | |  |
| LOC103708206 | 0.624175 | 0.205428 | 0.002378 | protein_coding | | XP_008791250.1 | | | ABC transporter G family member 10 | | |  |
| LOC103708369 | -0.26009 | 0.087784 | 0.003048 | protein_coding | | XP_008791489.1 | | | ABC transporter G family member 11-like | | |  |
| LOC103708769 | -0.2577 | 0.096867 | 0.007807 | protein_coding | | XP_026661090.1; XP_008792064.2 | | | ABC transporter C family member 3-like | | |  |
| LOC103708768 | -0.28755 | 0.110089 | 0.009002 | protein_coding | | XP_008792061.1; XP_008792063.1 | | | ABC transporter C family member 3-like | | |  |
| LOC103709706 | 0.713327 | 0.27585 | 0.009712 | protein_coding | | XP_008793410.1 | | | ABC transporter B family member 25, mitochondrial | | |  |
| LOC103709774 | 3.092359 | 1.237337 | 0.012447 | protein_coding | | XP_008793496.1 | | | ABC transporter A family member 7-like | | |  |
| LOC103709796 | 0.356193 | 0.152849 | 0.019787 | protein_coding | | XP_026661485.1 | | | ABC transporter A family member 7-like | | |  |
| LOC103709922 | 0.659631 | 0.291766 | 0.02377 | protein_coding | | XP_008793680.2 | | | ABC transporter G family member 35-like | | |  |
| LOC103709907 | 0.516267 | 0.229053 | 0.024201 | protein_coding | | XP_008793658.2 | | | ABC transporter F family member 4-like | | |  |
| LOC103710863 | 0.385304 | 0.181685 | 0.033945 | protein_coding | | XP_008794995.1 | | | ABC transporter C family member 10-like | | |  |
| LOC103710876 | 0.322618 | 0.152515 | 0.034402 | protein_coding | | XP_008795011.1 | | | ABC transporter G family member 8-like | | |  |
| LOC103711124 | -0.32784 | 0.15829 | 0.038347 | protein_coding | | XP_008795363.2; XP_017699281.1 | | | ABC transporter B family member 28 | | |  |
| LOC103711178 | 0.55367 | 0.273711 | 0.043091 | protein_coding | | XP_008795450.1 | | | ABC transporter G family member 36-like | | |  |
| LOC103711867 | 0.772963 | 0.383225 | 0.043696 | protein_coding | | XP_008796398.1; XP_008796397.1 | | | ABC transporter C family member 5 | | |  |
| LOC103712996 | -0.21033 | 0.105015 | 0.045192 | protein_coding | | XP_008797958.1 | | | ABC transporter G family member 14-like | | |  |
| LOC103713658 | 0.217103 | 0.11095 | 0.050374 | protein_coding | | XP_008798883.1 | | | ABC transporter B family member 21-like | | |  |
| LOC103714551 | 0.697179 | 0.362167 | 0.054226 | protein_coding | | XP_026663252.1 | | | ABC transporter G family member 28 | | |  |
| LOC103714592 | 0.465534 | 0.244161 | 0.056563 | protein_coding | | XP_008800115.1 | | | ABC transporter B family member 2-like | | |  |
| LOC103714881 | 0.326194 | 0.171377 | 0.056992 | protein_coding | | XP_008800546.1; XP_008800545.1 | | | ABC transporter F family member 1-like | | |  |
| LOC103714888 | -0.19844 | 0.106162 | 0.061597 | protein_coding | | XP_008800555.1 | | | ABC transporter B family member 1-like | | |  |
| LOC103715086 | 0.309854 | 0.167722 | 0.064684 | protein_coding | | XP_026663485.1; XP_008800825.1; XP_008800828.1 | | | ABC transporter C family member 14-like | | |  |
| LOC103715579 | 2.126979 | 1.161521 | 0.067071 | protein_coding | | XP_008801483.1; XP_008801482.1; XP_008801484.1 | | | ABC transporter E family member 2 | | |  |
| LOC103716937 | -0.12989 | 0.081513 | 0.111054 | protein_coding | | XP_008803363.1 | | | ABC transporter G family member 23 | | |  |
| LOC103717193 | 0.781679 | 0.501234 | 0.118876 | protein_coding | | XP_008803705.1; XP_008803704.1; XP_017700699.1; XP_026664281.1; XP_008803706.1 | | | ABC transporter C family member 13 | | |  |
| LOC103717446 | 0.441156 | 0.287103 | 0.124398 | protein_coding | | XP_008804056.1 | | | ABC transporter G family member 31 | | |  |
| LOC103717750 | -0.23188 | 0.152147 | 0.127489 | protein_coding | | XP_008804465.1 | | | ABC transporter B family member 19-like | | |  |
| LOC103717842 | -1.5102 | 1.02779 | 0.141733 | protein_coding | | XP_008804599.1 | | | ABC transporter G family member 42-like | | |  |
| LOC103718564 | -1.09082 | 0.762347 | 0.152466 | protein_coding | | XP_008805673.1 | | | ABC transporter G family member 14-like | | |  |
| LOC113463567 | -0.65981 | 0.47384 | 0.163778 | protein_coding | | XP_026665254.1 | | | ABC transporter G family member 7-like | | |  |
| LOC103719818 | -0.82272 | 0.594151 | 0.166145 | protein_coding | | XP_026665321.1; XP_008807453.1 | | | ABC transporter B family member 26, chloroplastic | | |  |
| LOC103719895 | -0.40086 | 0.289502 | 0.166157 | protein_coding | | XP_008807599.1 | | | ABC transporter F family member 4-like | | |  |
| LOC103719950 | 1.067016 | 0.894778 | 0.233068 | protein_coding | | XP_008807660.1; XP_008807661.1; XP_026665359.1; XP_026665360.1; XP_017701396.1 | | | ABC transporter D family member 1-like | | |  |
| LOC103720584 | -0.4158 | 0.355941 | 0.242733 | protein_coding | | XP_008808572.1 | | | ABC transporter A family member 7-like | | |  |
| LOC103720840 | 0.265156 | 0.234122 | 0.257401 | protein_coding | | XP_026665699.1; XP_008808988.1 | | | ABC transporter I family member 1 | | |  |
| LOC103721215 | 0.16356 | 0.147486 | 0.267436 | protein_coding | | XP_026665872.1; XP_008809553.1; XP_026665871.1; XP_026665870.1 | | | ABC transporter I family member 11, chloroplastic | | |  |
| LOC103721245 | 0.495412 | 0.460045 | 0.281535 | protein_coding | | XP_008809589.1; XP_008809590.1 | | | ABC transporter I family member 10 | | |  |
| LOC103721359 | -0.13955 | 0.133066 | 0.294312 | protein_coding | | XP_008809753.2 | | | putative ABC transporter B family member 8 | | |  |
| LOC103721413 | -0.10998 | 0.107916 | 0.30814 | protein_coding | | XP_008809834.1 | | | ABC transporter B family member 9-like | | |  |
| LOC103721783 | 0.853845 | 0.844474 | 0.31197 | protein_coding | | XP_026666095.1 | | | ABC transporter G family member 41-like | | |  |
| LOC103721797 | 0.106134 | 0.114503 | 0.353972 | protein_coding | | XP_017701829.1 | | | ABC transporter B family member 4-like | | |  |
| LOC103721895 | -0.13931 | 0.150871 | 0.3558 | protein_coding | | XP_008810494.1 | | | ABC transporter G family member 11-like | | |  |
| LOC103721916 | -0.29367 | 0.318498 | 0.356508 | protein_coding | | XP_008810525.1 | | | ABC transporter G family member 6-like | | |  |
| LOC103722178 | -0.21368 | 0.244246 | 0.381663 | protein_coding | | XP_008810860.1 | | | ABC transporter I family member 6, chloroplastic-like | | |  |
| LOC103722225 | 0.511491 | 0.609797 | 0.401587 | protein_coding | | XP_008810921.1 | | | ABC transporter G family member 5 | | |  |
| LOC103722707 | -0.08308 | 0.105466 | 0.430838 | protein_coding | | XP_008811580.1 | | | ABC transporter F family member 5-like | | |  |
| LOC103722812 | -0.19366 | 0.248459 | 0.435714 | protein_coding | | XP_008811726.1 | | | ABC transporter C family member 8 | | |  |
| LOC103722973 | -0.10357 | 0.136028 | 0.446425 | protein_coding | | XP_017702116.1 | | | ABC transporter G family member 20-like | | |  |
| LOC103722985 | 0.135825 | 0.179499 | 0.449233 | protein_coding | | XP_008811977.1 | | | ABC transporter G family member 7 | | |  |
| LOC103723003 | 0.10338 | 0.138031 | 0.453879 | protein_coding | | XP_026655731.1; XP_026655729.1; XP_026655732.1; XP_008812003.1; XP_026655730.1; XP_026655733.1; XP_026655728.1 | | | ABC transporter F family member 4-like | | |  |
| LOC103723467 | -0.1132 | 0.157392 | 0.472005 | protein_coding | | XP_008812601.1; XP_026655901.1; XP_026655900.1; XP_008812600.1 | | | ABC transporter C family member 14-like | | |  |
| LOC103723478 | 0.134154 | 0.188586 | 0.476856 | protein_coding | | XP_017702224.1 | | | ABC transporter G family member 14-like | | |  |
| LOC103723484 | 0.192455 | 0.287036 | 0.502546 | protein_coding | | XP_026655914.1 | | | ABC transporter C family member 2 | | |  |
| LOC103723916 | -0.18977 | 0.289614 | 0.512314 | protein_coding | | XP_008813230.1 | | | ABC transporter I family member 6, chloroplastic-like | | |  |
| LOC103724353 | -0.05632 | 0.095846 | 0.556794 | protein_coding | | XP_026656316.1 | | | ABC transporter G family member 41-like | | |  |
| LOC103695628 | 0.773427 | 1.329872 | 0.56085 | protein_coding | | XP_008775221.1; XP_008775222.1; XP_008775219.1; XP_008775220.1 | | | ABC transporter C family member 2-like | | |  |
| LOC103696134 | -0.06177 | 0.111708 | 0.580276 | protein_coding | | XP_026656587.1; XP_026656588.1; XP_026656586.1 | | | ABC transporter G family member 14-like | | |  |
| LOC103696452 | 0.122539 | 0.225324 | 0.586554 | protein_coding | | XP_008776307.1 | | | ABC transporter A family member 2-like | | |  |
| LOC103696478 | 0.505593 | 1.168358 | 0.665205 | protein_coding | | XP_026656709.1 | | | ABC transporter G family member 17-like | | |  |
| LOC103696855 | 0.125725 | 0.322551 | 0.696696 | protein_coding | | XP_008776799.1 | | | ABC transporter G family member 28-like | | |  |
| LOC103696929 | -0.20808 | 0.594746 | 0.726447 | protein_coding | | XP_008776879.1; XP_008776880.1 | | | ABC transporter G family member 3 | | |  |
| LOC103696952 | -0.05116 | 0.214018 | 0.811077 | protein_coding | | XP_026656870.1; XP_008776904.1 | | | ABC transporter B family member 9-like | | |  |
| LOC103697148 | 0.07627 | 0.327661 | 0.81594 | protein_coding | | XP_008777170.1 | | | ABC transporter B family member 2-like | | |  |
| LOC103697353 | -0.05561 | 0.380972 | 0.883943 | protein_coding | | XP_008777416.1 | | | protein ABC transporter 1, mitochondrial | | |  |
| LOC103697619 | -0.032 | 0.225331 | 0.887057 | protein_coding | | XP_008777748.1 | | | putative ABC transporter C family member 15 | | |  |
| LOC103697840 | 0.039547 | 0.286331 | 0.890148 | protein_coding | | XP_008778003.1; XP_008778002.1 | | | ABC transporter C family member 5-like | | |  |
| LOC103698270 | 0.018195 | 0.154081 | 0.906 | protein_coding | | XP_008778485.1 | | | ABC transporter B family member 25 | | |  |
| LOC103698927 | 0.01826 | 0.207438 | 0.929857 | protein_coding | | XP_026657548.1 | | | ABC transporter G family member 41-like | | |  |
| Characteristics of differentially expressed ABC transporter genes in the Fungi+Salt treated group | | | | | | | | | | | | |
| gene_name | log2FoldChange | lfcSE | pvalue | padj | type | | gene_ID | product | | | | |
| LOC103705291 | 0.353723 | 0.126322 | 0.005108 | 0.018944 | protein_coding | | XP_026660692.1; XP_008787206.1 | ABC transporter D family member 2, chloroplastic-like | | | | |
| LOC103718320 | 0.200167 | 0.126279 | 0.11294 | 0.233063 | protein_coding | | XP_008805333.1; XP_008805406.1 | ABC transporter E family member 2-like | | | | |
| LOC103703336 | -1.41675 | 0.336328 | 2.53E-05 | 0.000178 | protein_coding | | XP_008784383.1 | ABC transporter B family member 19 | | | | |
| LOC103704182 | 0.700427 | 0.163342 | 0.000018 | 0.00013 | protein_coding | | XP_008785597.1 | ABC transporter G family member 39 | | | | |
| LOC103705647 | -0.03959 | 0.268705 | 0.882877 | 0.936208 | protein_coding | | XP_026660079.1; XP_026660073.1; XP_008787683.1; XP_008787688.1 | ABC transporter B family member 29, chloroplastic | | | | |
| LOC103708150 | 1.089099 | 1.798997 | 0.544918 | 0.703735 | protein_coding | | XP_026660591.1 | ABC transporter G family member 25-like | | | | |
| LOC103707342 | -2.14926 | 1.222512 | 0.078735 | 0.176134 | protein_coding | | XP_026660737.1; XP_026660741.1; XP_017698344.1; XP_017698339.1; XP_008790019.1; XP_008790010.1; XP_017698341.1 | ABC transporter G family member 22 | | | | |
| LOC103708414 | -0.89667 | 0.142108 | 2.79E-10 | 4.52E-09 | protein_coding | | XP_008791552.1 | ABC transporter G family member 42-like | | | | |
| LOC103710662 | 0.283644 | 0.121351 | 0.019419 | 0.057433 | protein_coding | | XP_008794734.1; XP_026662087.1 | ABC transporter B family member 20-like | | | | |
| LOC103711386 | 0.214159 | 0.083998 | 0.010785 | 0.035374 | protein_coding | | XP_008795733.1 | ABC transporter B family member 20-like | | | | |
| LOC103721732 | 0.143291 | 0.142176 | 0.313529 | 0.486648 | protein_coding | | XP_008810273.1 | ABC transporter I family member 19-like | | | | |
| LOC103697782 | -1.61337 | 0.725715 | 0.026206 | 0.073266 | protein_coding | | XP_008777932.2 | ABC transporter G family member 6 | | | | |
| LOC103698293 | 0.064054 | 0.092337 | 0.487874 | 0.65505 | protein_coding | | XP_008778513.1 | ABC transporter F family member 3 | | | | |
| LOC103701011 | 3.090917 | 2.141854 | 0.148991 | 0.286389 | protein_coding | | XP_026657974.1 | ABC transporter G family member 51-like | | | | |
| LOC103701030 | 0.451593 | 0.308118 | 0.142744 | 0.277225 | protein_coding | | XP_008781186.1 | ABC transporter I family member 20 | | | | |
| LOC103701308 | 1.247681 | 0.253776 | 8.81E-07 | 8.30E-06 | protein_coding | | XP_017696858.1 | ABC transporter G family member 25 | | | | |
| LOC103701335 | 0.457254 | 0.091223 | 5.37E-07 | 5.25E-06 | protein_coding | | XP_008781581.1 | ABC transporter D family member 1-like | | | | |
| LOC103702232 | -0.04561 | 0.227749 | 0.84129 | 0.911966 | protein_coding | | XP_026658438.1 | ABC transporter I family member 6, chloroplastic-like | | | | |
| LOC103702171 | -0.17364 | 0.357634 | 0.6273 | 0.770245 | protein_coding | | XP_026658443.1; XP_008782716.1 | ABC transporter B family member 25, mitochondrial-like | | | | |
| LOC103702532 | 0.058076 | 0.084914 | 0.494012 | 0.660149 | protein_coding | | XP_008783221.1; XP_008783223.1 | ABC transporter E family member 2-like | | | | |
| LOC103702624 | -0.5848 | 0.147757 | 7.56E-05 | 0.000472 | protein_coding | | XP_008783350.1; XP_008783348.1; XP_026658634.1; XP_008783347.1; XP_026658635.1 | ABC transporter D family member 1-like | | | | |
| LOC103702981 | -3.078 | 0.463449 | 3.11E-11 | 5.75E-10 | protein_coding | | XP_017697293.1; XP_008783877.1; XP_026658763.1; XP_008783876.1 | ABC transporter D family member 2, chloroplastic-like | | | | |
| LOC103703562 | 0.761282 | 0.245224 | 0.001906 | 0.00816 | protein_coding | | XP_008784678.1 | putative ABC transporter C family member 15 | | | | |
| LOC103704020 | -0.55888 | 1.49857 | 0.709192 | 0.827262 | protein_coding | | XP_026659221.1 | ABC transporter G family member 51 | | | | |
| LOC103704782 | 0.434285 | 0.087685 | 7.31E-07 | 6.99E-06 | protein_coding | | XP_008786438.2 | ABC transporter G family member 45-like | | | | |
| LOC103706998 | -0.57767 | 0.246548 | 0.019128 | 0.056708 | protein_coding | | XP_008789538.2 | ABC transporter B family member 1-like | | | | |
| LOC103707002 | -0.01453 | 0.064644 | 0.822182 | 0.900523 | protein_coding | | XP_008789541.1; XP_026660417.1 | ABC transporter F family member 1-like | | | | |
| LOC103707665 | 3.133462 | 1.712681 | 0.067315 | 0.155559 | protein_coding | | XP_017698423.1 | ABC transporter B family member 9-like | | | | |
| LOC103707953 | 8.344296 | 1.279045 | 6.85E-11 | 1.21E-09 | protein_coding | | XP_008790912.2 | ABC transporter G family member 11-like | | | | |
| LOC103707982 | -0.33364 | 0.069745 | 1.72E-06 | 1.53E-05 | protein_coding | | XP_008790942.1 | ABC transporter A family member 1 | | | | |
| LOC113462773 | -0.80541 | 1.734938 | 0.642482 | 0.781705 | protein_coding | | XP_026660790.1 | ABC transporter A family member 1-like | | | | |
| LOC103708206 | -3.36072 | 0.767731 | 0.000012 | 9.03E-05 | protein_coding | | XP_008791250.1 | ABC transporter G family member 10 | | | | |
| LOC103708369 | 1.060261 | 0.152026 | 3.08E-12 | 6.34E-11 | protein_coding | | XP_008791489.1 | ABC transporter G family member 11-like | | | | |
| LOC103708769 | 1.154138 | 0.896244 | 0.197833 | 0.352371 | protein_coding | | XP_026661090.1; XP_008792064.2 | ABC transporter C family member 3-like | | | | |
| LOC103708768 | 1.81513 | 0.087828 | 6.89E-95 | 6.32E-92 | protein_coding | | XP_008792061.1; XP_008792063.1 | ABC transporter C family member 3-like | | | | |
| LOC103709706 | -0.09479 | 0.144805 | 0.512743 | 0.675993 | protein_coding | | XP_008793410.1 | ABC transporter B family member 25, mitochondrial | | | | |
| LOC103709774 | 0.423472 | 0.27079 | 0.117854 | 0.240733 | protein_coding | | XP_008793496.1 | ABC transporter A family member 7-like | | | | |
| LOC103709796 | -1.22943 | 0.144678 | 1.93E-17 | 6.47E-16 | protein_coding | | XP_026661485.1 | ABC transporter A family member 7-like | | | | |
| LOC103709922 | 2.189844 | 0.52147 | 2.68E-05 | 0.000187 | protein_coding | | XP_008793680.2 | ABC transporter G family member 35-like | | | | |
| LOC103709907 | 0.584986 | 0.164592 | 0.000379 | 0.001985 | protein_coding | | XP_008793658.2 | ABC transporter F family member 4-like | | | | |
| LOC103710863 | 1.854461 | 0.168053 | 2.59E-28 | 1.92E-26 | protein_coding | | XP_008794995.1 | ABC transporter C family member 10-like | | | | |
| LOC103710876 | 4.010471 | 2.860911 | 0.16097 | 0.303526 | protein_coding | | XP_008795011.1 | ABC transporter G family member 8-like | | | | |
| LOC103711124 | -0.34872 | 0.151341 | 0.021212 | 0.061829 | protein_coding | | XP_008795363.2; XP_017699281.1 | ABC transporter B family member 28 | | | | |
| LOC103711178 | 0.976283 | 0.6521 | 0.134358 | 0.265457 | protein_coding | | XP_008795450.1 | ABC transporter G family member 36-like | | | | |
| LOC103711867 | 1.255597 | 0.125438 | 1.38E-23 | 7.71E-22 | protein_coding | | XP_008796398.1; XP_008796397.1 | ABC transporter C family member 5 | | | | |
| LOC103712869 | -0.62655 | 2.799567 | 0.82291 | 0.901011 | protein_coding | | XP_026662656.1 | ABC transporter G family member 26 | | | | |
| LOC103712996 | -0.60791 | 0.635897 | 0.33908 | 0.515398 | protein_coding | | XP_008797958.1 | ABC transporter G family member 14-like | | | | |
| LOC103713658 | -2.38004 | 0.877537 | 0.006684 | 0.023817 | protein_coding | | XP_008798883.1 | ABC transporter B family member 21-like | | | | |
| LOC103713650 | 0.033204 | 0.103962 | 0.749438 | 0.854605 | protein_coding | | XP_017699893.1 | ABC transporter B family member 21-like | | | | |
| LOC103714551 | -1.02044 | 0.237892 | 1.79E-05 | 0.00013 | protein_coding | | XP_026663252.1 | ABC transporter G family member 28 | | | | |
| LOC103714592 | -0.58424 | 0.158051 | 0.000219 | 0.001221 | protein_coding | | XP_008800115.1 | ABC transporter B family member 2-like | | | | |
| LOC103714881 | 1.529578 | 0.108426 | 3.43E-45 | 6.52E-43 | protein_coding | | XP_008800546.1; XP_008800545.1 | ABC transporter F family member 1-like | | | | |
| LOC103714888 | -1.41674 | 0.328664 | 1.63E-05 | 0.000119 | protein_coding | | XP_008800555.1 | ABC transporter B family member 1-like | | | | |
| LOC103715086 | -0.30655 | 0.225964 | 0.174893 | 0.322318 | protein_coding | | XP_026663485.1; XP_008800825.1; XP_008800828.1 | ABC transporter C family member 14-like | | | | |
| LOC103715579 | -0.24993 | 0.084581 | 0.003128 | 0.012468 | protein_coding | | XP_008801483.1; XP_008801482.1; XP_008801484.1 | ABC transporter E family member 2 | | | | |
| LOC103716937 | -2.15593 | 0.499458 | 1.58E-05 | 0.000116 | protein_coding | | XP_008803363.1 | ABC transporter G family member 23 | | | | |
| LOC103717193 | -0.16091 | 0.07896 | 0.041559 | 0.106374 | protein_coding | | XP_008803705.1; XP_008803704.1; XP_017700699.1; XP_026664281.1; XP_008803706.1 | ABC transporter C family member 13 | | | | |
| LOC103717446 | -3.59213 | 0.265172 | 8.32E-42 | 1.32E-39 | protein_coding | | XP_008804056.1 | ABC transporter G family member 31 | | | | |
| LOC103717750 | -3.21697 | 0.843716 | 0.000137 | 0.000805 | protein_coding | | XP_008804465.1 | ABC transporter B family member 19-like | | | | |
| LOC103717842 | -0.08315 | 0.158747 | 0.600423 | 0.750017 | protein_coding | | XP_008804599.1 | ABC transporter G family member 42-like | | | | |
| LOC103718564 | -1.52867 | 0.55403 | 0.005795 | 0.02108 | protein_coding | | XP_008805673.1 | ABC transporter G family member 14-like | | | | |
| LOC113463567 | 0.330163 | 0.233679 | 0.157687 | 0.298783 | protein_coding | | XP_026665254.1 | ABC transporter G family member 7-like | | | | |
| LOC103719818 | 0.385763 | 0.159626 | 0.015663 | 0.048067 | protein_coding | | XP_026665321.1; XP_008807453.1 | ABC transporter B family member 26, chloroplastic | | | | |
| LOC103719895 | -3.57418 | 0.885653 | 5.45E-05 | 0.000354 | protein_coding | | XP_008807599.1 | ABC transporter F family member 4-like | | | | |
| LOC103719950 | 0.503317 | 0.111668 | 6.57E-06 | 5.24E-05 | protein_coding | | XP_008807660.1; XP_008807661.1; XP_026665359.1; XP_026665360.1; XP_017701396.1 | ABC transporter D family member 1-like | | | | |
| LOC103720584 | 0.321366 | 0.153478 | 0.03627 | 0.095513 | protein_coding | | XP_008808572.1 | ABC transporter A family member 7-like | | | | |
| LOC103720840 | 0.640302 | 1.325574 | 0.629069 | 0.771789 | protein_coding | | XP_026665699.1; XP_008808988.1 | ABC transporter I family member 1 | | | | |
| LOC103721215 | -0.10093 | 0.192985 | 0.600987 | 0.750394 | protein_coding | | XP_026665872.1; XP_008809553.1; XP_026665871.1; XP_026665870.1 | ABC transporter I family member 11, chloroplastic | | | | |
| LOC103721245 | 0.474898 | 0.242132 | 0.049842 | 0.12294 | protein_coding | | XP_008809589.1; XP_008809590.1 | ABC transporter I family member 10 | | | | |
| LOC103721359 | -0.38545 | 0.17084 | 0.024057 | 0.068253 | protein_coding | | XP_008809753.2 | putative ABC transporter B family member 8 | | | | |
| LOC103721413 | 0.182488 | 0.236305 | 0.439964 | 0.613978 | protein_coding | | XP_008809834.1 | ABC transporter B family member 9-like | | | | |
| LOC103721783 | -0.23902 | 0.101514 | 0.018545 | 0.05528 | protein_coding | | XP_026666095.1 | ABC transporter G family member 41-like | | | | |
| LOC103721797 | 0.405075 | 0.136112 | 0.00292 | 0.011748 | protein_coding | | XP_017701829.1 | ABC transporter B family member 4-like | | | | |
| LOC103721895 | 1.46207 | 0.220465 | 3.32E-11 | 6.10E-10 | protein_coding | | XP_008810494.1 | ABC transporter G family member 11-like | | | | |
| LOC103721916 | 1.391495 | 0.180429 | 1.24E-14 | 3.21E-13 | protein_coding | | XP_008810525.1 | ABC transporter G family member 6-like | | | | |
| LOC103722178 | 0.306648 | 0.172298 | 0.075116 | 0.16963 | protein_coding | | XP_008810860.1 | ABC transporter I family member 6, chloroplastic-like | | | | |
| LOC103722225 | -1.09061 | 0.880663 | 0.215568 | 0.375038 | protein_coding | | XP_008810921.1 | ABC transporter G family member 5 | | | | |
| LOC103722707 | 0.525858 | 0.142293 | 0.000219 | 0.001224 | protein_coding | | XP_008811580.1 | ABC transporter F family member 5-like | | | | |
| LOC103722812 | 2.932231 | 0.973226 | 0.002588 | 0.010589 | protein_coding | | XP_008811726.1 | ABC transporter C family member 8 | | | | |
| LOC103722973 | 2.257331 | 1.40754 | 0.108771 | 0.226408 | protein_coding | | XP_017702116.1 | ABC transporter G family member 20-like | | | | |
| LOC103722985 | 0.067178 | 0.172812 | 0.697472 | 0.818539 | protein_coding | | XP_008811977.1 | ABC transporter G family member 7 | | | | |
| LOC103723003 | 0.379943 | 1.69136 | 0.822261 | 0.900544 | protein_coding | | XP_026655731.1; XP_026655729.1; XP_026655732.1; XP_008812003.1; XP_026655730.1; XP_026655733.1; XP_026655728.1 | ABC transporter F family member 4-like | | | | |
| LOC103723467 | 0.362091 | 0.142776 | 0.01121 | 0.036515 | protein_coding | | XP_008812601.1; XP_026655901.1; XP_026655900.1; XP_008812600.1 | ABC transporter C family member 14-like | | | | |
| LOC103723478 | 0.447042 | 0.253977 | 0.078379 | 0.17544 | protein_coding | | XP_017702224.1 | ABC transporter G family member 14-like | | | | |
| LOC103723484 | -0.25664 | 0.088687 | 0.003806 | 0.014792 | protein_coding | | XP_026655914.1 | ABC transporter C family member 2 | | | | |
| LOC103723916 | 0.416532 | 0.274766 | 0.129532 | 0.258088 | protein_coding | | XP_008813230.1 | ABC transporter I family member 6, chloroplastic-like | | | | |
| LOC103724353 | -0.15742 | 0.134981 | 0.24352 | 0.409109 | protein_coding | | XP_026656316.1 | ABC transporter G family member 41-like | | | | |
| LOC103695628 | 0.089348 | 0.066964 | 0.182117 | 0.331822 | protein_coding | | XP_008775221.1; XP_008775222.1; XP_008775219.1; XP_008775220.1 | ABC transporter C family member 2-like | | | | |
| LOC103696114 | 3.544518 | 3.904691 | 0.364006 | 0.541375 | protein_coding | | XP_026656577.1 | ABC transporter G family member 36-like | | | | |
| LOC103696134 | -1.40114 | 1.30989 | 0.284769 | 0.455389 | protein_coding | | XP_026656587.1; XP_026656588.1; XP_026656586.1 | ABC transporter G family member 14-like | | | | |
| LOC103696456 | 0.429671 | 0.137138 | 0.00173 | 0.007499 | protein_coding | | XP_008776318.1 | ABC transporter A family member 7-like | | | | |
| LOC103696452 | -0.43811 | 0.151133 | 0.003745 | 0.014588 | protein_coding | | XP_008776307.1 | ABC transporter A family member 2-like | | | | |
| LOC103696478 | 0.685409 | 0.983208 | 0.485731 | 0.653403 | protein_coding | | XP_026656709.1 | ABC transporter G family member 17-like | | | | |
| LOC103696677 | 4.186614 | 3.38922 | 0.216729 | 0.376495 | protein_coding | | XP_008776585.3 | ABC transporter G family member 45-like | | | | |
| LOC103696855 | -0.86361 | 0.240199 | 0.000324 | 0.001726 | protein_coding | | XP_008776799.1 | ABC transporter G family member 28-like | | | | |
| LOC103696929 | -0.50289 | 0.111319 | 6.26E-06 | 5.01E-05 | protein_coding | | XP_008776879.1; XP_008776880.1 | ABC transporter G family member 3 | | | | |
| LOC103696952 | -0.64486 | 0.702711 | 0.358786 | 0.53619 | protein_coding | | XP_026656870.1; XP_008776904.1 | ABC transporter B family member 9-like | | | | |
| LOC103697148 | 0.401042 | 0.271319 | 0.139376 | 0.272477 | protein_coding | | XP_008777170.1 | ABC transporter B family member 2-like | | | | |
| LOC103697353 | -0.03754 | 0.327278 | 0.908684 | 0.950883 | protein_coding | | XP_008777416.1 | protein ABC transporter 1, mitochondrial | | | | |
| LOC103697619 | 1.0295 | 0.709673 | 0.146872 | 0.283313 | protein_coding | | XP_008777748.1 | putative ABC transporter C family member 15 | | | | |
| LOC103697840 | -0.23116 | 0.135229 | 0.087385 | 0.191294 | protein_coding | | XP_008778003.1; XP_008778002.1 | ABC transporter C family member 5-like | | | | |
| LOC103698004 | 0.51107 | 0.09378 | 5.05E-08 | 5.87E-07 | protein_coding | | XP_017696282.1 | ABC transporter B family member 4-like | | | | |
| LOC103698270 | -0.22614 | 0.083292 | 0.006628 | 0.023645 | protein_coding | | XP_008778485.1 | ABC transporter B family member 25 | | | | |
| LOC103698927 | -0.14616 | 0.1145 | 0.201764 | 0.357714 | protein_coding | | XP_026657548.1 | ABC transporter G family member 41-like | | | | |
| LOC103699595 | 2.285829 | 0.648553 | 0.000424 | 0.002195 | protein_coding | | XP_008779838.1 | ABC transporter G family member 36-like | | | | |
| LOC103700034 | 1.739248 | 0.472647 | 0.000233 | 0.001294 | protein_coding | | XP_008780232.1 | ABC transporter G family member 44-like | | | | |
| LOC103700386 | 2.286704 | 0.458826 | 6.23E-07 | 6.03E-06 | protein_coding | | XP_017696696.1 | ABC transporter G family member 36-like | | | | |
